# Odorant receptors tuned to isothiocyanates in *Drosophila melanogaster* and their evolutionary expansion in herbivorous relatives

**DOI:** 10.1101/2024.10.08.617316

**Published:** 2024-10-11

**Authors:** Teruyuki Matsunaga, Carolina E. Reisenman, Benjamin Goldman-Huertas, Srivarsha Rajshekar, Hiromu C. Suzuki, David Tadres, Joshua Wong, Matthieu Louis, Santiago R. Ramírez, Noah K. Whiteman

**Affiliations:** 1 Department of Complexity Science and Engineering, Graduate School of Frontier Sciences, The University of Tokyo, Chiba, Japan; 2 Department of Molecular and Cell Biology, University of California Berkeley, Berkeley, CA; 3 Department of Integrative Biology, University of California Berkeley, Berkeley, CA; 4 Department of Molecular, Cellular, and Developmental Biology, University of California Santa Barbara, Santa Barbara, CA; 5 The Biochemistry, Cellular and Molecular Biology Graduate Program, The Johns Hopkins University School of Medicine; 6 Department of Evolution and Ecology, University of California Davis, Davis, CA

**Keywords:** *Drosophila melanogaster*, olfaction, isothiocyanate, odorant receptor, Or42a, *Scaptomyza flava*, herbivory, evolution, Brassicales, AlphaFold2

## Abstract

Plants release complex volatile compounds to attract mutualists, deter herbivores, and deceive pollinators. Here, we used herbivorous specialist flies that feed on mustard plants (*Scaptomyza* spp.) and microbe-feeding species (*Drosophila melanogaster* and *Scaptomyza* spp.) to investigate how plant-derived electrophilic toxins such as isothiocyanates (ITCs) affect insects, and how flies detect these compounds through olfaction. In survival assays, *D. melanogaster* exposed to volatile allyl isothiocyanate (AITC), a toxin derived from many Brassicales plants, were acutely intoxicated, demonstrating the high toxicity of this volatile compound to non-specialized insects. Through single sensillum recordings (SSR) from olfactory organs and behavioral assays, we found that the Odorant receptor 42a (Or42a) is necessary for AITC detection and behavioral aversion. Comparative transcriptome and RNA FISH studies across the drosophilid genus *Scaptomyza* revealed lineage-specific triplication of *Or42a* in the Brassicales specialists and a doubling of *Or42a*-positive-olfactory sensory neurons. Heterologous expression experiments showed that Or42a paralogs in Brassicales-specialists exhibited broadened sensitivity to ITCs in a paralog-specific manner. Finally, AlphaFold2 modeling followed by site-directed mutagenesis and SSR identified two critical amino acid substitutions that conferred *Or42a* heighten sensitivity to Brassicales-derived ITCs. Our findings suggest that ITCs, which are toxic to most insects, can be detected and avoided by non-specialists like *D. melanogaster* through olfaction. In Brassicales specialists, these same Ors experienced gene duplication events that resulted in an expanded sensitivity to ITC compounds. Thus, the insect’s olfactory system can rapidly adapt to toxic ecological niches provided by chemically-defended host plants through co-option of chemosensory capabilities already present in their ancestors.

## INTRODUCTION:

Plants have evolved the ability to synthesize a diverse array of potentially toxic specialized metabolites that provide resistance against insect herbivory. In turn, herbivorous insects have evolved diverse morphological, physiological, and behavioral counter-strategies to avoid these chemicals if encountered, or to mitigate their effects if ingested (Mithöfer and Boland 2012). Some herbivores even co-opt these plant toxins as oviposition or feeding stimulants and chemical defenses of their own. For example, monarch butterfly (*Danaus plexippus*) larvae evolved target site insensitivity in the sodium potassium ATPase against the cardenolides released from milkweed (*Asclepias* spp.) host plants upon wounding (Dobler et al. 2012). The caterpillars sequester these heart poisons, which serve as effective defenses against predation throughout the life of the insect (Reichstein et al. 1968). Although cardenolides have one major molecular target in insects, some plant toxins are far more promiscuous in their modes of action, which presents a different “evolutionary hurdle” ((Southwood 1972) to herbivores.

For example, to defend themselves against herbivores, Brassicales plants that include mustards (Brassicaeae) have evolved a sophisticated chemical defense system that produces promiscuous electrophilic toxins (Ahuja et al. 2010). These plants produce non-toxic glucosinolates from various amino acid precursors that are hydrolyzed *in planta* to form toxic compounds that include electrophilic isothiocyanates (ITCs) (Hopkins et al. 2009). ITCs are defined by a R−N=C=S functional group wherein the carbon is attacked by nucleophiles such as the sulfhydryl groups of cysteines. For example, allyl isothiocyanate (AITC) is derived from radish [*Raphanus sativus* (Cuellar-Nuñez et al. 2022)], while butyl isothiocyanate (BITC) and other ITCs bearing similar structures are derived from cabbage [*Brassica oleracea* (MacLeod et al. 1989)]. The chemical diversity of glucosinolates allows Brassicales plants to effectively deter a wide array of herbivorous insects because different species have different mixtures of glucosinolates, making it more difficult for insects to adapt (Winde and Wittstock 2011).

One herbivorous insect lineage that has evolved to cope with more promiscuous toxic plant metabolites like ITCs are leaf-mining drosophilid flies in the genus *Scaptomyza* (e.g., *S. flava*) which are specialized primarily on Brassicales plants. *Scaptomyza* species form a clade that is phylogenetically nested within the paraphyletic *Drosophila* subgenus. Gloss et al. (2014) found that yeast-feeding *Drosophila melanogaster* (*D. melanogaster*) uses promiscuous glutathione *S*-transferase (GST) in the gut to detoxify dietary ITCs released from glucosinolate-bearing Brassicales plants like thale cress (*Arabidopsis thaliana*), broccoli (*Brassica oleracea*) and wasabi (*Eutrema japonicum*). Further, they characterized a set of *Scaptomyza*-specific GSTs which experienced rapid gene duplication and non-synonymous changes during the evolution of herbivory that resulted in among the most efficient GSTs known from animals in detoxifying ITCs (Gloss et al. 2014; Gloss et al. 2019). Additionally, Brassicales-feeding *Scaptomyza* flies may minimize their exposure to glucosinolate-derived toxins by selectively ovipositing on leaves that contain lower glucosinolate concentrations (Humphrey et al. 2016).

Insects can behaviorally avoid toxic chemicals through gustation and olfaction (e.g. (Bernays and Chapman 1987; Stensmyr et al. 2012; Scott 2018; Chen and Dahanukar 2020; Dweck and Carlson 2020). In *D. melanogaster*, physical contact of ITCs by the tarsi and proboscis induces behavioral aversion through gustatory receptor cells in those organs that express the nociceptive “wasabi receptor” TrpA1 and Painless (Al-Anzi et al. 2006; Kang et al. 2010; Kim et al. 2010; Mandel et al. 2018). Volatile exposure to extracts containing phenethyl isothiocyanate (PITC) from turnip or rutabaga kills *D. melanogaster*, suggesting that volatile PITC is toxic to these insects, at biologically meaningful concentrations (Lichtenstein et al. 1964). Coupled with more recent findings of how volatile allyl isothiocyanate (AITC) causes aversion in fire ants (*Solenopsis invicta*) (Hashimoto et al. 2019), this intriguing finding raises the possibility that insects have evolved olfactory sensory neurons (OSNs) that detect plant-derived electrophilic compounds such as ITCs, leading to olfactory-mediated behavioral avoidance in generalists, as has been found in the gustatory system.

*Scaptomyza* includes species that are both non-herbivorous (e.g., microbe-feeding) and herbivorous species (Aguilar et al. 2024). However, the most recent common ancestor of the herbivores is hypothesized to have been microbe-feeding based on ancestral state reconstruction (Whiteman et al. 2011; Whiteman et al. 2012; Peláez et al. 2023). Of particular interest are the Brassicales plant specialists *S. flava* and *S. montana*, which are closely related species in the exclusively herbivorous subgenus *Scaptomyza* of the genus *Scaptomyza* (Kim et al. 2021). Additionally, *Or85d, Or42b, Or9a* and *Or22a,* which in *D. melanogaster* mediate the olfactory detection of fermentation products of brewer’s yeast and other microbes, have been lost in a stepwise fashion during the evolution of the herbivory in *Scaptomyza* (Goldman-Huertas et al. 2015). Furthermore, the *S. flava* paralogs of the antennal odorant receptor Or67b, which in *D. melanogaster* is present as a single copy and responds to green leaf volatiles such as *trans*-3-hexenol, respond in *S. flava* to a subset of ITCs (Matsunaga et al. 2021). Although there has been some progress, whether and to what extent generalist insects avoid volatile electrophilic compounds like ITCs on the one hand, and how evolution has reshaped the sensory receptors of specialists to facilitate both aversion and attraction on the other, is still largely unknown, yet is a central problem in understanding how organisms invade toxic niches.

We addressed this question in *D. melanogaster* and a set of *Scaptomyza* spp. exhibiting a gradient in microbe-feeding and plant-feeding habits. We first recapitulated the experiments of Lichtenstein et al. (1964), but instead of PITC, we used the more widely used AITC and found that *D. melanogaster* is rapidly killed by exposure to moderate concentrations of volatile AITC, demonstrating the insecticide potency of this electrophilic compound. This indicated that *D. melanogaster* may have evolved odorant receptors that respond to and release aversive behaviors toward volatile ITCs. We found that Or42a, which is expressed in maxillary palp-specific pb1a OSNs, is highly sensitive to AITC and is necessary for behavioral aversion to this compound in *D. melanogaster*. Furthermore, we discovered that mustard plant specialist *Scaptomyza* fly species *S. flava* and *S. montana*, but not microbe-feeding *Scaptomyza* species, have expanded their ITC sensitivity range. This was coupled with a concomitant increase in the number of *Or42a*-positive-OSNs in *S. flava* and *S. montana*, and with a triplication of the *Or42a* gene in *S. flava*. We then used site directed mutagenesis and found that via just two amino acid substitutions, one of these Or42a paralogs in *S. flava* switched its sensitivity from odors that activate the Or42a of microbe feeders to odors that activate mustard specialists, paralleling the niche shift of the mustard-feeding *Scaptomyza*. Taken together, these results illustrate how plant-derived volatile toxins like electrophilic ITCs can negatively impact insect behavior and fitness, how the insect olfactory system detects them leading to behavioral avoidance, and how host-plant specialization reshapes the organization and molecular function of toxin-detecting Ors.

## MATERIAL AND METHODS:

### Fly Husbandry

Microbe-feeding *D. melanogaster* was reared on cornmeal medium. Microbe-feeding *S. pallida* (subgenus *Parascaptomyza*) and *S. hsui* (subgenus *Hemiscaptomyza*) were reared in vials of cornmeal molasses media covered with a mixture of Carolina biological supply instant *Drosophila* media (Burlington, NC) mixed with blended spinach leaves, and then covered with a layer of defrosted frozen spinach leaves. The obligate leaf-miners *S. flava* and *S. montana* (subgenus *Scaptomyza*) were cultivated on potted fresh laboratory-grown *Arabidopsis thaliana* Col-0. Isofemale lines of microbe feeding *S. hsui* and *S. pallida*, as well as the herbivorous *S. montana,* were collected along Strawberry Creek on the UC Berkeley Campus in Berkeley, California, USA (Kim et al. 2021), and a line of *S. flava* was collected in from a meadow near Dover, New Hampshire, USA. All species were kept at 23±2°C and 60% relative humidity in a 10L:14D light cycle under fluorescent ballast lights. The following lines (stock #) were obtained from the Bloomington *Drosophila* Stock Center: *Orco* (23130), *Or42a*−/− (60821), *Or42a-Gal4* (9970), *w*^*1118*^ (3605), *TrpA1*^*1*^ (26504), and a genetic background control for the *Or42a*^*−*^ line (68384). The *Or67d*^*Gal4*^ line was a gift from the laboratory of Barry J. Dickson.

### Single sensillum recordings (SSRs)

One to five day-old fed female flies were prepared for Single Sensillum Recording (SSR) as described in (Matsunaga et al. 2021). Briefly, a silicon tube delivering a constant flow of charcoal-filtered air (16 ml/min, measured using a flowmeter, Gilmont Instruments, USA) was placed near the fly’s head capsule, and the tip of the stimulation pipette (50 ml) was inserted into the constant air stream. The stimulation pipette contained a 0.5 cm x 5 cm piece of filter paper loaded with 20 μl of an odorant solution or the solvent control. A pulse of clean air (duration = 1 sec) was delivered to the stimulus pipette using a membrane pump operated by a Stimulus Controller CS 55 (Syntech, Buchenbach, Germany). Sensilla identification was conducted by using the following diagnostic odorants (as described in (Dweck et al. 2016; Gonzalez et al. 2016); all >95% pure, Sigma-Aldrich, St. Louis, MO, USA): ethyl acetate (CAS # 141–78-6) for identifying *D. melanogaster* (*Dmel*) pb1a; AITC (CAS # 57–06-7) for identifying *Scaptomyza* pb1a-like olfactory sensory neurons (OSNs); 4-ethylguaiacol (CAS# 2785–89-9) for identifying *Dmel* and *Scaptomyza* pb1b-like OSNs; fenchone (CAS# 1195–79-5) for identifying *Dmel* and *Scaptomyza* pb2a OSNs; guaiacol (CAS# 90–05-1) for identifying *Dmel* and *Scaptomyza* pb2b OSNs; phenethyl acetate (CAS# 103–45-7) for identifying *Dmel* pb3b, *Sfla* pb3b, and *Smon* pb3a-like OSNs; and 2-heptanone for identifying *Shsu* and *Spal* pb3a-like OSNs. All odorants were diluted in mineral oil (CAS 8042–47-5) exceptγ-hexalactone (CAS # 695–06-7), which was diluted in dimethyl sulfoxide (DMSO: CAS# 67–68-5). Odorants were diluted to 1/100 vol/vol for stimulation unless otherwise noted. Supplementary file 1 lists all the chemicals used in this study.

The “net number of spikes/second” was obtained by counting the number of spikes originating from the OSN of interest within a 0.5-second timeframe which started 0.2 seconds after the onset of stimulation. This count was then adjusted by subtracting the background spiking activity (# of spikes within a 0.5 second interval preceding the stimulation), and then doubled to represent the number of spikes/second. In all figures, unless otherwise stated, we represent the “control-subtracted net # of spikes/sec” to odorant stimulation, calculated by subtracting the average net # of spikes/sec in response to the solvent control (mineral oil or DMSO) from the net # of spikes/sec evoked by each odorant stimulation. The BITC to γ-hexalactone spike ratio (Figure 4F and Figure supplement 10) was calculated as: [net # of spikes/sec evoked by BITC/ (net # of spikes/sec evoked by BITC + net # of spikes/sec evoked by γ-hexalactone)]. We used this denominator for the ratio because the control-subtracted net # of spikes by γ-hexalactone occasionally produced negative values (likely a response to the solvent control).

The *Or67d*^GAL4^ line was used to generate flies expressing *Or42a* homologs in the at1 “empty neuron” system, as described in (Kurtovic et al. 2007).

### Survival assay

To investigate the survival of wild-type *D. melanogaster* (Canton-S strain) flies in presence of AITC volatiles, we used a 9 cm diameter plastic petri dish (Nunc, Denmark) with a piece of fabric mesh placed horizontally between the base and the lid, which created two chambers (Figure supplement 1A). The upper chamber housed 8–10 three to five days old male flies, and the lower chamber contained four 5 μl drops of the odor solution (or the solvent control) evenly dispersed. Because the mesh prevented the flies from reaching the bottom chamber, insects were exposed to the volatile chemicals but could not directly contact (i.e. taste) the odor solution unless the molecules adhered to the walls of the chamber after volatilizing. After each test started, we counted the number of flies alive every 10 minutes up to one hour and calculated the percentage of survival. Flies exhibiting no movement for >30 seconds were considered dead. AITC or γ-hexalactone were diluted in either mineral oil or DMSO at various concentrations. Survival data analysis was performed using the log-rank (Mantel-Cox) test (Mantel 1966).

### Consumption assay

Groups of 2–4 days old mated female flies (n=11–15) were wet-starved for 24 hours, and then transferred to a vial containing a piece of filter paper (2.7 cm diameter, Whatman, cat. No 1001 125) impregnated with 160 μl of 50 mM D-glucose (Sigma-Aldrich, USA) dyed blue with Erioglaucine (0.25 mg/ml, Sigma-Aldrich, St. Louis, MO, USA) (Reisenman and Scott 2019) (Figure supplement 1B). Flies were allowed to feed for 15 minutes (10 minutes in tests with γ-hexalactone), vials were frozen (>60 minutes), and the amount of blue dye in the flies’ abdomen was scored blind to treatment (see below). The odor source consisted of a strip of filter paper (0.25 cm wide x 1.5 cm long) impregnated with either 10 μl of an odorant solution (test) or 10 μl of the solvent (control), which was placed inside a container (1.3 cm long x 0.75 cm diameter) with a meshed bottom affixed to the vial’s flug (Figure supplement 1B). This allowed diffusion of odors into the fly vial but prevented flies from contacting the odor source. Control tests, with vials containing food solution but only the solvent control inside the meshed container, were conducted in parallel with experimental tests to control for fly cohort and day-to-day variability.

Food consumption was estimated by scoring individual flies in each vial blind to treatment using the following five-point scale (Reisenman and Scott 2019): 0 (no dye = no food), 0.25 (“trace” of blue dye), 0.5 (up to ¼ of the abdomen dyed blue), 1 (more than ¼ but less than ½ of the abdomen dyed blue), and 2 (more than ½ of the abdomen dyed blue). For each vial, a single feeding score value was calculated as: [(0 x n_0_ + 0.25 x n_0.25_ + 0.5 x n_0.5_ + 1 x n_1_ + 2 x n_2_) / N], where n_(0–2)_ denotes the number of flies in each score category, and N the total number of flies/vial. Each odor vial datapoint was normalized by dividing the feeding score in presence of odor by the averaged feeding score for the corresponding day and genotype in absence of odor. Normalized data for each genotype and odor was compared against the expected median=1 using one sample signed rank tests. That is, medians significantly less than 1 or more than 1 respectively indicate feeding aversion and enhancement; medians not different from 1 indicate that the odorant did not reduce neither enhanced consumption. Normalized data from control and mutant flies were compared using Mann-Whitney U tests. In all cases results were considered statistically significant if p<0.05. The consumption assay data is compiled in Supplementary file 4.

### Positional olfactory assay

To study the olfactory orientation of insects towards odors, we conducted group assays with fed 3–4 days old, mated females (Figure supplement 1C). Flies (n=10–12/test) were briefly anesthetized on ice and placed in a small piece of clear Tygon tube, capped in both sides with the bottom of a conical PCR plastic Eppendorf. After another about 3–4 minutes, the Tygon tube with the anesthetized flies was uncapped and connected to the cut ends of two glass Pasteur pipettes and the assay started; flies usually resume activity after about three-four minutes. Each of the two opposite ends of the pipettes were respectively connected (via a small piece of Tygon tube) to a glass vial (capacity: 0.5 ml) containing 10 μl of the odor solution (AITC 1: 500 vol/vol, or γ-hexalactone 1:100 or 1:10 vol/vol) or 10 μl of the control solvent (mineral oil, or DMSO). Tests with apple cider vinegar used 30 μl instead, and water was used as a control. The distal ends of the pipettes were separated from the glass vials with a small piece of fabric mesh, which allowed the odorant to diffuse into the pipette while also preventing insects from contacting the odor source (Figure supplement 1C). The odor and control sides were switched between assays. Assays were conducted on a white surface under white light at 22–24 °C, about 2–6 hours after lights on. Once each assay started, the number of flies in the pipette closest to the vial with the odor (referred to as “odor side” or “odorous tube” for simplicity), in the pipette closest to the vial with the solvent control (“control” side/tube), and in the Tygon tube that connected both pipettes (release site) were counted every five minutes until minute 35, and then again at 65 minutes in the case of tests with AITC. For each assay, we calculated the % of insects that made a choice for one or the other tube as: [(#of insects in the odor side + # of insects in the control side) / total number of insects released)] x 100. The percentage of insects that choose the odorous tube was calculated based on the total number of insects that made a choice as: [# of insects in the odor side / (# of insects in the odor side + # of insects in the control side)] x 100. Assays in which less than 40% of insects made a choice for either side at all time points were discarded (<5% of assays). For each fly genotype and odorant, the % of insects that choose the odor side at each time point was compared against the median value expected under the hypothesis that insects distributed at random between the two tubes (50% of insects in each tube) using one-sample signed rank tests. Thus, the insects either avoid the odorous tube (if median<50%) or prefer it (if median>50%). Also, at each time point and for each odor (and concentration when applicable), the responses of *Or42a*−/− and its genetic background control (Bloomington Stock Center # 68384) were compared via Mann-Whitney U tests. In all cases results were considered significant if p<0.05. The positional olfactory assay data is included in Supplementary file 5.

### RNA-sequencing of maxillary palps

Newly emerged adults of *S. flava* and *S. pallida* were collected from our colony and kept in humidified vials with 10% honey water until dissection, to minimize potential differences in nutrition resulting from differences in the two species’ larval diet. Three to ten days old flies were anesthetized with CO_2_, and their maxillary palps were hand-dissected using forceps. Approximately 100–120 flies were pooled for a single sample. The dissected tissues were directly collected in LB+TGA lysis buffer from Reliaprep RNA Tissue Miniprep System (Promega, USA), and homogenized using a Biomasher Standard homogenizer (Takara Bio Inc., USA) in a dry ice ethanol bath. The sample lysates were stored at −80°C until RNA extraction. Total RNAs were extracted from the lysates using ReliaPrep RNA Tissue Miniprep System (Promega, USA) according to the manufacturer’s protocol, and quantified using a Qubit RNA High Sensitivity kit (Thermo Fisher Scientific, USA). Library preparation was performed at the Functional Genomics Laboratory (FGL) at UC Berkeley. Due to the low yields of our maxillary palp-derived total RNAs, cDNA libraries were first produced by Takara SMART-Seq mRNA Ultra-low input RNA kit (Takara Bio Inc., USA) with 8 cycles of PCR for the amplification, and then processed by KAPA HyperPrep kit for DNA (Roche Sequencing, USA) with 9 cycles of PCR for attaching in-house sequencing adapters and index primers. cDNA libraries were then transferred to the Vincent J. Coates Genomics Sequencing Laboratory (GSL) at UC Berkeley and sequenced on an Illumina NovaSeq 6000 150 PE S4 flowcell, targeting 25M read pairs per library. For read mapping, we used previously reported reference genome assemblies and gene annotations from *S. flava* (Peláez et al. 2023) and *S. pallida* (Kim et al. 2021) for subsequent bioinformatic analyses. Raw RNA-seq reads were filtered using Fastp v0.21.0 (Chen et al. 2018) and mapped to the respective reference genomes using STAR v2.7.1a (Dobin et al. 2013) to generate multiple alignment (BAM) files, which were then converted to read count data using HTseq v0.9.1 (Anders et al. 2015). Count data for the Or gene family were converted to RPM (reads per million); data is compiled in Supplementary file 6.

### HCR RNA FISH

One-to-four-day-old female *S. flava* and *S. pallida* were collected and anesthetized with CO_2_. Whole mouthparts were removed proximal to the attachment of the maxillary palps with Inox 5 forceps (Fine Science Tools, Foster City, CA) on a Sylgard-covered petri dish. Mouthparts from each species were immediately placed in 2 mL of fixative (4% vol/vol paraformaldehyde in 1x phosphate buffered saline with 3% vol/vol Triton X-100 added, PBST) in a LoBind Eppendorf tube, and fixed for 22 hours at 4°C on a nutator.

Fixative was carefully pipetted out from samples and replaced with 2 mL of cold (4°C) 3% PBST, and tubes were agitated on a nutator for 5 minutes. This step was repeated once more and then again with cold 0.1% PBST; samples were then washed with 1 mL of cold 0.1% PBST 4 times and agitated on a nutator (5 min for each wash). PBST was removed and replaced with 300 μL of warm (37°C) HCR RNA FISH probe hybridization buffer (Molecular Instruments, Inc., Los Angeles, CA) and incubated for 30 minutes. The probe hybridization solution was prepared by adding 5μL of 1μM of probe B1-*Spal Or42a* and probe B2-*Spal Orco* (or probe B1-*Sfla Or42a* and probe B2-*Sfla Orco*) to 300 μL of pre-heated probe hybridization buffer for the *S. pallida* or the *S. flava* tissues and kept at 37°C. Finally, the buffer was replaced with the preincubated probes in hybridization buffer and kept at 37°C for 22 hours.

Samples were next washed 3 times with 500 μL of preheated (37°C) probe wash buffer for 5 min on a heating block. Ten μL of 3μM amplification hairpins one and two of b1–488 and b2–637 HCR amplifiers (Molecular Instruments, Inc., Los Angeles, CA) were snap cooled on a thermocycler at 95°C for 90 secs and then at 20°C for 30 min, and then covered with aluminum foil. Samples were washed 3 times with 500 μL of 0.75M NaCl, 75mM sodium citrate and 0.1% Tween-20 vol/vol (SSCT) at room temperature for 5 minutes, and then incubated in 500 μL amplification buffer (Molecular Instruments, Inc., Los Angeles, CA) for 30 minutes at room temperature. Snap cooled hairpins were added to two vials containing 300 μL of amplification buffer for each species. The amplification buffer was removed from the samples and replaced with the hairpin solution; tubes were covered in aluminum foil and incubated at room temperature for 22 hours.

Samples were washed twice with 500 μL of SSCT solution for 5 minutes, twice for 30 minutes, and finally washed again for 5 minutes (vials were kept on a nutator in between washes). Samples were then stained with 300 nM DAPI stain in 0.1% PBST for 15 minutes, and then washed 3 times with 0.1% PBST for 5 minutes. Tissues were transferred to a microscope slide and mounted in a drop of ProLong diamond antifade mounting (Life Technologies Corp., Eugene, OR) and stored at 4°C until examination. Confocal imaging of fixed samples was performed using a Zeiss LSM 880 microscope in the AiryScan mode. Raw images were processed using Zeiss ZEN Black software. *Orco*-positive cells (visualized with the 488 nm laser) and *Or42a*-positive cells (visualized with the 633 nm laser) were manually counted using the “Cell Counter” plugin in Fiji (ImageJ) software. Supplementary file 7 contains cell counts of RNA FISH.

### *Scaptomyza Or42a* gene cloning and generation of *UAS* lines

RNA was isolated from 15–25 days laboratory-reared adults of both sexes of *S. pallida* and *S. flava* using the ReliaPrep Miniprep system (Promega, W.I., USA). Complementary DNA (cDNA) was synthesized using the qScript cDNA Supermix (Quantabio, Beverly, MA, USA). The paralogs were amplified via touchdown PCR, using primers that target the highly variable regions of the 5’ and 3’ untranslated regions (UTRs). The single *Or42a* gene of *S. pallida* was amplified using touchdown PCR with primers targeting the coding region (Q5 DNA Polymerase, #M0491, NEB) (Supplementary file 8). All PCR products underwent gel purification (11–300C, Zymo Research) and were subsequently cloned using the Gibson Assembly (E2611S, NEB) following the manufacturer’s instructions into the UAST-attB vector [DGRC Stock 1419; RRID:DGRC1419, (Bischof et al. 2007)]. The ligation products were transformed into DH5α competent cells (T3007, Zymo Research). After confirming the sequences, the 5’ and 3’ UTRs of the plasmids containing the *S. flava Or42a* paralogs were removed. This was achieved by first amplifying the coding region using PCR, followed by gel purification and ligation back into UAST-attB vector via Gibson Assembly. The resultant plasmids were sequence-verified. Finally, these plasmids were microinjected into the attP40 site (P{nos-phiC31\int.NLS}X, P{CaryP}attP40, line # 25709, Rainbowgene) to create transgenic lines for *Spal Or42a*, *Sfla Or42a2*, *Sfla Or42a3* and *Sfla Or42a4*. All lines were confirmed by sequencing prior to experiments.

### Screen of candidate amino acids using branch-site test and Colabfold 3D prediction

CDS of *Sfla Or42a3* and *Sfla Or42a4* were confirmed by palp RNAseq data using IGV_2.16.0. CDSs of *Sfla Or42a3* and *Sfla Or42a4* were then used as inputs into ColabFold (Jumper et al. 2021; Mirdita et al. 2022). Then the output models ranked first (rank1) were selected, visualized, and 3D-aligned by using PyMol2.5.3. Since previous studies suggested that the binding pockets of odorant receptors are located in the transmembrane region (Butterwick et al. 2018; Del Mármol et al. 2021; Zhao et al. 2024), in silico substitutions of amino acids were individually conducted on *Sfla Or42a3*. The resulting amino acid sequences were then re-inputted into ColabFold, the model with rank1 was selected, and the sequences were 3D-aligned with *Sfla* Or42a4. This process was repeated until the 3D structures of transmembrane regions between the chimera and *Sfla* Or42a4 matched in PyMol space. All pdb files used in this study are included in Supplementary file 9.

### Site-directed mutagenesis

Conventional PCR was conducted using plasmids of *Sfla Or42a3* as backbone (Q5 DNA polymerase, #M0491, NEB; Supplemental File 1). Primers were designed to introduce the point mutations (Supplementary File 1). The PCR product underwent gel purification (11–300C, Zymo Research) and the methylated plasmids were digested with DpnI for 1 hour at 37°C (QuickChange, Agilient Technology, USA). Ligation was performed by incubation at 16°C for 30 minutes (DNA ligation kit Mighty mix, Takara, Japan) and the products were transformed into DH5α competent cells (Takara, Japan). After confirming the sequence, the plasmid was microinjected into the attP40 site (P{nos-phiC31\int.NLS}X, P{CaryP}attP40, line # 25709, Rainbowgene) to create transgenic A181D S307P fly. The line was confirmed by sequencing prior to experiments.

## RESULTS

### Volatile AITC rapidly kills *D. melanogaster*.

**Figure 1:**
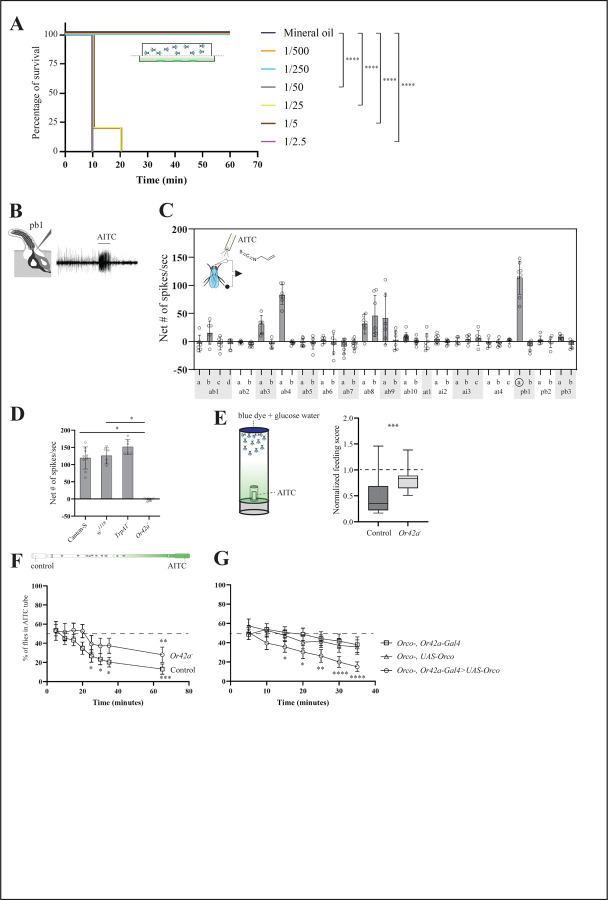
*Drosophila melanogaster* Or42a mediates volatile toxicity of allyl isothiocyanate (AITC) and behavioral aversion to this compound **(A)** Survival of *D. melanogaster* flies exposed to various concentrations of volatile AITC (no direct contact with the source of the AITC was allowed). The experimental arena (Figure Supplement 1A) consisted of two chambers, one housing a group of 8–10 flies and the other containing four 5 μL of AITC. All flies survived exposure to the solvent control and AITC 1:250 or 1:500 vol/vol. Flies exposed to higher concentrations of AITC survived less than 20 minutes, while those exposed to 1:5 or 1:2.5 vol/vol survived less than 10 minutes (**** p<0.0001, log-rank Mantel-Cox tests against the control). **(B)** Schematic and representative single sensillum recording (SSR) from pb1 OSNs upon stimulation with AITC 1:100 vol/vol. **(C)** SSR from all *D. melanogaster* antennae (ab) and palp (pb) basiconic sensilla to AITC stimulation (1:100 vol/vol). Here and thereafter, bar graph bars represent the control-subtracted net number of spikes (mean ± SEM) unless otherwise noted, and symbols indicate individual data points. AITC activates pb1a neurons the most, followed by ab4a neurons (n=6–10 recordings/sensilla type from 6 animals). **(D)** Responses from pb1 sensilla of Canton-S, *w*^*1118*^, *TrpA1*^*−*^, and *Or42a−/− D. melanogaster* flies to 1:100 vol/vol AITC stimulation. Flies from all genotypes except *Or42a-*responded to AITC, which indicates that *OR42a* is required for olfactory detection of this odor in pb1a (n=6–9 sensilla/genotype from 3–4 animals/genotype). Responses from all genotypes were different from those of *Or42a* mutants (Kruskal-Wallis ANOVA followed by Dunn’s multiple comparisons, *p<0.05). **(E)** Food consumption of mutants and background genotype control flies in presence or absence of a non-toxic concentration of volatile AITC (1:500 vol/vol). Data represents the fly’s normalized feeding scores (horizontal bars: median, edges of boxes: 25 and 25% quartiles, whiskers: 10 and 90% quartiles) in presence of AITC volatiles; the dashed line at 1 indicates neither aversion nor feeding enhancement. AITC volatiles strongly reduced feeding in control flies (median=0.35; one-sample signed rank test against median=1, p<0.001, n=16). AITC volatiles slightly reduced feeding in *Or42a* mutants (median=0.83, p<0.05, n=14), but their normalized feeding score was statistically different from that of control flies (Mann-Whitney U test, ***p<0.001, n=16, 14). **(F)** Positional olfactory assay. Groups of 10–12 non-starved females were released in the middle of a dispositive consisting of two glass tubes, each connected to a glass vial containing 10 μl of AITC 1:500 vol/vol or 10 μL of the solvent control (Figure supplement 1B). The number of flies in each of the glass tubes were counted every five 5 minutes until minute 35, and then again at 65 minutes. Data show the % of insects (mean ± SEM) in the tube closest to the odorant source at each timepoint (n=15/genotype). The dotted line at 50% indicates random distribution between the two tubes. Control insects avoided the tube closest to the odor source at 25, 30, 35 and 65 minutes (one-sample signed rank tests against median=50%, ***p<0.005 in all cases; p=0.068 at 20 minutes). *Or42a*−/− mutants distributed randomly between the two tubes at all timepoints (at 30 and 35 minutes p=0.055 and 0.078, respectively) except at 65 minutes (**p<0.01). **(G)** Positional olfactory assay using *Orco*−/− flies in which orco olfactory function was specifically rescued in the OR42a circuit. Tests were conducted as in **F** using AITC 1:500 vol/vol. The two parental control lines (*UAS-Orco* and *Or42a-Gal4*) distributed at random between the two tubes, while rescued flies (*Or42a-Gal4*>*UAS-Orco*) significantly avoided the tube closest to the odor source from minute 15 onwards (*p<0.05; **p<0.01; ****p<0.001, n=15, 16, 13).

We conducted survival assays with various concentrations of volatile AITC (from 1/500 to 1/2.5 vol/vol). In our experimental setup, a fabric mesh separated the chamber containing the flies from the chamber containing one of the ITC solutions (Figure supplement 1A) and therefore, flies were exposed to volatile AITC but could not contact (i.e. taste) the AITC solution directly. While all animals in both the control treatment and those exposed to AITC 1:250 and 1:500 vol/vol survived, most flies exposed to AITC concentrations ≥ 1/50 vol/vol became paralyzed (highly intoxicated or dead) within 10 minutes (Figure 1A, log-rank Mantel-Cox tests, p<0.0001). Thus, this rapid immobilization indicates that plant-derived electrophilic compounds like AITC can have a strong negative effect on fly survival, consistent with previous findings using volatile PITC (Lichtenstein et al. 1964).

#### Volatile AITC is detected by Or42a in *D. melanogaster*

The high lethality caused by volatile AITC indicates that this volatile had the potential to be detected by activating the fly’s olfactory system, and that this could provide a fitness benefit if these volatile toxins were then behaviorally avoided. To investigate this, we conducted exhaustive single sensillum recordings (SSR) from the fly’s olfactory organs, the antennae and maxillary palps, upon stimulation with volatile AITC. Several OSNs showed excitatory responses to AITC, but OSNs in palp basiconic (pb) sensilla (pb1a) were the most strongly activated (Figure 1 B-C, 113.8 ± 10.4 spikes/sec from pb1a, n=8). Because previous studies reported that *D. melanogaster* pb1a OSNs express *Or42a* (Couto et al. 2005), we investigated whether AITC responses in pb1a OSNs are indeed mediated by Or42a. OSNs in pb1 sensilla from control flies showed strong responses to volatile AITC (Figure 1D, Canton-S flies: 119.3 ± 10.8 spikes/sec, n=9; *w*^*1118*^ flies: 126.7 ± 9.8 spikes/sec, n=6). In contrast, ONSs in pb1 sensilla from *Or42a* null mutant flies showed no response (−1.6 ±1.74 spikes/sec, n=6; Kruskal-Wallis test with Dunn’s multiple comparison, p<0.05 in both cases), suggesting that Or42a detects volatile AITC in these sensilla. Furthermore, OSNs in pb1a of *TrpA1* null mutant flies still responded to AITC, suggesting that the contact chemoreceptor TrpA1 is not necessary for maxillary palp olfactory detection of this compound (Figure 1D, 152.7 ± 9.1 spikes/sec, n=6; p>0.05).

#### Volatile AITC repels *D. melanogaster* via Or42a

Next, we examined if activation of OSNs housing Or42a play a role at the behavioral level (whole organism). Previous studies have shown that the presence of food-related odorants increase sugar consumption in *D. melanogaster* (Reisenman and Scott, 2019). Based on this, we hypothesized that the presence of aversive odorants such as AITC would decrease sugar consumption. To investigate this hypothesis, we conducted consumption assays. In brief, groups of 10–15 food-deprived female flies were offered 50 mM glucose water solution dyed blue in the presence or absence of volatile AITC (1:500 vol/vol loaded on filter paper), such as that flies could smell, but not contact, the AITC solution (Figure supplement 1B). After 15 minutes flies were frozen and then the amount of blue dye in the abdomen of each fly within a vial was quantified blind to treatment; these values were used to calculate a single feeding score/vial (see materials and methods). We found that genetic background control flies and wild-type Canton-S flies fed less when volatile AITC was present than in presence of the solvent control (one-sample signed rank test on normalized data, p<0.001, n=16; Figure 1E). Although mutant flies still fed less in presence than in absence of AITC volatiles (p<0.05, n=14), the reduction in consumption was lower than that of the genetic control group (median normalized feeding scores: 0.35 and 0.83 in control and mutant flies, respectively; p<0.005, Mann-Whitney U test). These results indicate that Or42a mediates aversion to AITC in this behavioral context.

Additionally, we conducted a positional olfactory assay based on Ohashi and Sakai (2015), with various modifications to prevent flies from physically contacting the odor source (Figure supplement 1C). Groups of 10–12 female flies were briefly ice-anesthetized and released at the juncture between the two glass pipettes, allowing flies to choose between the pipette closest to the odor source (hereafter referred to as “odorous side/tube”, for simplicity) and the pipette closest to the solvent (control side/odorless tube; Figure supplement 1C). The number of flies in each tube as well as in the middle section where flies were released was counted every five minutes up to minute 35, and then again at 65 minutes in tests with AITC. We first confirmed that flies can show normal olfactory-guided behavior in this behavioral set-up using apple cider vinegar, a well-established olfactory attractant (Semmelhack and Wang 2009; Becher et al. 2010). Canton-S flies preferred the odorous tube from minute 20 onwards (one-sample signed rank tests, p<0.05 in all cases, p=0.09 at minute 15, n=21). We then tested whether and to what degree AITC causes olfactory repellence in this behavioral context. Genetic control flies and wild-type Canton-S flies avoided the AITC tube at various time points (25, 30, 35 and 65 minutes in genetic controls, one-sample signed rank tests, p<0.005 in all cases, n=15 Figure 1F; and from 25 minutes onwards in Canton-S, p<0.05 in all cases, n=19; ), while *Or42a*−/− mutants only avoided the AITC tube at the 65 minutes time point (p<0.005). These findings suggest that *Or42a* plays a crucial role in mediating olfactory-driven behavioral aversion to AITC. Similar percentages of genetic control and *Or42a*−/− mutants choose one or the other tube at all time points (the average of medians over all time points were 79.7% and 82.5%, respectively; Mann-Whitney U tests, p>0.05 at all-time points; Figure supplement 2C), suggesting that *Or42a*−/− mutants are as active as wild-type flies in presence of AITC. We also confirmed that *Or42a*−/− mutants are capable of odor-mediated olfactory orientation. We conducted dual-choice trap assays offering apple cider vinegar vs. water as described in Matsunaga et al. (2019). About 50% (median) of flies released in each test (n=8, n=20 females/test) were trapped and of these, 100% (median) were found in the odor-baited trap (Wilcoxon-matched pairs test, p<0.005, n=8). This result confirms that the lack of behavioral aversion to AITC in *Or42a*−/− was not due to generalized olfactory defect.

We also tested whether the *Or42a* olfactory circuit can *per se* mediate olfactory avoidance of volatile AITC (Figure 1G). We conducted positional assays with *Orco* null mutant flies in which we rescued the olfactory function specifically in the *Or42a* circuit. While the two parental control groups (UAS-Orco, n=15, and Or42a-Gal4, n=16) distributed at random between the odorous and the control tubes at all time points (one sample signed rank tests, p>0.05 in all cases), rescued flies (Or42a-Gal4> UAS-Orco, n=13) significantly avoided the AITC tube from 15 minutes onwards (p<0.05 at 15, 20 and 25 minutes, p<0.001 at 30 and 35 minutes; tests stopped at 35 minutes). These results support the hypothesis that Or42a OSNs in pb1a alone are sufficient to induce aversion to AITC.

It has been reported that Or42a mediates behavioral attraction towards fruit/fermentation volatiles, including γ-hexalactone (Dweck et al. 2016). Thus, we used this odorant to test whether our behavioral assays yielded results consistent with these previous findings. In consumption assays lasting 10 minutes, Canton-S flies increased their feeding in the presence of volatile γ-hexalactone 1:50 vol/vol (normalized median=1.65, n=25, one-sample rank test, p=0.001), but this effect was lost in *Or42a*−/− mutants (normalized median=0.89, n=24, p=0.5; Figure Supplement 2A). In the positional olfactory assay, a larger proportion of genetic control flies than of *Or42a*−/− mutants choose either tube in presence of 1:10 vol/vol γ-hexalactone (Figure Supplement 2D, range of medians for all time points: 91% and 50–89% in control and mutant flies, respectively; Mann-Whitney U tests, p<0.05 in all cases), suggesting that the presence of the odor increases exploratory activity in control flies. Moreover, control flies and wild-type Canton-S flies showed attraction to 1:10 vol/vol γ-hexalactone (at 20 and 30 minutes in genetic control, n=19, and at 5, 10, and 15 minutes in Canton-S, n=12; one-sample signed rank tests, p<0.05 in all cases), but this attraction was lost in *Or42a*−/− mutants (Figure supplement 1B). These behavioral attraction phenotypes are consistent with the results reported by Dweck et al. (2017), and with our findings showing that volatile γ-hexalactone did not negatively affect survival (Figure supplement 1E).

Given that we found that Or42a participates in both mediating repellence to AITC and attraction to γ-hexalactone, we next tested whether these two odorants activate additional OSNs. Through exhaustive SSR from antennae and maxillary palps upon stimulation with γ-hexalactone, we found that pb1a OSNs was the only olfactory sensillum activated by γ-hexalactone (Figure supplement 2F), while AITC activated several additional OSN types (e.g. ab4a; Figure 1C). In sum, our data suggested that activation of *Or42a*-positive-OSNs, along with other Or-expressing OSNs likely mediates behavioral aversion, whereas the simultaneous activation of *Or42a*-positive-OSNs and other yet uncharacterized olfactory circuits involving e.g. Ionotropic receptors (Benton et al. 2009) would mediate behavioral attraction.

#### Pb1a-like OSNs in Brassicales-specialists evolved broadened sensitivity to isothiocyanates (ITCs)

We next investigated how the evolutionary transitions from microbe-feeding to herbivory have affected OSNs, using the reference species *D. melanogaster* as well as species within *Scaptomyza*: the microbivorous *S. hsui* and *S. pallida,* and the herbivorous Brassicales specialists *S. flava* and *S. montana* (Kim et al. 2021; Peláez et al. 2023). We investigated whether *Scaptomyza* species have pb1-like sensilla homologous to *D. melanogaster* pb1 and if so, the extent to which they respond to a broader range of ITC compounds, since Brassicales plants release ITCs upon wounding (MacLeod et al. 1989; Cuellar-Nuñez et al. 2022).

**Figure 2:**
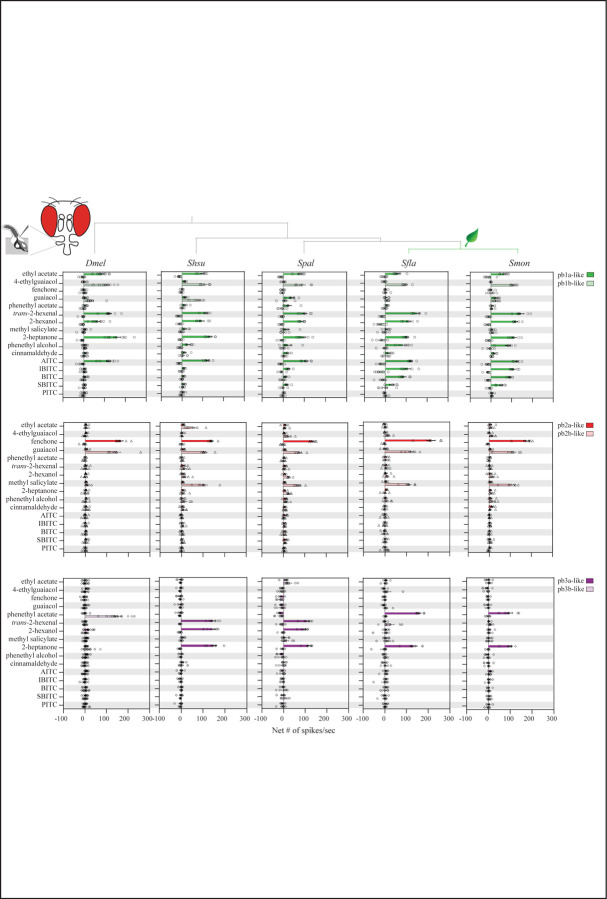
Functional characterization of maxillary palp OSNs in *D. melanogaster* and *Scaptomyza* species. Single sensillum recordings (SSRs) were conducted from maxillary palp OSNs in *D. melanogaster* (*Dmel*), *S. hsui* (*Shsu*), *S. pallida* (*Shsu*), *S. flava* (*Sfla*), and *S. montana* (*Smon*). Stimuli (all presented at 1:100 vol/vol) included diagnostic chemicals used to identify Ors in *D. melanogaster* such as ethyl acetate (to identify Or42a in *Dmel* pb1a), 4-ethylguaiacol (Or71a in *Dmel* pb1b), fenchone (Or85e in *Dmel* pb2a), guaiacol (Or46a in *Dmel* pb2b), and phenethyl acetate (Or85d in *Dmel* pb3b), along with fruit volatiles, green leaf volatiles (GLVs), and Brassicales plant-derived isothiocyanate (ITCs). Each panel depicts the control-subtracted net number of spikes/sec (average ± SEM, n=18–27 sensilla from 3–4 animals/species) in response to odor stimulation (duration=1 sec). All sensilla housed two OSNs, labeled “a” (darker color) and “b” (lighter color). Their response profiles and stereotyped locations within the palp support the classification of *Scaptomyza* sensilla in three types (Figure 3, Supplementary figure 5), named pb1-like, pb2-like, and pb3-like. In all *Scaptomyza* species we identified OSNs exhibiting an odorant tuning pattern similar to that of *Dmel* pb1a sensilla. Among these, one OSN type co-housed with pb1a-like OSNs exhibited a tuning pattern comparable to that of *Dmel* pb1b, while the other OSN co-housed with pb1a-like OSNs showed subthreshold activity and thus it was excluded from analysis. While pb1a sensilla from all species responded to AITC, pb1a-like from *S. flava* and *S. montana* additionally responded to other ITC compounds. Mustard specialization occurred at the clade leading to the common ancestor of these two species, denoted by the leaf cartoon. AITC: Allyl ITC, IBITC: Isobutyl ITC, BITC: Butyl ITC, SBITC: Sec-butyl ITC, PITC: Phenethyl ITC.

We first validated our methods for functional characterization of sensilla in *D. melanogaster*. We used compounds that serve as diagnostic for the three sensilla types found in the maxillary palps of *D. melanogaster* (see methods for details). In addition, we used as stimuli *trans*-2-hexenal, 2-hexanol, methyl salicylate, 2-heptanone and phenethyl alcohol, and Brassicales-derived isothiocyanates (ITCs) including allyl ITC (AITC), isobutyl ITC (IBITC), butyl ITC (BITC), sec-butyl ITC (SBITC), and phenethyl ITC (PITC). Using the diagnostic odorants, we confirmed the presence of three different types of palp sensilla (pb1, pb2 and pb3) in *D. melanogaster*. We also found that *Dmel* pb1a OSNs were activated by AITC (128.3 ± 10.8 spikes/sec, n=9), but not by the other ITCs tested (<10 spikes/sec, Figure 2).

We next characterized OSNs in pb sensilla in four *Scaptomyza* species and used the spike rate to determine if data clustered by sensilla type (Figure 2, Figure supplement 3A). In all species, the sensilla fell into three functional classes similar to *Dmel* pb1, pb2 and pb3, which we termed pb1-like, pb2-like, and pb3-like. The response profiles of *Scaptomyza* pb3-like OSNs were different from those of *Dmel* pb3, likely because the homologs of *Or59c* and *Or85d*, which are respectively expressed in *Dmel* pb3a and p3b, are unidentified in the genomes of *Scaptomyza* (Goldman-Huertas et al. 2015). We found two types of pb1-like sensilla in the four *Scaptomyza* species, each of which exhibited an odorant tuning pattern similar to that of *Dmel* pb1a. However, only one subset of *Scaptomyza* pb1b-like sensilla exhibited an odorant tuning pattern similar to that of *Dmel* pb1b, while the other subset showed subthreshold activity and thus was excluded from analysis. As in *Dmel*, AITC activated pb1a-like sensilla in all four *Scaptomyza* species (Figure 2). Notably, several other ITCs, including IBITC, BITC, and SBITC, additionally activated *S. flava* (*Sfla*) and *S. montana* (*Smon*) pb1a-like OSNs (the average # of spikes/second ranged from 30.3 to 107.3 across ITCs in *Sfla* pb1a-like, and 48.3 to 110.3 in *Smon* pb1a-like, n=6/ITC and species; Figure 2). Methyl salicylate activated all *Scaptomyza* pb2b-like sensilla but not *Dmel* pb2b (the average spike rates across *Scaptomyza* pb1a-like ranged from 70 to 110.7 spikes/second, n=6–7, compared to 11.6 ± 4.4 spikes/second for *Dmel* pb2b, n=9; Figure 2) and interestingly, this compound is a known *S. flava* volatile attractant in natural settings (Orre et al. 2010). Furthermore, the odorant response profiles of OSNs in pb3-like sensilla were more similar between the more distantly related species *S. hsui* and *S. pallida,* than between the more closely related species *S. pallida* and *S. flava,* or *S. pallida* and *S. montana.* For example, *trans*-2-hexenal activated *Shsu* and *Spal* OSNs in pb3a-like sensilla but not OSNs of *Sfla* or *Smon* in pb3a-like sensilla, while phenethyl acetate activated *Sfla* and *Smon* OSNs in pb3a-like but not those of *Shsu* or *Spal* pb3a-like sensilla (Figure supplement 3B-C). Thus, niche differences rather than phylogenetic distance may explain the functional divergence between OSNs in maxillary palp sensilla in these *Scaptomyza* species.

### Brassicales specialists have a higher number of pb1-like sensilla.

**Figure 3:**
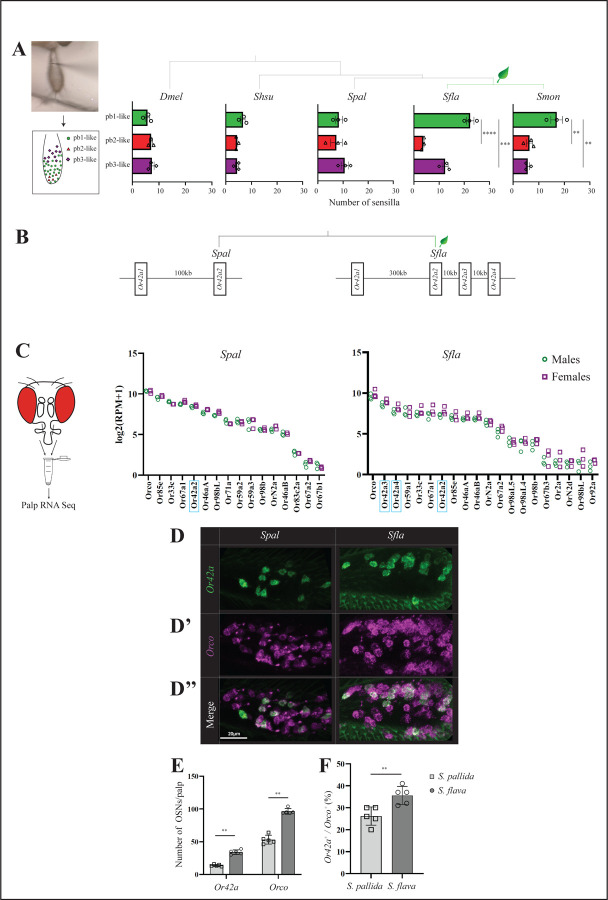
High expression of *Or42a* paralogs and over-representation of *Or42a*-positive-OSNs in the maxillary palp of mustard specialist flies. **(A)** Picture of SSR from *Sfla* maxillary palp sensilla (top left) and a representative anatomical mapping of *S. flava* palp sensilla (bottom left) obtained using diagnostic chemicals. Squares, triangles, and circles in the cartoon indicate the location of the three types of maxillary palp *Sfla* sensilla, and bars in the graph (right) represent the number of each sensilla type (green: pb1-like, red: pb2-like, purple: pb3-like) in *Dmel* and the four *Scaptomyza* species (see Figure supplement 4 for individual maps; n=3 animals/species, symbols). Asterisks indicate significant differences in the number of pb sensilla within a species (one-way ANOVA with Holm-Šídák’s multiple comparisons test, **p<0.0001). **(B)** Schematic of *Or42a* syntenic regions in the genomes of *Spal* and *Sfla*, with a gene triplication in the *Sfla* genome at the syntenic region of *Spal Or42a2* (*Sfla Or42a2*, *Sfla Or42a3*, and *Sfla Or42a4*). **(C)** Maxillary palp RNA seq of *Spal* and *Sfla Or*s (n=3 replicates/sex and species; symbols), indicating high expression of *Spal Or42a2*, *Sfla Or42a2*, *Sfla Or42a3*, *Sfla Or42a4*, and the absence of *Or42a1* from *S. pallida* and *S. flava*. *Or*s with the log2 (RPM +1) < 1 average values (n=3) were excluded. **(D-D”)** Representative images of HCR RNA FISH from the maxillary palps of *Spal* and *Sfla* showing *Or42a*-positive-OSNs (**D**, green), *Orco*-positive-OSNs (**D’**, magenta), and the merged signals (**D’’**, white indicates co-localization of *Or42a*-positive-OSNs and *Orco*-positive-OSNs). Scale bar: 20μm. **(E)** Number of *Or42a*-positive-OSNs and *Orco*-positive-OSNs in one maxillary palp of *S. pallida* and *S. flava* (left panel) and ratio between them (right panel). The number of both cell types as well as their percentage ratio was significantly higher in *S. flava* (Mann-Whitney U tests, ** p<0.01 in both cases; n=5 animals/species).

We next generated anatomical maps of sensilla on the anterior part of the maxillary palps of the species of *Scaptomyza* and of *D. melanogaster* for comparison, using diagnostic chemicals (Figures 3A, Figure supplement 4, Supplementary file 10; see methods for more detail). Interestingly, while *D. melanogaster* had a relatively randomized distribution of sensilla on the palps, consistent with previous reports (de Bruyne et al. 1999), all four *Scaptomyza* species exhibited a more organized sensilla pattern, with pb1-like, pb2-like, and pb3-like respectively located medially, distally, and proximally as was indicated in *D. mojavensis* (Crowley-Gall et al. 2016).

The proportions of each type of palp sensilla varied within and between species (Figure 3A and Figure supplement 4). *S. flava* had a larger number of pb1-like sensilla (22.3 ± 1.5, average ± SEM) than of pb2-like (3.7 ± 0.3) or pb3-like sensilla (12.3 ± 1.2; ANOVA with Holm-Šídák’s multiple comparisons test, p<0.0001 and p<0.001). *S. montana* also had a higher number of pb1-like sensilla (17 ± 2.3 pb1-like, 6.3 ± 1.2 pb2-like and 5.6 ± 0.7 pb3-like sensilla; p<0.01 in both cases). In contrast, *D. melanogaster* and the other two *Scaptomyza* species had similar numbers of each sensilla type (range: 5.6–7.3 pb sensilla in *D. melanogaster*, 4.3–6.6 in *S. hsui*, and 7.3–10.7 in *S. pallida;* p>0.05 for the three species). These findings suggest an expansion of pb1-like sensilla in Brassicales specialists over the course of evolution. Moreover, *S. flava* and *S. montana* had an overall larger number of olfactory sensilla on the palps (38.3 ± 0.3 and 29 ± 2.1 pb sensilla, respectively) compared to *D. melanogaster* (20 ± 1.2), *S. hsui* (15.3 ± 0.9), and *S. pallida* (26.3 ± 3.0) (Figure 3A). The largest number of palp sensilla in *S. flava* correlated with the larger size of their palps within *Scaptomyza*: the surface area of one palp of *S. flava* was 3.57 ± 0.32 mm^2^, while that of *S. hsui* and *S. pallida* were respectively 2.37 ± 0.23 mm^2^ and 2.00 ± 0.18 mm^2^; the surface area of the maxillary palp of *D. melanogaster* was 2.99 ± 0.41 mm^2^ (Figure supplement 5). Taken together, these results show that Brassicales plant specialists have a higher proportion of pb1-like sensilla compared to microbe-feeding *Drosophila* and *Scaptomyza*, suggesting that Brassicales plant specialist *Scaptomyza* species may have enhanced capacity to detect volatile ITCs.

### *Sfla Or42a* is triplicated in the genome of *S. flava* and is highly expressed in the maxillary palps.

We next investigated the expression of *Or* genes in the OSNs of sensilla in the maxillary palps across some of the species of interest. Initially, we scanned the known genome sequences of species in the genus *Scaptomyza* and found a duplication of *Or42a* in the lineage leading to all known *Scaptomyza*, while the *D. melanogaster* outgroup had a single *Or42a* homolog (Figure supplement 6). Interestingly, within *Scaptomyza*, *S. flava* exhibited serial triplicates in one of the *Or42a* duplicates, which we named *Or42a2*, *Or42a3*, and *Or42a4* (Figure 3B and Figure supplement 6), whereas *S. montana* retained only two paralogs, like *S. hsui* and *S. pallida*.

To determine the extent of *Or* gene expression, including that of the paralogs of *Or42a*s in the *Scaptomyza* maxillary palps, we conducted species- and sex-specific RNA transcriptome analyses of *S. pallida* and *S. flava* (Figure 3C; Figure supplement 7). We confirmed the expression of *Spal Or42a* and *Sfla Or42a2–4* in the palps. Interestingly, the *Sfla Or42a* paralogs were each expressed at levels comparable to those of other *Or* genes, such as *Or33c*, the homolog of which in *D. melanogaster* is expressed in OSNs of pb2a sensilla (see below). Furthermore, we confirmed the expression of homologs *Or71a* (expressed in OSNs of *Dmel* pb1b), and of *Or33c/Or85e* and *Or46a* (expressed in OSNs of *Dmel* pb2a and pb2b) in both *Spal* and *Sfla* palps. Only *Spal Or42a2* and *Sfla Or42a2–4* were expressed in the OSNs of their respective palp sensilla (pb1-like), whereas *Spal Or42a1* and *Sfla Or42a1* were not. *Or85d* and *Or59c* were respectively expressed in OSNs of *Dmel* pb3a and pb3b but the homologs were unidentified in the genomes of *Scaptomyza* (Goldman-Huertas et al., 2015). Instead, *Or59a1, Or67a1, Or67a2,* and *OrN2a* were strongly expressed in OSNs of both *Spal* and *Sfla,* suggesting that these homologs may be expressed in pb3a or pb3b (Figure 3C). In summary, potential conservation of *Or* expression in pb1 and in pb2 sensilla, and the difference in *Or* expression in OSNs of pb3 between *Drosophila* and *Scaptomyza,* may reflect the similarities and differences in their odorant response profiles (See Figure 2).

### Brassicales specialists have more abundant *Or42a*-positive-OSNs OSNs.

Our transcriptome analysis revealed high expression of *Or42a* triplicates in *Sfla* maxillary palps (Figure 3C), which could be due to high expression of *Or42a* in individual cells and/or a higher number of *Or42a*-positive-OSNs. To distinguish between these possibilities, we quantified the number of *Or42a*-positive-OSNs in the maxillary palps of *Sfla* and *Spal* using hybridization chain reaction RNA fluorescent in situ hybridization (HCR RNA FISH). Due to the high sequence similarity between the paralogs, we designed an RNA probe based on the conserved sequence region of *Sfla Or42a2–4* to visualize all *Or42a2–4* paralog^+^ cells in *S. flava.* Similarly, we designed the *Spal Or42a2* probe to compare the expression of homologs in the maxillary palps of *Sfla* and *Spal* (Figure 3B).

HCR RNA FISH analysis revealed that the maxillary palps of *S. flava* contained more *Or42a*-positive-OSNs than those of *S. pallida,* as well as more *Orco*-positive-OSNs (*Or42a*-positive-OSNs: 34.2 ± 1.53 in *S. flava* vs 13.8 ± 0.74 in *S. pallida*, average ± SEM; *Orco*-positive-OSNs: 96.4 ± 1.91 in *S. flava* vs 53.2 ± 3.00 in *S. pallida*, Mann Whitney U-tests, p<0.01 in both cases, n=5/species, Figure 3D-E). Additionally, the ratio of the total number of *Or42a*-positive-OSNs to the total number of *Orco*-positive-OSNs was higher in *S. flava* (35.6 ± 1.83 % in *S. flava* vs 26.2 ± 1.86 % in *S. pallida*, p<0.01, Figure 3F). This indicates that *S. flava* not only has more *Or42a*-positive-OSNs and *Orco*-positive-OSNs, but also a greater proportion of *Or42a*-positive-OSNs relative to the overall number of OSNs in the maxillary palps.

### Paralog-specific functional evolution of Or42a.

**Figure 4:**
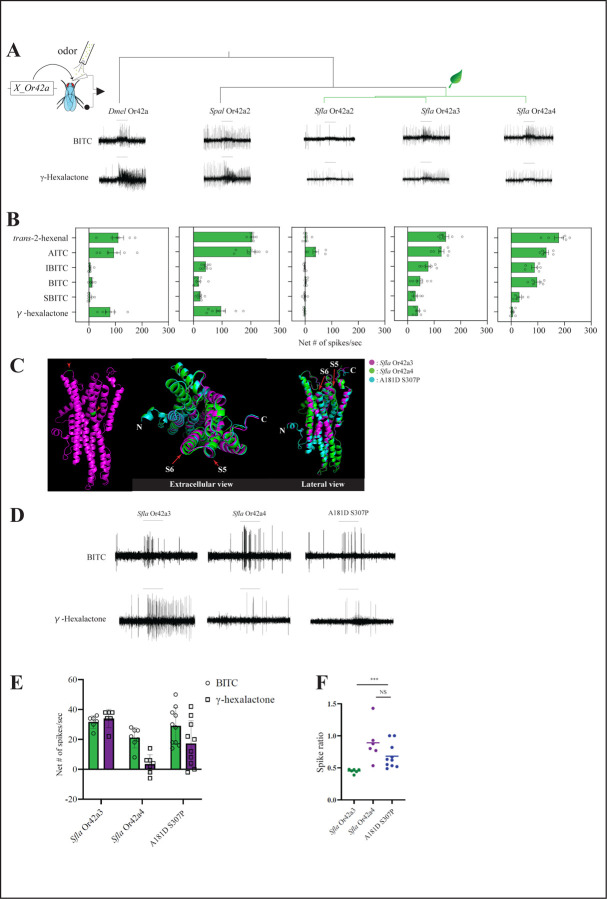
Functional characterization of chimeric Or42a (A181D and S301P) and Or42a from *D. melanogaster*, *S. pallida*, and *S. flava* **(A)** Representative SSR from *Dmel* at1 sensilla OSNs expressing species-specific *Or42a* under the control of *Or67d* ^*Gal4*^ (fly genotype: *UAS-Or42a; Or67d* ^*Gal4*^) in response to BITC or γ-hexalactone stimulation. Horizontal bars indicate the onset of the stimulus and its duration (1 sec). **(B)** Responses of at1 OSNs (n=6–8 sensilla from 3–4 animals/genotype) expressing species-specific *Or42a* upon stimulation with *trans*-2-hexenal (a general leaf odor released upon leaf mechanical damage such as crushing), various ITCs produced by mustard plants (AITC, IBITC, BITC, SBITC), and γ-hexalactone, which is produced by various fruits. All paralogs except *Sfla*Or42a2 responded to trans-2-hexenal. OSNs expressing *Dmel* and *Spal* paralogs responded strongly to γ-hexalactone. OSNs expressing any of the paralogs responded to AITC, but OSNs expressing *Sfla Or42a3* or *Or42a4* additionally responded to other ITCs. **(C)** Schematic representation of chimeric Or42a with two amino acid substitutions (A181D and S301P) in the background of *Sfla* Or42a3. Lateral view of the 3D structure of A181D, S301P predicted by ColabFold (left) using PyMol2.5.3. *Sfla* Or42a3-derived amino acids are indicated in magenta, and the substitutions with *Sfla* Or42a4-derived amino acids are represented in green. The upper and lower sections represent the extracellular and the intracellular region, respectively (C: terminal C section). The middle and right panels respectively display extracellular and lateral views of alignment of the predicted structures of *Sfla* Or42a3, *Sfla* Or42a4, and the chimera. The extracellular region at S5 and S6 (red arrows) highlight the local structural differences between *Sfla* Or42a3 and Or42a4 where the A181D, S301P overlaps with the local structure of *Sfla* Or42a4. **(D-F)** Representative SSR from *Dmel* at1 sensilla expressing heterologous *Sfla* Or42a3, Or42a4, and the chimera (genotypes of *UAS-Or42a/CyO; Or67d* ^*Gal4*^) upon stimulation with BITC or γ-hexalactone (**D**), population responses, with green circles indicating responses to BITC and purple rectangles indicating responses to γ-hexalactone (**E**, n=6–10 sensilla from 3–4 animals/genotype), and response ratio, where green represents *Sfla* Or42a3, purple represents *Sfla* Or42a4, and blue represents A181D S307P (**F**, response to BITC stimulation/response to BITC + response to γ-hexalactone). *p<0.01, Kruskal-Wallis ANOVA followed by Dunn’s multiple comparisons.

We next investigated the functional evolution of *Or42a* triplicates in *S. flava*. To do this, we expressed *Dmel Or42a, Spal Or42a2,* and *Sfla Or42a2–4* in *Dmel* antennal trichoid 1 (at1) OSNs in the background of a null mutation for *Or67d*, the at1 cognate receptor (Kurtovic et al. 2007). We then conducted SSR from at1 using six different odorant stimuli at 1/100 vol/vol: *trans*-2-hexenal, AITC, IBITC, BITC, SBITC, and γ-hexalactone. We found that γ-hexalactone, *trans*-2-hexenal, and AITC strongly activated OSNs expressing *Dmel* Or42a (range: 79–110 spikes/second) or *Spal* Or42a2 (range: 96–205 spikes/sec; Figure 4A-B), while IBITC, BITC and SBITC evoked much weaker responses from these two paralogs (respectively 4.3–11 spikes/sec and 18.3–42.3 spikes/sec). In contrast, OSNs expressing *Sfla* Or42a3 or *Sfla* Or42a4 were very sensitive to IBITC and BITC (range: 87–96.7 spikes/sec in *Sfla* Or42a3; 46–77.7 spikes/sec in *Sfla* Or42a4), which is consistent with the response profiles of OSNs from wild-type *Sfla* pb1a (Figure 2). Notably, OSNs expressing *Sfla* Or42a4 showed only small responses to γ-hexalactone. *Sfla* Or42a2 was only activated by AITC. These results align with the niche difference between microbe-feeding flies and Brassicales specialists. Moreover, our findings reveal the paralog-specific functional evolution of Or42a triplicates in *S. flava*, as different paralogs have evolved distinct sensitivities to various ITC chemicals and fruit odors.

We also stimulated OSNs in pb1a (or pb1a-like) sensilla from the microbe-feeding species (*D. melanogaster, S. hsui* and *S. pallida*) and the Brassicales specialists (*S. flava* and *S. montana*) with various concentrations of odorants from 10^−5^ to 10^−2^ vol/vol. We calculated the odorant concentration required to elicit a biological response halfway between the baseline and the maximum (50% effective concentration values, EC_50_) (Figure supplement 8). The EC_50_ values for γ-hexalactone were consistently lower in the microbe-feeding species in comparison with the Brassicales specialists, indicating a lower sensitivity to γ-hexalactone in the latter. All species had similar EC_50_ values for AITC. OSNs expressing *Sfla* pb1a exhibited a lower EC_50_ value for *trans*-2-hexenal compared to those expressing *Smon* pb1a and the other microbe-feeding species. This heightened sensitivity to this general leaf odor in OSNs of *Sfla* pb1a aligns with its potentially broader host range, as *S. flava* can be reared on plants from other lineages in addition to Brassicales, such as Fabaceae or even Caryophyllaceae in New Zealand (Martin 2004)Maca 1972), whereas *S. montana* appears to be more restricted to Brassicaceae within the Brassicales.

#### AlphaFold2-led screening with ectopic expression of *Sfla* Or42a reveals the molecular changes underlying changes in odor sensitivity

We investigated which amino acid substitutions in *Sfla* O42a3 lead to the respective gain and loss of sensitivity to BITC and γ-hexalactone in *Sfla* Or42a4 (there are total of 32 amino acid differences between *Sfla* Or42a3 and *Sfla* Or42a4, Figure supplement 9A). For this, we predicted the 3D structures of *Sfla* Or42a3 and *Sfla* Or42a4 in the cell membrane and aligned them in 3D space using PyMol, and checked the overlap between Ors (Jumper et al. 2021; Mirdita et al. 2022; Benton and Himmel 2023; Himmel et al. 2023).

One of the most striking differences in 3D structures between *Sfla* Or42a3 and *Sfla* Or42a4 was observed in the S5 and S6 helices in the transmembrane region, which are reported to contain the ligand binding pockets (Figure 4C and Figure supplement 9B; (Del Mármol et al. 2021; Zhao et al. 2024). We therefore substituted each of the 32 amino acids in *Sfla Or42a4* individually with the corresponding residues from *Sfla* Or42a3 *in silico,* predicted the 3D structures of the chimeras, and aligned them with *Sfla* Or42a4 in 3D space until the local structural differences were resolved. The substitutions of A181D and S307P in *Sfla* Or42a3 (hereafter referred to as A181D S307P) resolved the structural differences and overlapped with *Sfla* Or42a4 in 3D space (Figure 4C and Figure Supplement 9B).

We then investigated whether these two amino acid substitutions could account for the *Sfla* Or42a4 enhanced sensitivity to BITC and reduced sensitivity to γ-hexalactone, in comparison with *Sfla* Or42a3. Using the *D. melanogaster* at1 empty neuron system, we functionally characterized the A181D S307P variant, and found that the BITC to γ-hexalactone response ratio (net number of spikes evoked by BITC divided by the sum of the net number of spikes evoked by BITC and γ-hexalactone) was greater than *Sfla* Or42a3 and A181D S307P (Figure 4E-F; Mann-Whitney U test, p<0.001), but not between *Sfla* Or42a4 and A181D S307P (p>0.05). This suggests that the two amino acid substitutions are sufficient to increase the BITC to γ-hexalactone response ratio, by respectively increasing and reducing the sensitivity to BITC and γ-hexalactone in *Sfla* Or42a4. This effect was observed in flies carrying the heterozygous genotype A181D S307P/+ but not in flies with the homozygous A181D S307P genotype (Compare Figure 4E-F with Figure supplement 10). Given that the focal gene is more highly expressed in homozygous flies than in heterologous flies, it is possible that the OSNs’ response to both BITC and γ-hexalactone reached saturation in homozygous flies, obscuring further differences in spike ratios. In summary, our findings demonstrate that A181D and S307P substitutions are critical for shifting the receptor’s sensitivity from fruit odorants to ITCs.

## DISCUSSION:

In this study, we discovered that plant-derived ITCs have a detrimental effect on survival of *D. melanogaster* through volatile exposure. We then found that volatile ITCs are detected by Or42a, which is expressed in the maxillary palps, and that this Or is necessary for the behavioral aversion behavior to volatile ITCs. To our knowledge, Or42a is the first known ITC olfactory detector in *D. melanogaster*. Thus, these results suggest that Or42a works in combination with the “wasabi” taste receptor” TrpA1 and Painless to facilitate adaptive behavioral avoidance of these chemicals in *D. melanogaster* (Al-Anzi et al. 2006; Kang et al. 2010; Mandel et al. 2018). We also conducted molecular evolutionary analyses, site-directed mutagenesis, and functional assays to uncover the molecular mechanisms by which the Brassicales specialist *S. flava* evolved paralogous copies of the homologous *Or42a* genes that we found respond to a broad range of electrophilic compounds emitted by their host plants. Overall, our findings provide new insight into the olfactory mechanisms that allow generalist insects to avoid lethal volatile toxins and into the rapid evolution of an expanded set of more finely tuned *Or*s in specialist insects that detect these volatile host-plant signals.

### Plant-derived volatile AITC is toxic to *D. melanogaster* and its detection and avoidance is mediated by *Or42a*-positive-OSNs

Plants have evolved a diverse array of specialized metabolites that repel or intoxicate insects (Ibanez et al. 2012). Among these, plant-derived electrophilic compounds, including *trans*-2-hexenal and ITCs, are highly reactive (Noge and Becerra 2015; Tocmo et al. 2021). For example, AITC derived from wasabi and other mustard and Brassicales plants causes a highly pungent sensation in humans (Bell et al. 2018). Additionally, ITCs exert toxic effects acutely on insects when volatilized as well (Lichtenstein et al. 1964). Here, we found that volatile AITC also exerts a dose-dependent detrimental effect on *D. melanogaster* adults (Figure 1A). As little as 10 minutes of exposure of low to moderate concentrations of volatile AITC is sufficient to immobilize flies, underscoring the high toxicity of this compound. These findings are consistent with previous findings showing that ingestion of AITC decreases fly survival (Zimmermann et al. 2024) and with earlier studies on the toxic effect of volatile PITC (Lichtenstein et al. 1964).

While TrpA1 pain receptors in *D. melanogaster* mediate the detection of ITCs and other reactive compounds by taste as they do in humans (i.e, by contact, (Kim et al. 2010)), it was unclear whether and to what degree insects have evolved strategies to avoid these compounds via olfaction. Our findings demonstrate that Or42a and its associated OSNs are key components in detecting and inducing olfactory aversion to AITC in *D. melanogaster*, as revealed through exhaustive SSR and olfactory behavioral assays (Figure 1). We found that maxillary palp pb1a OSNs, which express Or42a, respond strongly to olfactory stimulation with AITC in wild-type *D. melanogaster* and TrpA1^−^ loss of function mutants. However, this response was absent in *Or42a*−/− mutants, demonstrating that Or42a is necessary for detecting volatile AITC in pb1a OSNs (Figure 1C-D). Using two different behavioral assays, we found that volatile AITC induces strong behavioral aversion in wild-type and genetic control *D. melanogaster* lines. These flies consumed less sugar solution than *Or42a*−/− mutant flies in presence of volatile AITC, indicating that this compound causes olfactory aversion in a feeding context (Figure 1E). Furthermore, control flies avoided volatile AITC in the positional behavioral assay, while *Or42a*−/− mutants only showed aversion after a long-time exposure (65 minutes) (Figure 1F). The delayed avoidance in *Or42a*−/− mutants could be due to the involvement of additional circuits mediating the aversion to volatile AITC, such as Or7a, which is expressed in ab4a OSNs and was also activated by volatile AITC (Figure 1C), and/or to undescribed olfactory ionotropic receptors that may also respond to AITC. Alternatively, AITC might have adhered to the internal surface of the glass tube during the course of the experiment, leading to taste-mediated aversion via TrpA1.

Dweck et al., (2015) reported that *Dmel* Or42a detects fruit odors and mediates behavioral attraction to these odors. We confirmed this result in our hands by showing that the fruit odor γ-hexalactone strongly activates *Or42a*-positive-OSNs (Figure 4A-B, Figure supplement 2). Moreover, the presence of this odor increased consumption of sugar and induced positional behavioral attraction in control flies, but not in *Or42a*−/− mutants (Figure Supplement 2A-B). These findings may appear contradictory: a single OSN type (*Or42a*-positive-OSNs) mediates aversion to AITC while also driving attraction to γ-hexalactone. This paradox could be resolved considering different levels of OSNs activation, which may result in the recruitment of alternative combinations of glomeruli that are activated by interglomerular local neurons (Kreher et al. 2008; Semmelhack and Wang 2009; Mohamed et al. 2019). Alternatively, distinct subsets of OSNs may be activated by different odorants, leading to behavioral responses of opposite valence (Haverkamp et al. 2018), or a combination of both mechanisms may be involved. Indeed, we found that rescue of Orco-mediated olfactory function specifically in *Or42a*-positive-OSNs was sufficient to induce repellence to AITC (Figure 1G). Additionally, we found that AITC induced the strongest responses in pb1a OSNs but also elicited spiking activity in other OSNs such as ab4a and ab8b, albeit to a lesser degree (Figure 1C). In contrast, γ-hexalactone activated only pb1a OSNs (Figure Supplement 2F). Thus, it’s possible that the combined activation of pb1a OSNs along with ab4a and/or ab8a OSNs leads to repellence, while activation of pb1a OSNs, possibly alongside other uncharacterized OSNs, results in behavioral attraction. Further research is needed to fully elucidate the mechanisms by which a single OSN type may mediate behavioral aversion to one ligand while driving attraction to another.

### Functional evolution of *Or42a* and *Or67b* in *Scaptomyza* mustard plant specialists

Phylogenetic evidence and ancestral state reconstruction indicate that the ancestor of all herbivorous *Scaptomyza* (subgenus *Scaptomyza*) diverged from a microbe-feeding common ancestor and evolved the herbivory habit ca. 10–15 million years ago, giving rise to a monophyletic Brassicales-specialized lineage that includes *S. flava* and *S. montana,* and a Carophyllales-feeding lineage that includes *S. graminum* (Goldman-Huertas et al. 2015; Kim et al. 2021; Peláez et al. 2023). This pattern of specialization on plants that release specialized metabolites that are toxic to many other insects provides a novel niche for host use (Ali and Agrawal 2012), given that specialists also evolved mechanisms to detoxify plant toxins (Gloss et al. 2014; Karageorgi et al. 2019), and may help insects escape pressure from natural enemies like parasitoids (Bernays and Graham 1988). Indeed, drosophilids, including *D. melanogaster*, and *S. flava* use the mercapturic acid pathway to detoxify ITCs (Gloss et al. 2014). Notably, the detoxification enzymes that catalyze this pathway in *S. flava* exhibit a 10-fold higher catalytic efficiency *in vitro* for ITCs compared to other flies, likely allowing them to thrive on Brassicales plants (Gloss et al. 2019).

Specialized insects also often use these toxic compounds as olfactory “tokens” to find their hostplants. Many Brassicales plants release species-specific volatile ITCs (Wu et al. 2021) at particular ratios and concentrations that attract a guild of specialized herbivores through olfaction. For instance, the mustard specialist diamondback moth *Plutella xylostella* uses volatile ITCs to find suitable oviposition sites (Liu et al. 2020). Volatile ITCs are also known to attract *S. flava* females at least in laboratory tests (Matsunaga et al., 2021), and the herbivorous *Scaptomyza* have lost four *Or* that are involved in attraction to yeast-associated volatiles in *D. melanogaster* (Goldman-Huertas et al. 2015; Matsunaga et al. 2021). Given the need for finer olfactory resolution in mustard plant specialist like *S. flava*, we investigated whether the *Or42a* homologs and the Ors they encode in this lineage have undergone differentiation and specialization compared to those of more generalist microbe-feeding species like *D. melanogaster* as well as closer relatives of the herbivorous *Scaptomyza*, which include the microbivores *S. hsui* and *S. pallida*.

To address this question, we started by constructing functional maps of the palp olfactory sensilla across five species that spanned both Brassicales-feeding and microbe-feeding *Scaptomyza* (Figure 2 and 3A, Figure Supplement 3). Our analysis confirmed that *Scaptomyza* species possess three distinct types of sensilla, similar to those found in *D. melanogaster* (Ray et al. 2008; Dweck et al. 2016). We found that the overall tuning patterns were largely conserved between *Scaptomyza* and *Drosophila*, with the exception of OSN responses in pb3a- and pb3b-like sensilla (Figure 2). While OSNs housed in pb1a sensilla from all microbivore and herbivorous species responded to volatile AITC, only OSNs housed in *Sfla* pb1a-like and *Smon* pb1a-like sensilla additionally responded to other volatile ITCs (Figure 2).

Next, we investigated the odor-response profiles of the Or42a paralogous genes. We previously found that triplicated and positively selected *Sfla* Or67b is expressed in antennal OSNs and responds to aromatic and some aliphatic ITCs in a paralog-specific manner. However, all three Or67b paralogs showed a poor response to organosulfur ITCs, including AITC. The *D. melanogaster* and *S. pallida* Or67b copies did not respond to any volatile ITCs (Matsunaga et al. 2021). In this study, on the other hand, we found that all three Or42a paralogs in the Brassicales-feeding species of *Scaptomyza* responded to organosulfur ITCs such as AITC (Figure 4B). Thus, the gene duplications and amino acid substitutions of *Or42a*, along with those of *Or67b*, likely play a crucial role in enabling Brassicales-plant specialist *Scaptomyza* species to detect a wide range of ITCs, including specific blend ratios and concentrations, facilitating effective host plant location. Further refinement could be achieved by changes in processing in downstream olfactory centers, such as those recently reported in the noni specialist *D. sechellia* (Auer et al. 2020).

*Or67b* in *S. flava* is primarily expressed in the antennae (Matsunaga et al. 2021), whereas *Or42a* is expressed in the maxillary palps (Figure 3C). In *D. melanogaster*, OSNs from the maxillary palps have lower sensitivity thresholds to certain host-related compounds compared to antennal OSNs (Dweck et al. 2016). Indeed, we found that the *Sfla* Or42a paralogs are much more sensitive to AITC than its Or67b paralogs [Figure 4, (Matsunaga et al. 2021)]. Odor response redundancy between antennal and maxillary palp Ors could have evolved to further underpin olfactory orientation over both long and short distances in drosophilids (Dweck et al. 2016). Given the proximity of Or42a OSNs to the mouthparts, their activation could potentially modulate feeding behaviors, as suggested in *D. melanogaster* (Shiraiwa 2008). *S. flava* and *S. montana* adult females feed on the juice that seeps into the leaf wounds they create in Brassicales plants before oviposition (Peláez et al. 2022). Given this stereotyped feeding behavior of adult females, while the activation of contact chemoreceptors by ITCs could help females assess the suitability of an oviposition site through taste, flies may also be aided by olfactory activation via the maxillary palps perhaps even before tasting the plants.

### Differential expression of *Or*s in *Scaptomyza*

Our maxillary palp RNA-seq expression data (Figure 3C), combined with electrophysiological results showing similar patterns of odor responses in pb1-like and pb2-like sensilla OSNs (Figure 2), suggest that all *Scaptomyza* species express the following *Or*s in the OSNs of pb sensilla: *Or42a* in pb1a-like, *Or71a* in pb1b-like, *Or33c* and *Or85e* in pb2a-like, and *Or46a* in pb2b-like. This raises the question of which *Or*s are expressed in OSNs of pb3a- and pb3b-like sensilla in *Scaptomyza*. Based on our RNA-seq data, candidates included *Or67a, Or98b, Or59a,* and *OrN2a*, as they were robustly expressed in the maxillary palps (Figure 3C). Among these, only Or67a has been functionally characterized in *D. melanogaster*, and responds to 2-heptanone (Auer et al. 2020). Our SSR experiments showed that 2-heptanone also activated OSNs in pb3a-like (Figure 2), raising the possibility that *Scaptomyza* pb3a-like OSNs express Or67a. Alternatively, other yet uncharacterized Ors responsive to 2-heptanone might be expressed in these OSNs.

Using HCR RNA FISH, we discovered an increase in the number of *Or42a*-positive-OSNs in *S. flava* compared to *S. pallida* (Figure 3C). Similar increases in OSNs which detect odors that bear biological significance for insects have been reported in several *Drosophila* species. For instance, the noni fruit specialist *D. sechellia* and the seasonal specialist of *Pandanus* spp. screw pine fruits *D. erecta* both show an increase in the number of *Or22a*^*+*^ OSNs (Dekker et al. 2006; Linz et al. 2013; Auer et al. 2020). These increases in OSNs have been shown to enhance odor tracking by reducing adaptation in projection neurons in *D. sechellia* (Takagi et al. 2024). We hypothesize that the increase in the number of *Or42a*-positive-OSNs in *S. flava* may similarly contribute to enhance odor sensitivity and tracking during host plant finding, which will require further investigation to address.

### Insight into the binding pocket of Or42a paralogs

Our results demonstrated that the Or42a paralogs from microbe-feeding species show strong responses to both AITC and γ-hexalactone (Figure 4), while the paralogs from the herbivorous *S. flava* show a notable shift in olfactory sensitivity. For example, *Sfla* Or42a3 showed moderate responses to γ-hexalactone (about an order of magnitude lower than those of the Or42a from the microbe-feeding *S. pallida*), and *Sfla Or42a4* showed even less spiking activity (Figure 4D-E). Intriguingly, *Sfla* Or42a4 has a relatively higher sensitivity to BITC compared to Or42a3 (Figure 4B, D and E). These findings align with the fact that *S. flava* has undergone a full transition to the herbivory habit. To explore the mechanisms underlying this shift from fruit-detector to ITC-detector, we employed computational and functional approaches to identify the amino acid substitutions responsible for the differences in odor sensitivities between *Sfla* Or42a3 and Or42a4.

The binding pocket of Ors is located within the transmembrane region, and amino acid substitutions in these regions can alter the ligand’s binding affinity [(Del Mármol et al. 2021; Zhao et al. 2024), although extracellular loops are also suggested to play an important role (Carraher et al. 2015)]. Our AlphaFold2-led screening for amino acid substitutions identified two key substitutions in the transmembrane portion: A181D and S307P (Figure 4C). We substituted these two amino acids and found that OSNs expressing this chimeric Or increased the BITC to γ-hexalactone response ratio, by respectively increasing and reducing the sensitivity to BITC and γ-hexalactone (Figure 4E-F). The substitution of serine to proline (S307P) alters both the charge and the protein structure, which likely accounts for the impact in the notable change in ligand sensitivity. However, the increased BITC to γ-hexalactone spike ratio for was observed only in flies heterozygous for A181D and S307P (Or67d^Gal4^; UAS-A181D S307P/+) and not in the flies homozygous for A181D and S307P (Figure Supplement 10). Given that homozygous flies express A181D and S307P more strongly than the heterozygous flies, it is possible that the OSNs response to both BITC and γ-hexalactone reached saturation in homozygous flies, obscuring further differences in spike ratios. Alternatively, excessive expression of A181D and S307P in cells may have altered the 3D structure of the protein, reverting it closer to the original *Sfla* Or42a3 conformation and restoring the original binding pocket. Nonetheless, the partial rescue we observed in heterologous flies indicates that A181D and S307P are critical for altering the binding pocket structure, enabling the binding pocket of Ors to better accommodate BITC instead of γ-hexalactone.

## CONCLUSIONS:

Taken together, our study reveals that generalist insects have evolved olfactory sensory mechanisms that allow them to detect and avoid plant-derived volatile electrophilic toxins, such as ITCs. In contrast, Brassicales plant specialists have undergone significant evolutionary sensory adaptations for host recognition, including specialized ITC olfactory receptors to aid host plant location. These adaptations involve the triplication of homologous Ors in the antennae (Matsunaga et al. 2021), as well as in the maxillary palps (this study), which collectively expanded the sensitivity and detection range for these and other signature hostplant odorants. Furthermore, our use of AlphaFold2, followed by site-directed mutagenesis and electrophysiology, identified critical amino acid changes for the evolution of specialized odorant receptors. This also demonstrates the utility of machine learning algorithms in evolutionary biology research, particularly in screening and identifying functionally relevant genetic changes for follow-up functional studies.

## Supplementary Material

Supplement 1 **Figure supplement 1: Schematic representations of behavioral assays. (A)** Survival assay. Groups of mated female flies (n=10) were placed in the upper chamber and monitored every 10 minutes in presence of volatile chemicals (4 drops of a 5 μl solution in the lower chamber). The top and the bottom chambers were separated by a fabric mesh, preventing direct contact between the flies and the chemical solutions. **(B)** Feeding assay in presence of odors. Groups of 24 hours wet-starved mated female flies (n=11–15) were transferred to a vial containing a piece of filter paper impregnated with 160 μl of 50 mM D-glucose dyed blue. A small container affixed to the flug vial had a piece of filter paper loaded with 10 μl of an odorant solution or the solvent control, and because the opening was enclosed by mesh, flies could smell but not contact the odor source. After 15 minutes, vials were frozen for at least 60 minutes, the amount of blue dye in the abdomen of each fly in a vial was quantified blind to treatment, and a feeding score was calculated for each vial as explained in materials and methods. **(C)** Positional olfactory assay. Groups of 10–12 non-starved mated females were briefly cold anesthetized in a small piece of clear Tygon tube, which was then used to connect the narrow ends of two cut glass Pasteur pipettes. The distal end of each glass pipette was connected (via a piece of Tygon tube) to a 0.5 mL glass vial containing 10 μL of the odor solution or the solvent control; a piece of fabric mesh prevented insects from entering the vials containing the odor or control solutions. The number of flies in each of the two tubes, as well as in the middle “release” section, were counted every five minutes until minute 35, and in tests with AITC, again at 65 minutes. The % of insects that choose one or the other tube over the total number of insects released, and the % of insects in the tube closest to the odor source over the total number of insects that choose one or the other tube, was then calculated for each time point.
**Figure supplement 2. γ-hexalactone does not affect fly survival and mediates olfactory attraction via activation of Or42a in *D. melanogaster*. (A)** Feeding assay, as in Figure 1E, but using γ-hexalactone (1:50 vol/vol). Flies were allowed to feed for 10 minutes, frozen, and scored blind to treatment. Data were normalized (feeding in presence of the odor/feeding in absence of the odor) to account for potential day-to-day and genotype variability. Normalized feeding of wild-type (Canton-S) flies significantly increased in presence of volatile γ-hexalactone (one-sample signed rank test against expected median=1, p<0.001, n=25), but this enhancement effect was lost in *Or42a*−/− mutants (p>0.05). The horizontal dotted line at 1 indicates neither feeding aversion nor enhancement. **(B)** Percentage of odor choice in the positional olfactory assay, as in Figure 1F, but using γ-hexalactone 1:10 vol/vol. The number of flies in the tube closest to the odor source and in the tube closest to the solvent control was counted every 5 minutes until minute 35. The dotted line at 50% indicates random distribution between the two tubes. Control genotype flies significantly prefer the tube closest to the odor source at various time points (*p<0.05, one-sample signed rank tests against median=50%; p=0.074 and p=0.064 at 15 and 25 minutes; n=19). *Or42a*−/− mutant flies were randomly distributed between the two tubes at all time points (p>0.05 in all cases; n=19). (**C-D):** Percentage of flies that choose either tube in the positional assay [(# of flies in one or the other tube / # of flies released) *100] in tests using AITC 1:500 vol/vol vs. m oil **(C)** or γ-hexalactone 1:10 vol/vol vs DMSO **(D)**. Similar proportions of genotype control and *Or42a*−/− mutant flies choose one or the other tube at all time points in tests with AITC (Mann-Whitney U tests, p>0.05 in all cases), but a significantly larger proportion of control flies choose either tube in tests with γ-hexalactone (*p<0.05, ***p<0.005). (**E)** Survival assay, as in Figure 1A, but using γ-hexalactone at various concentrations from 1/500 to 1/2.5 vol/vol. This odor had no effect on fly mortality within at least 60 minutes. **(F)** SSR from all *D. melanogaster* antennae (ab) and palp basiconic (pb) sensilla to stimulation with γ-hexalactone 1:100 vol/vol. Represented are the control-subtracted net number of spikes and individual data points. Pb1a neurons are the most strongly activated by γ-hexalactone, all other sensilla showed responses ≦20 spikes/second.
**Figure supplement 3: Hierarchical cluster analysis based on odorant response profiles across species. (A)** Hierarchical clusters were constructed using odorant response data from *Dmel, Shsu, Spal, Sfla,* and *Smon* using R studio v1.4.1717. A terminal node corresponds to a single recording from the individual sensilla. N=6–8 sensilla from 3–4 animals. **(B)** Hierarchical clusters were created for each sensilla type, including pb1-like, pb2-like, and pb3-like, using the odorant response (control-subtracted net number of spikes) average from each species as inputs. **(C)** The species phylogeny (left: Kim et al., 2021, eLife) and palp functional dendrogram (right) highlight the relevance of niche differences in shaping odorant responses. The palp functional dendrogram was generated using odorant response average for each species.
**Figure supplement 4. Location and percentages of the three different maxillary palp basiconic (pb) sensilla across species.** Classification of maxillary palp pb sensilla using SSRs and stimulation with diagnostic odorants. Data represents the proportion of pb1 and pb1-like (green), pb2 and pb2-like (red), and pb3 and pb3-like (magenta) in *Dmel, Shsu, Spal, Sfla,* and *Smon*. The total number of sensilla identified in each animal is indicated between parentheses. The schematic positions of pb sensilla in each of the three replicates (R1, R2, and R3) from each species are shown on the right. *Sfla* and *Smon* exhibited a larger number of pb1 compared to the microbe-feeder species, both in absolute numbers and proportions.
**Figure supplement 5: Maxillary palp and body area dimensions across species. (A)** A representative photograph and measurement of a male *Sfla* viewed under a conventional dissection microscope. The areas of the maxillary palp and of the body are respectively indicated by the broken magenta and green lines. Body size was defined as the sum of the head, thorax (excluding legs and wings) and abdomen areas. **(B)** Data (mean ± SEM, symbols indicate individual measurements, n=4 animals/species) represents the palp (top) and body size (middle) areas, along with their ratio (bottom) in *D. melanogaster, S. hsui, S. pallida*, and *S. flava*.
**Figure supplement 6: Or42a gene tree constructed by RAxML.** Maximum-likelihood gene tree with 1000 bootstrap cycles using *Shsu Or42a1*, *Spal Or42a1*, *Sgra Or42a1*, *Smon Or42a1*, *Sfla Or42a1*, *Shsu Or42a2*, *SpalOr42a2*, *Sgra Or42a2*, *Sfla Or42a2*, *Smon Or42a*, *Sfla Or42a2*, *Sfla Or42a3*, *Sfla Or42a4*, and *Dmel Or42a* as an outgroup, generated by RAxML (v8.2.19) (Stamatakis 2014) after alignment by MAFFT v7 (Katoh and Standley 2013). Bootstrap values for the gene trees are denoted only when the values were smaller than 80. The tree was displayed by FigTree v1.4.3 (Rambaut 2009)
**Figure Supplement 7: Maxillary palp RNA seq of *Spal* and *Sfla Or*s.** The heatmap displays Log2[RPM+1] values for *Or*s expression in the maxillary palps of *S. flava* and *S. pallida* across both sexes (n=3). Gray boxes indicate that the corresponding genes were unidentified in the genomes of the species.
**Figure Supplement 8: Dose-dependent responses of OSNs from *D. melanogaster* pb1a and *Scaptomyza* pb1a-like sensilla to stimulation with various odorant concentrations.** Responses of pb1a OSNs across species upon stimulation with various concentrations (ranging from 10^−2^ to 10^−5^ vol/vol) of γ-hexalactone, ethyl butyrate, trans-2-hexenal, AITC, IBITC, BITC, and SBITC (6–8 sensilla from n=3–4 animals/species). The chemical structures of each compound are shown on the left. Half maximal effective concentrations (EC_50_) are shown for each chemical and species. IBITC, BITC, and SBITC were only tested in *Sfla* and *Smon* pb1a, as BITC at 10^−2^ vol/vol failed to activate OSNs in pb1a from microbe-feeding species (Figure 2A). The EC_50_ values for γ-hexalactone were consistently lower in microbe-feeding species compared to Brassicales specialists, suggesting a lower sensitivity in the non-specialist microbivores. *S. flava* exhibited a lower EC_50_ value for *trans*-2-hexenal compared to *S. montana* and the other microbivorous species. This heightened sensitivity to a general leaf odor in *S. flava* aligns with its potentially broader host range than *S. montana*, as it can be reared on plants from other families, such as Fabaceae or Caryophyllaceae (Martin 2004), rather than being strictly limited to Brassicales.
**Figure supplement 9: Screening of candidate amino acids using branch-site test following ColabFold 3D prediction. (A)** Alignment of amino acids of *Spal* Or42a2, *Sfla* Or42a3, and *Sfla* Or42a4, and the A181D S301P chimera. Red arrowheads denote the sites where site-directed-mutagenesis was performed to generate the A181D, S301P chimera. **(B)** Lateral view of alignment of 3D predictions of *Sfla* Or42a3, *Sfla* Or42a4, and the chimera using ColabFold and PyMol 2.5.3. Red rectangles highlight the sites of local structural variance between *Sfla* Or42a3 and Or42a4. The A181D, S301P chimera shows a closer alignment with Or42a4. S5’ and S6’ are enlarged views of S5 and S6, respectively.
**Figure supplement 10: Responses of OSNs expressing homozygous Or42a to stimulation with BITC and γ-hexalactone.** Left: responses of OSNs expressing homozygous *Sfla* Or42a3, *Sfla* Or42a4, or A181D S307P (*UAS-Or42a; Or67d* ^*Gal4*^) to stimulation with BITC and γ-hexalactone (left; n=16–30 from 5–10 animals). Right: ratio between responses to BITC and the sum of the responses evoked by stimulation with BITC and γ-hexalactone. In these homozygous flies, increased BITC sensitivity or decrease γ-hexalactone sensitivity (Figure 4) was not observed, indicating that while two amino acids substitutions (A181D S307P) can change the ligand specificity (Figure 4), additional amino acids are necessary for achieving further robustness in this ligand switch. Kruskal-Wallis test with Dunn’s multiple comparisons, *p<0.05 between *Sfla* Or42a4 and A181D S301P.
