# Odorant receptors tuned to isothiocyanates in *Drosophila melanogaster* are co-opted and expanded in herbivorous relatives

**DOI:** 10.1101/2024.10.08.617316

**Published:** 2025-03-10

**Authors:** Teruyuki Matsunaga, Carolina E. Reisenman, Benjamin Goldman-Huertas, Srivarsha Rajshekar, Hiromu C. Suzuki, David Tadres, Joshua Wong, Matthieu Louis, Santiago R. Ramírez, Noah K. Whiteman

**Affiliations:** 1 Department of Complexity Science and Engineering, Graduate School of Frontier Sciences, The University of Tokyo, Chiba, Japan; 2 Department of Molecular and Cell Biology, University of California Berkeley, Berkeley, CA; 3 Department of Integrative Biology, University of California Berkeley, Berkeley, CA; 4 Department of Molecular, Cellular, and Developmental Biology, University of California Santa Barbara, Santa Barbara, CA; 5 The Biochemistry, Cellular and Molecular Biology Graduate Program, The Johns Hopkins University School of Medicine; 6 Department of Evolution and Ecology, University of California Davis, Davis, CA

**Keywords:** *Drosophila melanogaster*, olfaction, isothiocyanate, odorant receptor, Or42a, *Scaptomyza flava*, herbivory, evolution, Brassicales, AlphaFold2

## Abstract

Plants release volatile compounds that attract mutualists, deter herbivores, and deceive pollinators. Among them are electrophilic compounds such as isothiocyanates (ITCs) derived Brassicales plants that activate TrpA1 pain receptors by contact in *Drosophila melanogaster* and humans. However, it is unclear whether generalist animals evolved strategies to detect these electrophilic compounds via olfaction. To address this, and to understand how specialized insects co-opted these toxic compounds as hostplant signatures, we studied generalist micro-feeding (*D. melanogaster* and *Scaptomyza pallida*) and herbivorous mustard specialist drosophilid flies (*S. flava* and *S. montana*). In behavioral assays, *D. melanogaster* exposed to volatile allyl isothiocyanate (AITC) were rapidly immobilized, demonstrating the high toxicity of this compound to non-specialists. Through single sensillum recordings (SSR) from olfactory organs and behavioral assays, we found that the Odorant receptor 42a (Or42a) is necessary for volatile AITC detection and behavioral aversion. RNA expression following heterologous expression showed that lineage-specific, triplicated *S. flava* Or42a proteins exhibited paralog-specific broadened ITC sensitivity. AlphaFold2 modeling followed by site-directed mutagenesis and SSR identified two critical amino acid substitutions that changed Or sensitivity from fruit-derived odors to ITCs during the evolution of *Or42a*. Our findings suggest that ITCs, which are toxic to most insects, can be detected and avoided by non-specialists like *D. melanogaster* through olfaction. In the specialist *S. flava*, paralogous *Or42a* copies experienced gene duplication and amino acid substitutions resulting in expanded ITC sensitivity. Thus, insect olfactory systems can rapidly adapt to toxic host plant niches through co-option of chemosensory capabilities already present in their ancestors.

## INTRODUCTION:

Plants have evolved the ability to synthesize a diverse array of potentially toxic specialized metabolites that can provide resistance against insect herbivory. In turn, herbivorous insects have evolved diverse morphological, physiological, and behavioral counter-strategies to avoid these chemicals if encountered, or to mitigate their effects if ingested (Mithöfer and Boland 2012). Some herbivores even co-opt these plant toxins as oviposition or feeding stimulants and chemical defenses of their own. For example, monarch butterfly (*Danaus plexippus*) larvae evolved target site insensitivity in the sodium potassium ATPase against the cardenolides released from milkweed (*Asclepias* spp.) host plants upon wounding (Dobler et al. 2012). The caterpillars sequester these heart poisons, which serve as effective defenses against predation throughout the life of the insect (Reichstein et al. 1968). Although cardenolides have one major molecular target in insects, many plant toxins are far more promiscuous in their modes of action, which presents a different “evolutionary hurdle” (Southwood 1972) to herbivores. Among them are various alkaloids, terpenes, and green leaf volatiles (GLVs) electrophilic compounds, such as piperine from black pepper, caryophyllene from cloves, and *trans*-2-hexenal from a wide range of leaves and grasses (Noge and Becerra 2015; Yaffe et al. 2015; Iorio et al. 2022). These compounds intoxicate or deter herbivores and pathogens by interacting with nucleophilic sites in biological molecules, rendering them toxic or inhibitory (War et al. 2012).

Brassicales plants that include mustards (Brassicaeae) have evolved a sophisticated chemical defense system that produces promiscuous electrophilic toxins (Ahuja et al. 2010). These plants produce non-toxic glucosinolates from various amino acid precursors, some of which are hydrolyzed *in planta* to form toxic compounds that can include electrophilic isothiocyanates (ITCs) (Hopkins et al. 2009). ITCs are defined by a R–N=C=S functional group wherein the carbon is attacked by nucleophiles such as the sulfhydryl groups of cysteines. For example, allyl isothiocyanate (AITC) is derived from radish [*Raphanus sativus* (Cuellar-Nuñez et al. 2022)], while butyl isothiocyanate (BITC) and other ITCs bearing similar structures are derived from cabbage [*Brassica oleracea* (MacLeod et al. 1989)]. The chemical diversity of glucosinolates allows Brassicales plants to effectively deter a wide array of herbivorous insects because different species have different mixtures of glucosinolates, making it more difficult for insects to adapt (Winde and Wittstock 2011).

One herbivorous insect lineage that has evolved to cope with more promiscuous toxic plant metabolites like ITCs are obligated leaf-mining drosophilid flies in the genus *Scaptomyza* (e.g., *S. flava* and *S. montana*: hereafter *Sfla* and *Smon*, respectively) which are specialized primarily on Brassicales plants. *Sfla* likely has a broader host range, as it can be reared on plants from other lineages in addition to Brassicales, such as Fabaceae or even Caryophyllaceae in New Zealand (Martin 2004; Maca 1972), whereas *Smon* appears to be more restricted to Brassicaceae within the Brassicales and may require plants that produce indolic glucosinolates specifically (Gloss et al. 2017). *Scaptomyza* species form a clade that is phylogenetically nested within the paraphyletic *Drosophila* subgenus. Gloss et al. (2014) found that yeast-feeding *Drosophila melanogaster* (hereafter *Dmel*) uses promiscuous glutathione *S*-transferases (GSTs) in the gut to detoxify dietary ITCs released from glucosinolate-bearing Brassicales plants like thale cress (*Arabidopsis thaliana*), broccoli (*Brassica oleracea*) and wasabi (*Eutrema japonicum*). Further, they characterized a set of *Scaptomyza*-specific GSTs that experienced rapid gene duplication and non-synonymous changes during the evolution of herbivory, resulting in among the most efficient GSTs known from animals in detoxifying ITCs (Gloss et al. 2014; Gloss et al. 2019). Additionally, Brassicales-feeding *Scaptomyza* flies may minimize their exposure to glucosinolate-derived toxins by selectively ovipositing on leaves that contain lower glucosinolate concentrations (Humphrey et al. 2016).

Insects can behaviorally avoid toxic chemicals through gustation and/or olfaction (e.g. Bernays and Chapman 1987; Stensmyr et al. 2012; Scott 2018; Chen and Dahanukar 2020; Dweck and Carlson 2020). In *Dmel*, physical contact of ITCs by the tarsi and/or proboscis induces behavioral aversion through gustatory receptor cells that express the nociceptive “wasabi receptor” TrpA1 and Painless (Al-Anzi et al. 2006; Kang et al. 2010; Kim et al. 2010; Mandel et al. 2018). Volatile exposure to extracts containing phenethyl isothiocyanate (PITC) from turnip or rutabaga kills *Dmel*, suggesting that volatile PITC is toxic to these insects at biologically meaningful concentrations (Lichtenstein et al. 1964). In addition, volatile allyl isothiocyanate (AITC) causes behavioral aversion in fire ants (*Solenopsis invicta*) (Hashimoto et al. 2019). All these findings raise the possibility that insects have evolved olfactory sensory neurons (OSNs) that detect plant-derived electrophilic compounds such as ITCs, leading to olfactory-mediated behavioral avoidance in generalists, as has been found in the gustatory system.

*Scaptomyza* includes species that are both non-herbivorous (e.g., microbe-feeding) and herbivorous (Aguilar et al. 2024). However, the most recent common ancestor of the herbivores is hypothesized to have been microbe-feeding based on ancestral state reconstruction (Whiteman et al. 2011; Whiteman et al. 2012; Peláez et al. 2023). Of particular interest are the Brassicales plant specialists *Sfla* and *Smon*, which are closely related species in the exclusively herbivorous subgenus *Scaptomyza* of the genus *Scaptomyza* (Kim et al. 2021). Additionally *Or85d, Or42b, Or9a* and *Or22a*, which in *Dmel* mediate the olfactory detection of fermentation products of brewer’s yeast and other microbes, were lost in a stepwise fashion during the evolution of herbivory in *Scaptomyza* (Goldman-Huertas et al. 2015). Furthermore, the *Sfla* paralogs of the antennal odorant receptor Or67b, which in *Dmel* is encoded by a single *Or67b* copy and responds to green leaf volatiles such as *trans*-3-hexenol, respond to a subset of ITCs only in *Sfla*, not *Dmel* (Matsunaga et al. 2021). Although there has been some progress, whether and to what extent generalist insects avoid volatile electrophilic compounds like ITCs on the one hand, and how evolution has reshaped the sensory receptors of specialists to facilitate aversion and/or attraction on the other, is still largely unknown, yet is a central problem in understanding how organisms invade toxic niches and host herbivorous insects specialize on toxic host plants.

We addressed this problem in *Dmel* and a set of *Scaptomyza* spp. exhibiting a gradient in microbe-feeding and plant-feeding habits. We first recapitulated the experiments of Lichtenstein et al. (1964) with the more commonly used AITC instead, and found that *Dmel* is rapidly immobilized (likely intoxicated) by exposure to moderate concentrations of volatile AITC. This indicates that *Dmel* may have evolved odorant receptors that respond to and release aversive behaviors toward volatile ITCs. We found that Or42a, which is expressed specifically in *Dmel* maxillary palp basiconic sensilla 1a (pb1a) OSNs (Ray et al. 2007), is highly sensitive to volatile AITC and is necessary for behavioral aversion to this compound. We discovered that the mustard plant specialist *Scaptomyza* species *Sfla* and *Smon*, but not microbe-feeding *Scaptomyza* and *Dmel*, have expanded their ITC sensitivity range. In *Sfla*, this was coupled with a concomitant increase in the number of *Or42a*-positive-OSNs, and with a triplication of the *Or42a* gene. We then used site directed mutagenesis and found that via just two amino acid substitutions, one of these Or42a paralogs in *Sfla* switched its sensitivity from odors that activate the Or42a of microbe feeders to odors that activate mustard specialists, paralleling the niche shift of the mustard-feeding *Scaptomyza*. Taken together, these results illustrate how plant-derived volatile toxins like electrophilic ITCs can negatively impact insect behavior and fitness, how the insect olfactory system detects them leading to behavioral avoidance, and how host-plant specialization reshapes the organization and molecular function of toxin-detecting Ors.

## MATERIAL AND METHODS:

### Fly Husbandry

Microbe-feeding *Dmel* was reared on cornmeal medium. Microbe-feeding *S. pallida* (hereafter: *Spal*, subgenus *Parascaptomyza*) and *S. hsui* (hereafter: *Shsu*, subgenus *Hemiscaptomyza*) were reared in vials of cornmeal molasses media covered with a mixture of Carolina biological supply instant *Drosophila* media (Burlington, NC) mixed with blended spinach leaves, and then covered with a layer of defrosted frozen spinach leaves. The obligate leaf-miners *Sfla* and *Smon* (subgenus *Scaptomyza*) were cultivated on potted fresh laboratory-grown *Arabidopsis thaliana* Col-0. Isofemale lines of microbe feeding *Shsu* and *Spal*, as well as the herbivorous *Smon*, were collected along Strawberry Creek on the UC-Berkeley Campus in Berkeley, California, USA (Kim et al. 2021), and a line of *Sfla* was collected from a meadow near Dover, New Hampshire, USA. All species were kept at 23±2°C and 60% relative humidity in a 10L:14D light cycle under fluorescent lights. The following lines (stock #) were obtained from the Bloomington *Drosophila* Stock Center: *Or42a*−/− (60821), *Or42a-Gal4* (9970), *w^1118^* (3605), *TrpA1^1^* (26504), *Or35a*−/− (91811) *Or7a*−/− (91811), and a genetic background control for the three Or null mutant lines (68384). The *Or35a*−/− line (10564) was obtained from the Korea Stock Center. The *Or67d^Gal4^* line was a gift from the laboratory of Barry J. Dickson.

### Single sensillum recordings (SSRs)

One-five days old fed female flies were prepared for SSR as described in Matsunaga et al. (2021). Briefly, a silicon tube delivering a constant flow of charcoal-filtered air (16 ml/min, measured using a flowmeter, Gilmont Instruments, USA) was placed near the fly’s head capsule, and the tip of the stimulation pipette (50 ml) was inserted into the constant air stream. The stimulation pipette contained a 0.5 cm x 5 cm piece of filter paper loaded with 20 μl of an odorant solution or the solvent control. A pulse of clean air (duration = 1 sec) was delivered to the stimulus pipette using a membrane pump operated by a Stimulus Controller CS 55 (Syntech, Buchenbach, Germany). Sensilla identification was conducted using the following diagnostic odorants (as described in Dweck et al. 2016; Gonzalez et al. 2016), all >95% pure (Sigma-Aldrich, St. Louis, MO, USA): ethyl acetate (CAS# 141-78-6) for identifying *Dmel* pb1a; AITC (CAS # 57-06-7) for identifying *Scaptomyza* pb1a-like olfactory sensory neurons (OSNs); 4-ethylguaiacol (CAS# 2785-89-9) for identifying *Dmel* and *Scaptomyza* pb1b-like OSNs; fenchone (CAS# 1195-79-5) for identifying *Dmel* and *Scaptomyza* pb2a OSNs; guaiacol (CAS# 90-05-1) for identifying *Dmel* and *Scaptomyza* pb2b OSNs; phenethyl acetate (CAS# 103-45-7) for identifying *Dmel* pb3b, *Sfla* pb3b, and *Smon* pb3a-like OSNs; and 2-heptanone for identifying *Shsu* and *Spal* pb3a-like OSNs. All odorants were diluted in mineral oil (CAS# 8042-47-5) except γ-hexalactone (CAS # 695-06-7), which was diluted in dimethyl sulfoxide (DMSO, CAS# 67-68-5) because it did not dissolve completely in mineral oil and sometimes produced response artifacts. Odorants were diluted to 1:100 vol/vol for stimulation unless otherwise noted. Supplementary file 1 lists all the chemicals used in this study.

The “net number of spikes/second” was obtained by counting the number of spikes originating from the OSN of interest within a 0.5-second timeframe which started 0.2 seconds after the onset of stimulation. This count was then adjusted by subtracting the background spiking activity (# of spikes within a 0.5 second interval preceding the onset of the stimulation), and then doubled to represent the number of spikes/second. In all figures, unless otherwise stated, we represent the “control-subtracted net # of spikes/sec” to odorant stimulation, calculated by subtracting the average net # of spikes/sec in response to the solvent control (mineral oil or DMSO) from the net # of spikes/sec evoked by each odorant stimulation. Control-subtracted spike data are compiled in Supplementary file 2. The Butyl-ITC (BITC) to γ-hexalactone spike ratio (Figure 4F and Figure Supplement 11) was calculated as: net # of spikes/sec evoked by BITC / (net # of spikes/sec evoked by BITC + net # of spikes/sec evoked by γ-hexalactone). We used this denominator for the ratio because the control-subtracted net # of spikes upon γ-hexalactone stimulation occasionally produced negative values (likely a response to the solvent control).

Half maximal effective concentrations (EC_50_) were calculated using *Quest Graph*^™^ *EC50 Calculator*. (AAT Bioquest, Inc., 4 Mar. 2025, https://www.aatbio.com/tools/ec50-calculator). We primarily used the two-parameter feature with normalization, where responses were normalized to the largest response within the same chemical-species pair, with the minimum set to 0. This approach was chosen because the lowest tested concentration (10^−5^) still elicited non-zero spike activity (>10) in some chemical-species pairs. The four-parameter method without normalization, in which the maximum and minimum responses were free parameters, was used in cases where the two-parameter method failed to fit a logistic regression (summarized in Supplementary file 2) or when analyzing sec-Butyl ITC (SBITC) data. For SBITC, the four-parameter method was necessary because the highest tested concentration (10^−2^) did not reach saturation (<100 spikes). When both the two-parameter and four parameter methods failed due to lack of convergence at the lowest concentration (10^−5^), resulting in a calculated value of 0, we instead used the minimum value observed within that chemical-species group.

The *Or67d*^GAL4^ line was used to generate flies expressing *Or42a* homologs in the at1 “empty neuron” system, as described in (Kurtovic et al. 2007).

The spike amplitude differences between the two types of OSNs housed in pb3a and pb3b were less distinct in *Scaptomyza*, and therefore we could not completely rule out the possibility that we occasionally erroneously assigned spiking to each of these two OSNs types.

### Immobility assay

To investigate the effect of AITC volatiles in wild-type *Dmel* (Canton-S strain), we used a 9 cm diameter plastic petri dish (Nunc, Denmark) with a piece of fabric mesh placed horizontally between the base and the lid, creating two chambers (Figure Supplement 1A). The upper chamber housed 8-10 male flies 3-5 days old, and the lower chamber contained four 5 μl drops of the odor solution (or the solvent control) evenly dispersed. Because the mesh prevented the flies from reaching the bottom chamber, insects were exposed to the volatile chemicals but could not directly contact (i.e. taste) the odor solution, unless the molecules adhered to the walls of the chamber after volatilizing. After each test started, we counted the number of mobile flies every 10 minutes up to one hour and calculated the percentage of mobile flies at each time point. Flies exhibiting no movement for >30 seconds were likely intoxicated. AITC or γ-hexalactone were diluted in either mineral oil or DMSO at various concentrations. Mobility data analysis was performed using the log-rank (Mantel-Cox) test (Mantel 1966). The complete immobility assay dataset is compiled in Supplementary file 3.

### Consumption assay

Groups of 2-4 days old mated female flies (n=11-15) were wet-starved for 24 hours, and then transferred to a vial containing a piece of filter paper (2.7 cm diameter, Whatman, cat. No 1001 125) impregnated with 160 μl of 50 mM D-glucose (Sigma-Aldrich, USA) dyed blue with Erioglaucine (0.25 mg/ml, Sigma-Aldrich, St. Louis, MO, USA) (Reisenman and Scott 2019) (Figure Supplement 1B). Flies were allowed to feed for 15 minutes (10 minutes in tests with γ-hexalactone), vials were frozen (>60 minutes), and the amount of blue dye in the flies’ abdomen was scored blind to treatment (see below). The odor source consisted of a strip of filter paper (0.25 cm wide x 1.5 cm long) impregnated with either 10 μl of an odorant solution (test) or 10 μl of the solvent (control), which was placed inside a container (1.3 cm long x 0.75 cm diameter) with a meshed bottom affixed to the vial’s flug (Figure Supplement 1B). This allowed diffusion of odors into the fly vial but prevented flies from contacting the odor source. Control tests, with vials containing food solution but only the solvent control inside the meshed container, were conducted in parallel with experimental tests to control for fly cohort and day-to-day variability.

Food consumption was estimated by scoring individual flies in each vial blind to treatment using the following five-point scale (Reisenman and Scott 2019): 0 (no dye = no food), 0.25 (“trace” of blue dye), 0.5 (up to ¼ of the abdomen dyed blue), 1 (more than ¼ but less than ½ of the abdomen dyed blue), and 2 (more than ½ of the abdomen dyed blue). For each vial, a single feeding score value was calculated as: [(0 x n_0_ + 0.25 x n_0.25_ + 0.5 x n_0.5_ + 1 x n_1_ + 2 x n_2_) / N], where n_(0-2)_ denotes the number of flies in each score category, and N the total number of flies/vial. Feeding scores from each test vial (flies tested in presence of odor) were normalized to the averaged feeding score of control vials (flies of the same genotype tested in absence of odor) assayed on the same day. Normalized feeding scores for each genotype and odor were compared against the null hypothesis (median feeding score = 1) using one sample signed rank tests. That is, medians not different from 1 indicate that the odorant did not reduce neither enhanced consumption, while medians significantly less than 1 or more than 1 respectively indicate feeding aversion or enhancement. Normalized data from control and mutant flies were compared using Mann-Whitney U tests. In all cases results were considered statistically significant if p<0.05. The consumption assay data is compiled in Supplementary file 4.

### Positional olfactory assay

To study the olfactory orientation of insects towards odors, we conducted assays with non-starved 3-4 days old mated females (Figure Supplement 1C). Flies (n=10-12/test) were anesthetized on ice (5-7 minutes) and placed in a small piece of clear Tygon tube, capped in both sides with a conical PCR plastic Eppendorf. After another about 4-5 minutes, the Tygon tube with the anesthetized flies was uncapped and connected to the cut ends of two glass Pasteur pipettes and the assay started; flies usually resumed activity after about 3-4 minutes. Each of the two opposite ends of the pipettes were respectively connected (via a small piece of Tygon tube) to a 1.75-ml glass vial containing 10 μl of the odor solution (AITC 1: 500 vol/vol, or γ-hexalactone 1:100 or 1:10 vol/vol) or 10 μl of the control solvent (mineral oil, or DMSO). Tests with apple cider vinegar used 30 μl instead, and water was used as a control. The distal ends of the pipettes were separated from the glass vials with a small piece of fabric mesh, which allowed the odorant to diffuse into the pipette while also preventing insects from contacting the odor source (Figure Supplement 1C). The odor and control sides were switched between assays. Assays were conducted on a white surface under white light at 21-24 °C, about 2-6 hours after lights on. Once each assay started, the number of flies in the pipette closest to the vial with the odor (referred to as “odor side” or “odorous tube” for simplicity), in the pipette closest to the vial with the solvent control (“control” side/tube), and in the Tygon tube that connected both pipettes (release site) were counted every 5 minutes until minute 35, and then again at 65 minutes in the case of tests with AITC. For each assay, we calculated the % of insects that made a choice for one or the other tube as: [(#of insects in the odor side + # of insects in the control side) / total number of insects released)] x 100. The percentage of insects that choose the odorous tube was calculated based on the total number of insects that made a choice as: [# of insects in the odor side / (# of insects in the odor side + # of insects in the control side)] x 100. Assays in which less than 40% of insects made a choice for either side at all time points were discarded (<5% of assays). For each fly genotype and odorant, the % of insects that choose the odor side at each time point was compared against the median value expected under the hypothesis that insects distributed at random between the two tubes (50% of insects in each tube) using one-sample signed rank tests. Thus, we assessed whether the insects significantly avoid the odorous tube (if median<50%), prefer it (if median>50%), or showed a random preference. Also, at each time point and for each odor (and concentration when applicable), the responses of the null mutants (*Or42a*−/−, *Or7a*−/− and *Or35a*−/−) and their genetic background control (listed above) were compared via Mann-Whitney U tests. In all cases results were considered significant if p<0.05. In most cases we used two-tailed tests (e.g. for testing median_1_ ≠ median_2_), but in a few cases we used one-tailed tests (e.g. for testing median_1_ > median_2_ or median_1_<median_2_, Figure Supplement 2G–I). The positional olfactory assay data is included in Supplementary file 5.

### RNA-sequencing of maxillary palps

Newly emerged adults of *Sfla* and *Spal* were collected from our colony and kept in humidified vials with 10% honey water until dissection, to minimize potential differences in nutrition resulting from differences in the two species’ larval diet. Three to ten days old flies were anesthetized with CO_2_, and their maxillary palps were hand-dissected using forceps. Approximately 100-120 flies were pooled for a single sample. The dissected tissues were directly collected in LB+TGA lysis buffer from Reliaprep RNA Tissue Miniprep System (Promega, USA), and homogenized using a Biomasher Standard homogenizer (Takara Bio Inc., USA) in a dry ice ethanol bath. The sample lysates were stored at −80°C until RNA extraction. Total RNAs were extracted from the lysates using ReliaPrep RNA Tissue Miniprep System (Promega, USA) according to the manufacturer’s protocol, and quantified using a Qubit RNA High Sensitivity kit (Thermo Fisher Scientific, USA). Library preparation was performed at the Functional Genomics Laboratory (FGL) at UC Berkeley. Due to the low yields of our maxillary palp-derived total RNAs, cDNA libraries were first produced by Takara SMART-Seq mRNA Ultra-low input RNA kit (Takara Bio Inc., USA) with 8 cycles of PCR for the amplification, and then processed by KAPA HyperPrep kit for DNA (Roche Sequencing, USA) with 9 cycles of PCR for attaching in-house sequencing adapters and index primers. cDNA libraries were then sequenced on an Illumina NovaSeq 6000 150 PE S4 flowcell, targeting 25M read pairs per library by the UC Berkeley Vincent J. Coates Genomics Sequencing Laboratory. For read mapping, we used previously reported reference genome assemblies and gene annotations from *Sfla* (Peláez et al. 2023) and *Spal* (Kim et al. 2021) for subsequent bioinformatic analyses. Raw RNA-seq reads were filtered using Fastp v0.21.0 (Chen et al. 2018) and mapped to the respective reference genomes using STAR v2.7.1a (Dobin et al. 2013) to generate multiple alignment (BAM) files, which were then converted to read count data using HTseq v0.9.1 (Anders et al. 2015). Count data for the Or gene family were converted to RPM (reads per million; Supplementary file 6).

### HCR RNA FISH

One-to-four-days-old female *Sfla* and *Spal* were collected and anesthetized with CO2. Whole mouthparts were removed and immediately placed in 2 mL of fixative (4% vol/vol paraformaldehyde in 1x phosphate buffered saline with 3% vol/vol Triton X-100 added, PBST) in LoBind Eppendorf tubes, and fixed for 22 hours at 4°C on a nutator.

Fixative was removed and replaced with 2 mL of cold (4°C) 3% PBST, and tubes were agitated on a nutator for 5 minutes. This step was repeated once more and then again with cold 0.1% PBST; samples were then washed with 1 mL of cold 0.1% PBST 4 times and agitated on a nutator (5 min/wash). PBST was removed and replaced with 300 μL of warm (37°C) HCR RNA FISH probe hybridization buffer (Molecular Instruments, Inc., Los Angeles, CA) and incubated for 30 minutes. The probe hybridization solution was prepared by adding 5μL of 1μM of probe B1-*Spal Or42a* and probe B2-*Spal Orco* (or probe B1-*Sfla Or42a* and probe B2-*Sfla Orco*) to 300 μL of pre-heated probe hybridization buffer for the *Spal* or the *Sfla* tissues and kept at 37°C. Finally, the buffer was replaced with preincubated probes in hybridization buffer and kept at 37°C for 22 hours.

Samples were next washed 3 times with 500 μL of preheated (37°C) probe wash buffer for 5 min on a heating block. Ten μL of 3μM amplification hairpins one and two of b1-488 and b2-637 HCR amplifiers (Molecular Instruments, Inc., Los Angeles, CA) were snap cooled on a thermocycler at 95°C for 90 secs and then at 20°C for 30 min, and covered with aluminum foil to prevent fading. Samples were kept at room temperature and washed 3 times with 500 μL of 0.75M NaCl, 75mM sodium citrate and 0.1% Tween-20 vol/vol (SSCT) for 5 minutes, and then incubated in 500 μL amplification buffer (Molecular Instruments, Inc., Los Angeles, CA) for 30 minutes. Snap cooled hairpins were added to two vials containing 300 μL of amplification buffer for each species. The amplification buffer was removed from the vials and replaced with the hairpin solution; tubes were covered in aluminum foil and incubated at room temperature for 22 hours.

Samples were washed twice with 500 μL of SSCT solution for 5 minutes, twice for 30 minutes, and finally washed again for 5 minutes. Samples were then stained with 300 nM DAPI stain in 0.1% PBST for 15 minutes and then washed 3 times with 0.1% PBST for 5 minutes. Tissues were transferred to a microscope slide and mounted in a drop of ProLong diamond antifade mounting (Life Technologies Corp., Eugene, OR) and stored at 4°C until examination. Confocal imaging of fixed samples was performed using a Zeiss LSM 880 microscope in the AiryScan mode. Raw images were processed using Zeiss ZEN Black software. Orco-positive cells (visualized with the 488 nm laser) and Or42a-positive cells (visualized with the 633 nm laser) were manually counted using the “Cell Counter” plugin in Fiji (ImageJ) software. Supplementary file 7 contains cell counts of RNA FISH.

### *Scaptomyza Or42a* gene cloning and generation of *UAS* lines

RNA was isolated from 15-25 days laboratory-reared adults of both sexes of *Spal* and *Sfla* using the ReliaPrep Miniprep system (Promega, W.I., USA). Complementary DNA (cDNA) was synthesized using the qScript cDNA Supermix (Quantabio, Beverly, MA, USA). The paralogs were amplified via touchdown PCR, using primers that target the highly variable regions of the 5’ and 3’ untranslated regions (UTRs). The single *Or42a2* gene of *Spal* was amplified using touchdown PCR with primers targeting the coding region (Q5 DNA Polymerase, #M0491, NEB) (Supplementary file 8). All PCR products underwent gel purification (11-300C, Zymo Research) and were subsequently cloned using the Gibson Assembly (E2611S, NEB) following the manufacturer’s instructions into the UAST-attB vector (DGRC Stock 1419; RRID:DGRC1419, Bischof et al. 2007). The ligation products were transformed into DH5α competent cells (T3007, Zymo Research). After confirming the sequences using Sanger sequencing, the 5’ and 3’ UTRs of the plasmids containing the *Sfla Or42a* paralogs were removed. This was achieved by first amplifying the coding region using PCR, followed by gel purification and ligation back into UAST-attB vector via Gibson Assembly. The resultant plasmids were Sanger sequence-verified. Finally, these plasmids were microinjected into the attP40 site (P{nos-phiC31\int.NLS}X, P{CaryP}attP40, line # 25709, Rainbowgene) to create transgenic lines for *Spal Or42a*, *Sfla Or42a2*, *Sfla Or42a3* and *Sfla Or42a4*. All lines were confirmed by sequencing prior to experiments.

### Screen of candidate amino acids using AlphaFold2 3D structural prediction

CDS of *Sfla Or42a3* and *Sfla Or42a4* were confirmed by palp RNAseq data using IGV_2.16.0. CDSs of *Sfla Or42a3* and *Sfla Or42a4* were then used as inputs into ColabFold (Jumper et al. 2021; Mirdita et al. 2022). Then the output models ranked first (rank1) were selected, visualized, and 3D-aligned by using PyMol2.5.3. We focused on the regions where predicted Local Distance Difference Test (pLDDT) scores exceed 70, as structures with lower pLDDT scores are often unreliable (Mariani et al. 2013). We focused on the S5-S6 transmembrane helices because previous studies suggested that the binding pockets of odorant receptors are located in the transmembrane region (Butterwick et al. 2018; Del Mármol et al. 2021; Zhao et al. 2024), and our amino acid alignment of *Sfla* Or42a3 and *Sfla* Or42a4 provided the highest root mean square deviation (RMSD) scores in this region. In silico substitutions of amino acids were performed individually on *Sfla* Or42a3, and the resulting sequences were then re-input into ColabFold, the model with rank1 was selected, and the structures were 3D-aligned with that of *Sfla* Or42a4. This process of in silico mutation and alignment was repeated until the RMSD scores for the S5-S6 region were reduced to a value comparable to the other region (~0.1 Å). All pdb files used in this study are included in Supplementary file 9.

### Site-directed mutagenesis

Conventional PCR was conducted using plasmids of *Sfla Or42a3* as backbone (Q5 DNA polymerase, #M0491, NEB; Supplemental File 1). Primers were designed to introduce the point mutations (Supplementary file 1). The PCR product underwent gel purification (11-300C, Zymo Research) and the methylated plasmids were digested with DpnI for 1 hour at 37°C (QuickChange, Agilient Technology, USA). Ligation was performed by incubation at 16°C for 30 minutes (DNA ligation kit Mighty mix, Takara, Japan) and the products were transformed into DH5α competent cells (Takara, Japan). After confirming the sequence using Sanger sequencing, the plasmid was microinjected into the attP40 site (P{nos-phiC31\int.NLS}X, P{CaryP}attP40, line # 25709, Rainbowgene) to create transgenic A181D S307P fly. The line was confirmed by sequencing prior to experiments.

## RESULTS

### Volatile AITC rapidly immobilizes *Dmel*

**Figure 1:**
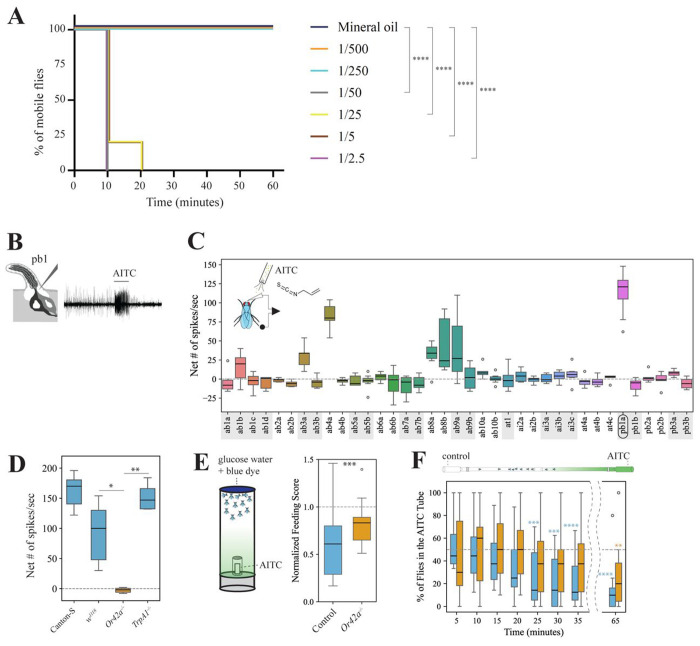
*Drosophila melanogaster* Or42a mediates volatile toxicity of allyl isothiocyanate (AITC) and behavioral aversion to this compound **(A)** The toxic effect of various concentrations of volatile AITC to *D. melanogaster (Dmel)* flies was assessed by measuring the % of mobile flies; flies were not allowed to contact the AITC source. Increasing the concentration of AITC decreases the % of mobile flies, likely due to intoxication. ****p<0.0001, log-rank Mantel-Cox tests against the control. **(B)** Schematic and representative trace of single sensillum recording (SSR) from pb1 OSNs upon stimulation with AITC 1:100 vol/vol. The horizontal bar indicates the onset of the stimulus and its duration (1 sec). **(C)** Single sensillum recordings (SSR) from all *Dmel* antennae and palp basiconic sensilla upon AITC stimulation (1:100 vol/vol; n= 6-10 recordings/sensilla type from 6 animals). Represented here and in all the figures are the control-subtracted net number of spikes/sec, unless otherwise noted. The horizontal dotted line at zero indicates no response to odor stimulation. Here and thereafter, horizontal bars represent the median, the edges of the boxes correspond to 25th and 75th quartiles, the whiskers denote 10th and 90th quartiles, and symbols indicate outliers. **(D)** Responses from pb1 sensilla of Canton-S, *w*^*1118*^, *Or42a−/−*, and *TrpA1*^*−*^ *Dmel* flies to 1:100 vol/vol AITC stimulation (n=6-10 sensilla/genotype from 3-4 animals/genotype). The responses of both mutant flies were compared against each other and against those of w1118. Kruskal-Wallis ANOVA followed by Dunn’s multiple comparisons (****p<0.001, **p<0.01, *p<0.05). **(E)** Food consumption of genetic control flies (n=16) and Or42a mutants (n=14) in presence or absence of a non-toxic concentration of volatile AITC (1:500 vol/vol). Both control flies and mutants fed less in the presence of AITC volatiles (one-sample signed rank tests; respectively p<0.05 and p<0.001), but the feeding score of genetic control flies was lower than that of mutant flies (Mann-Whitney U test, ***p<0.001). The dotted line at 50% indicates no feeding aversion or enhancement. **(F)** Positional olfactory assay. Flies could smell, but not contact, the AITC solution (Figure Supplement 1C). The number of flies in the odorless and the odorous glass tubes was counted every five 5 minutes until minute 35, and then again at 65 minutes. The dotted line at 50% indicates random distribution between the two tubes. Genetic control flies (blue, n=15) avoided the tube closest to the odor source at various time points (***p<0.005, ****p<0.001; one-sample signed rank tests against median=50%). *Or42a−/−* mutants (orange, n =15) distributed randomly between the two tubes at all timepoints (p>0.05) except at 65 minutes (**p<0.01).

We conducted mobility assays as a proxy for toxicity with various concentrations of volatile AITC (from 1:500 to 1:2.5 vol/vol). In our experimental setup, a fabric mesh separated the chamber containing the flies from the chamber containing the AITC solution (Figure Supplement 1A) and therefore, flies were exposed to volatile AITC but could not contact (i.e. taste) the AITC solution directly. While all animals in both the control treatment and those exposed to AITC 1:250 and 1:500 vol/vol remained active, most flies exposed to AITC concentrations ≥ 1:50 vol/vol became paralyzed (likely highly intoxicated or even dead) within 10 minutes (Figure 1A). Thus, this rapid immobilization indicates that plant-derived electrophilic compounds like AITC can have a strong negative effect on flies, consistent with previous findings using volatile PITC (Lichtenstein et al. 1964).

### Volatile AITC is detected by Or42a in *Dmel*

The high toxicity caused by volatile AITC indicates that this volatile had the potential to be detected by the fly’s olfactory system, and that this could provide a fitness benefit if these volatile toxins were then behaviorally avoided. To investigate this, we conducted exhaustive single sensillum recordings (SSR) from the fly’s olfactory organs, the antennae and the maxillary palps, upon stimulation with volatile AITC. Several OSNs showed excitatory responses to AITC, but OSNs in palp basiconic (pb) sensilla (pb1a) were the most strongly activated (Figure 1 B–C, >100 spikes/sec). Because previous studies reported that *Dmel* pb1a OSNs express Or42a (Couto et al. 2005), we investigated whether AITC responses in pb1a OSNs are indeed mediated by Or42a. OSNs in pb1 sensilla from control flies showed strong responses to volatile AITC, while OSNs in pb1 sensilla from *Or42a* null mutant flies showed no response (Figure 1D, <2 spikes/second), indicating that Or42a detects volatile AITC in these sensilla. Furthermore, OSNs in pb1a of *TrpA1* null mutant flies responded strongly to AITC (>150 spikes/second, Figure 1D), showing that the contact chemoreceptor TrpA1 is not necessary for maxillary palp olfactory detection of this compound. Or42a is the first Or reported to detect an ITC in *Dmel* to our knowledge.

### Volatile AITC repels *Dmel* via Or42a

Next, we examined if activation of OSNs housing Or42a plays a role at the behavioral level (whole organism). Previous studies have shown that the presence of food-related odorants increases sugar consumption in *Dmel* (Reisenman and Scott, 2019). Based on this, we hypothesized that the presence of aversive odorants such as AITC would decrease sugar consumption. We conducted consumption assays in which groups of 10-15 food-deprived female flies were offered 50 mM glucose water solution dyed blue in the presence or absence of volatile AITC (1:500 vol/vol loaded on filter paper), such as that flies could smell, but not contact, the AITC solution (Figure Supplement 1B). After 15 minutes flies were frozen and then a single feeding score/vial was calculated based on the amount of blue dye in the abdomen of all flies within a vial. We found that genetic background control flies fed less when volatile AITC was present than in the presence of the solvent control (one-sample signed rank tests on normalized data, p<0.001, n=16; Figure 1E); wild-type Canton-S flies also fed less in presence than in absence of volatile AITC (p<0.05, n=23; not shown). Mutant flies fed less in the presence of AITC volatiles (p<0.05) as well, but their feeding scores were higher than that of the genetic control group (p<0.001, Mann-Whitney U test; Figure 1E). These results indicate that Or42a mediates aversion to AITC in this behavioral context.

Additionally, we conducted a positional olfactory assay based on Ohashi and Sakai (2015), with various modifications to prevent flies from physically contacting the odor source (Figure Supplement 1C). Groups of 10-12 female flies were released at the juncture between the two glass pipettes and were allowed to choose between the pipette closest to the odor source (“odorous side/tube”) and the pipette closest to the solvent (“control side/odorless tube”; Figure Supplement 1C). The number of flies in each tube as well as in the release section was counted every five minutes up to minute 35, and then again at 65 minutes. We first confirmed that flies can show normal olfactory-guided behavior in this behavioral set-up using apple cider vinegar, a well-established olfactory attractant (Semmelhack and Wang 2009; Becher et al. 2010). Canton-S flies preferred the odorous tube from minute 25 onwards (one-sample signed rank tests, p<0.05 in all cases, n=21, Supplementary File 5). We then tested whether AITC causes olfactory repellence in this behavioral context. Genetic control flies and wild-type Canton-S flies avoided the AITC tube at various time points (25, 30, 35 and 65 minutes in genetic controls, Figure 1F; and from 25 minutes onwards in Canton-S, Figure Supplement 2B). In contrast, *Or42a*−/− mutants avoided the AITC tube only at the 65 minutes time point (Figure 1F), possibly by taste detection of AITC via TrpA1 (we cannot exclude the possibility that volatile AITC molecules adhered to the glass tube walls at this point). These findings suggest that *Or42a* plays a crucial role in mediating olfactory-driven behavioral aversion to AITC. Similar percentages of genetic control and *Or42a*−/− mutants choose one or the other tube at all time points (Figure Supplement 2D), indicating that *Or42a*−/− mutants are as active as wild-type flies in the presence of AITC; Canton-S flies also remained similarly active in presence of AITC volatiles (Figure Supplement 2C). We also confirmed that *Or42a*−/− mutants are capable of odor-mediated olfactory orientation. We conducted dual-choice trap assays offering apple cider vinegar vs. water as described in Matsunaga et al. (2021). Approximately 50% (median) of flies released in each test (N=8 tests, n=20 females/test) were trapped and of these, 100% (median) were found in the odor-baited trap (Wilcoxon-matched pairs test, p<0.005; Supplementary File 5). This result confirms that the lack of behavioral aversion to AITC in *Or42a*−/− was not due to a generalized olfactory defect in this line.

It has been reported that Or42a OSNs respond to a variety of fruit/fermentation volatiles, including γ-hexalactone, and that some of these activators can furthermore mediate behavioral attraction (Dweck et al. 2016). We thus used γ-hexalactone to test whether our behavioral assays yielded results consistent with these previous findings. In consumption assays (duration=10 minutes) Canton-S flies increased their feeding in the presence of volatile γ-hexalactone 1:50 vol/vol, but this effect was lost in *Or42a*−/− mutants (Figure Supplement 2A). In the positional olfactory assay, a larger proportion of genetic control flies than of *Or42a*−/− mutants chose either tube in presence of 1:10 vol/vol γ-hexalactone at all time points (Figure Supplement 2F), suggesting that the presence of the odor increases exploratory activity in control flies. Moreover, control flies showed attraction to 1:10 vol/vol γ-hexalactone (at 20 and 30 minutes, one-sample signed rank tests, p<0.05 in both cases), but this attraction was lost in *Or42a*−/− mutants (Figure Supplement 2E). Canton-S flies also showed attraction to this odor (at 5, 10, and 15 minutes, n=12; p<0.05 at all those three times, Supplementary File 5). Furthermore, we found that volatile γ-hexalactone does not immobilize flies (Figure Supplement 2K). Thus, our behavioral assays show that volatile γ-hexalactone attracts flies and is harmless, in line with previous results (Dweck et al., 2016).

Given that Or42a mediates both repellence to AITC and attraction to γ-hexalactone (Figure 1E–F and Figure Supplement 2A and 2E–F), we next tested whether these two odorants activate additional OSNs. Through exhaustive SSR from antennae and maxillary palps upon stimulation with γ-hexalactone, we found that pb1a OSNs were the only OSNs activated by γ-hexalactone in basiconic, intermediate, and trichoid sensilla (Figure Supplement 2L). Due to technical limitations, we were unable to conduct SSR from antennal coeloconic sensilla, but Yao et al (Yao et al. 2005) reported that γ-hexalactone activates OSNs in ac3b, which house Or35a OSNs (Oh et al. 2021). In contrast, AITC activated several OSNs within the above mentioned sensilla types, including those in ab4a (Figure 1C). Thus, we assessed whether *Or7a* and *Or35a* respectively contribute to repellence/attraction in olfactory positional assays. Compared to genetic controls, *Or7a* −/− flies showed reduced aversion to AITC (Figure Supplement 2G–H), while *Or35a*−/− flies lost the attraction to γ-hexalactone (Figure Supplement 2I–J). These results suggest that the activation of Or42a OSNs mediates aversion to AITC in conjunction with Or7a OSNs, but can also mediate attraction to γ-hexalactone if active in conjunction with Or35a OSNs. Thus, our findings support a combinatorial hypothesis of odor coding with respect to these ligands and indicate that the valence of Or42a-mediated behavioral responses is context-dependent (Figure Supplement 2M).

### Pb1a-like OSNs in Brassicales-specialists evolved broadened sensitivity to isothiocyanates (ITCs)

We next investigated how the evolutionary transitions from microbe-feeding to herbivory have affected OSNs, using the reference species *Dmel* as well as species within *Scaptomyza*: the microbivorous *S. hsui* (hereafter: *Shsu*) and *S. pallida* (hereafter: *Spal*), and the herbivorous Brassicales specialists *Sfla* and *Smon* (Kim et al. 2021; Peláez et al. 2023). We investigated whether *Scaptomyza* species have pb1-like sensilla homologous to *Dmel* pb1 and if so, the extent to which they respond to a broader range of ITC compounds, since Brassicales plants release ITCs upon wounding (MacLeod et al. 1989; Cuellar-Nuñez et al. 2022).

**Figure 2:**
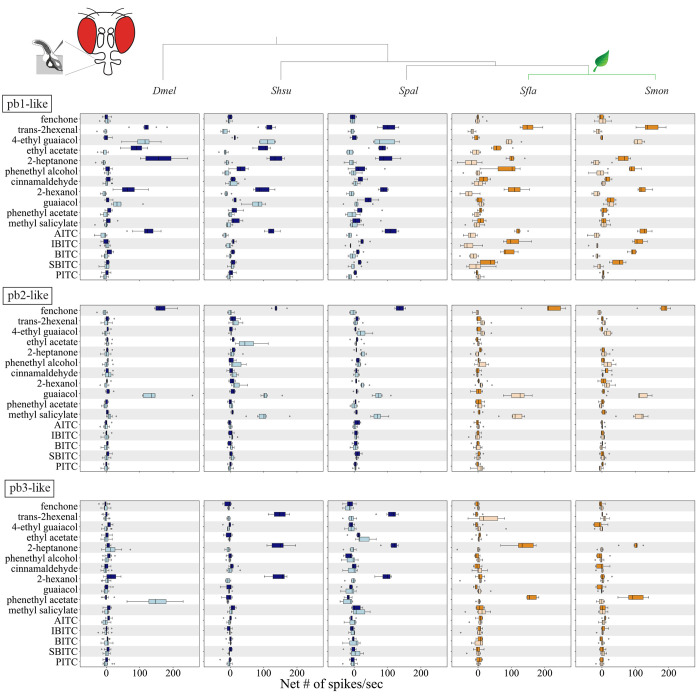
Functional characterization of maxillary palp OSNs in *Dmel* and *Scaptomyza* species. Single sensillum recordings (SSRs) from maxillary palp OSNs of *Dmel, Shsu, Spal, Sfla*, and *Smon*. Stimuli (1:100 vol/vol) included diagnostic chemicals used to identify Ors in *Dmel* (see Methods), fruit volatiles, green leaf volatiles (GLVs), and Brassicales plant-derived isothiocyanates (ITCs) (n=6-9 from 3-4 animals/species). All sensilla housed two OSNs, labeled “a” (darker color) and “b” (lighter color). Their response profiles and stereotyped locations within the palp support the classification of *Scaptomyza* sensilla into three types (Figure 3, Figure Supplement 5): pb1-like, pb2-like, and pb3-like. While pb1a sensilla from all species responded to AITC, pb1a-like from *Sfla* and *Smon* additionally responded to other ITC compounds. Mustard specialization occurred at the clade leading to the common ancestor of these two species, denoted by the leaf cartoon. AITC: allyl ITC, IBITC: isobutyl ITC, BITC: butyl ITC, SBITC: sec-butyl ITC, PITC: phenethyl ITC.

We first validated our methods for functional characterization of sensilla in *Dmel*. We used compounds that serve as diagnostic for the three sensilla types found in the maxillary palps of *Dmel* (see methods for details). In addition, we used as stimuli *trans*-2-hexenal, 2-hexanol, methyl salicylate, 2-heptanone and phenethyl alcohol, and Brassicales-derived isothiocyanates (ITCs) including allyl ITC (AITC), isobutyl ITC (IBITC), butyl ITC (BITC), sec-butyl ITC (SBITC), and phenethyl ITC (PITC). Using the diagnostic odorants, we confirmed the presence of three different types of palp sensilla (pb1, pb2 and pb3) in *Dmel*. We also found that *Dmel* pb1a OSNs responded to AITC (>120 spikes/sec), but not to the other ITCs tested (<10 spikes/sec, Figure 2).

**Figure 3:**
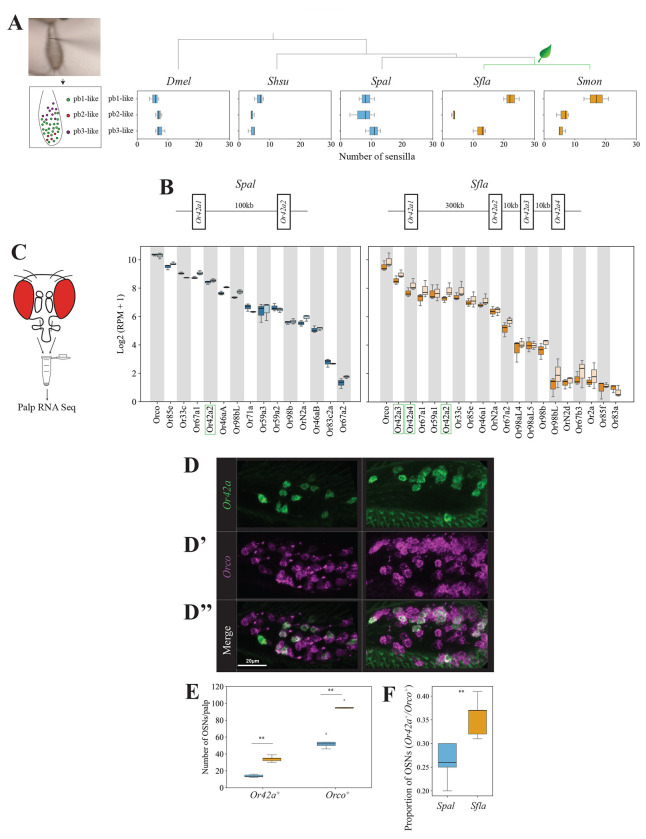
High expression of *Or42a* paralogs and over-representation of *Or42a*-positive-OSNs in the maxillary palp of *S. flava*. **(A)** Picture of SSR from *Sfla* maxillary palp sensilla (top left) and a representative anatomical mapping of *Sfla* palp sensilla (bottom left) obtained using diagnostic chemicals. Green: pb1-like, red: pb2-like, and magenta: pb3-like. Boxplots in the graph (right) represent the number of each sensilla type in *Dmel* and the four *Scaptomyza* species (n=3 animals/species; see Figure Supplement 5 for individual maps). Maxillary palp pb1 sensilla are over-represented in the two mustard specialists. **(B)** *Or42a* syntenic regions in the genomes of *Spal* and *Sfla*, with a gene triplication in the *Sfla* genome at the syntenic region of *Spal Or42a2* (*Sfla Or42a2*, *Sfla Or42a3*, and *Sfla Or42a4*). **(C)** Maxillary palp RNA seq of *Spal* and *Sfla Or*s (n=3 replicates/sex and species). *Or*s with median values of the log2 (RPM +1) < 1 (n=3) were excluded. **(D-D”)** Representative images of HCR RNA FISH from the maxillary palps of *Spal* and *Sfla* showing *Or42a*-positive-OSNs (**D**, green), *Orco*-positive-OSNs (**D’**, magenta**),** and the merged signals (**D”**, white indicates co-localization of *Or42a*-positive-OSNs and *Orco*-positive-OSNs). Scale bar: 20μm. **(E)** Number of *Or42a*-positive-OSNs and *Orco*-positive-OSNs in one maxillary palp of *Spal* (blue boxes) and *Sfla* (orange boxes, left panel) and ratio between them (right panel). Mann-Whitney U tests, ** p<0.01; n=5 animals/species.

We next characterized OSNs in pb sensilla in the four *Scaptomyza* species and used the spike rate to determine if data clustered by sensilla type (Figure 2, Figure Supplement 3A). In all species, sensilla fell into three functional classes akin to *Dmel* pb1, pb2 and pb3, which we termed pb1-like, pb2-like, and pb3-like. The response profiles of *Scaptomyza* pb3-like OSNs were different from those of *Dmel* pb3, likely because the homologs of *Or59c* and *Or85d*, which are respectively expressed in *Dmel* pb3a and p3b, are unidentified in the genomes of *Scaptomyza* (Goldman-Huertas et al. 2015). We found two types of pb1-like sensilla across the four *Scaptomyza* species. Both OSNs in pb1a exhibited an odorant tuning pattern similar to that of *Dmel* pb1a. On the other hand, one subset of pb1b-like OSNs exhibited an odorant tuning pattern akin to that of *Dmel* pb1b, while the other subset of pb1b-like OSNs consistently showed very small spike amplitudes and thus was excluded from the analysis. As in *Dmel*, AITC activated pb1a-like sensilla in all four *Scaptomyza* species (Figure 2). Notably, several other ITCs, including IBITC, BITC, and SBITC, additionally activated *Sfla* and *Smon* pb1a-like OSNs (Figure 2, first row). Methyl salicylate activated all *Scaptomyza* pb2b-like sensilla but not *Dmel* pb2b (Figure 2, second row) and interestingly, this compound is a known *Sfla* volatile attractant in natural settings (Orre et al. 2010). Furthermore, the odorant response profiles of OSNs in pb3-like sensilla were more similar between the more distantly related species *Shsu* and *Spal*, than between the more closely related species *Spal* and *Sfla*, or *Spal* and *Smon* (Figure 3, third row). For example, OSNs in pb3a-like sensilla were activated by *trans*-2-hexenal in *Shsu* and *Spal* OSNs (but not in *Sfla* or *Smon*), while *Sfla* and *Smon* OSNs (but not *Shsu* or *Spal*) were activated by phenethyl acetate (Figure Supplement 3B–C).

We also conducted SSR from pb1a (or pb1a-like) sensilla of the microbe-feeding species (*Dmel, Shsu* and *Spal*) and the Brassicales specialists (*Sfla* and *Smon*) upon stimulation with various concentrations of odorants from 10^−5^ to 10^−2^ vol/vol. For the most part, for all species and odorants, responses increased with increasing concentration (Figure Supplement 4A). For comparing odor sensitivity across species, we calculated the odorant concentration required to elicit a biological response halfway between the baseline and the maximum (50% effective concentration values, EC_50_; Figure Supplement 4D). Interestingly, the spiking responses to γ-hexalactone stimulation were lower in *Sfla* compared to *Shsu*, and correspondingly, the EC_50_ was higher in the former (note that *Sfla* had even lower sensitivity to γ-hexalactone than *Smon*; Figure Supplement 4B Figure Supplement 4B and D). The responses of pb1a-like sensilla to *trans*-2-hexenal were higher in *Sfla* than in *Smon* (Figure Supplement 4C), and their EC_50_s were lower in comparison to those of *Dmel* or *Shsu* (Figure Supplement 4D). Similarly, the EC_50_s to AITC were lower in *Sfla, Smon*, and *Spal* than in *Dmel* (Figure Supplement 4D), indicating higher sensitivity to this volatile compound in those three *Scaptomyza* species. Finally, *Sfla* and *Smon* showed similar EC_50_s to all the four ITCs tested (Figure Supplement 4D). Overall, all these findings suggest that microbe-feeding species exhibit higher γ-hexalactone sensitivity, whereas Brassicales specialists show heightened sensitivity to *trans*-2-hexenal and ITCs in general. In particular, the heightened sensitivity of *Sfla* OSNs to the general leaf odor *trans*-2-hexenal in comparison with that of *Smon* OSNs aligns with its potentially broader host range (Martin 2004; Maca 1972).

### Brassicales specialists have a higher number of pb1-like sensilla.

We next generated anatomical maps of sensilla on the anterior part of the maxillary palps of the species of *Scaptomyza* (and of *Dmel* for comparison) using diagnostic chemicals (Figure 3A, Figure Supplement 5, Supplementary file 10; see methods for more detail). Interestingly, while *Dmel* had a relatively randomized distribution of sensilla on the palps, consistent with previous reports (de Bruyne et al. 1999), all four *Scaptomyza* species exhibited a more organized sensilla pattern, with pb1-like, pb2-like, and pb3-like respectively located medially, distally, and proximally as reported in *D. mojavensis* (Crowley-Gall et al. 2016).

The proportions of each type of palp sensilla varied within and between species (Figure 3A and Figure Supplement 5). Both *Sfla* and *Smon* have a larger number of pb1-like than of pb2-like or pb3-like sensilla. In contrast, *Dmel* and the other two *Scaptomyza* species had similar numbers of each sensilla type. These findings suggest an expansion of pb1-like sensilla in Brassicales specialists over the course of evolution. Moreover, we observed a trend in which *Sfla* and *Smon* had an overall larger number of palp olfactory sensilla compared to *Dmel*, *Shsu* and *Spal* (Figure 3A, Figure Supplement 5, Supplementary file 11). Within *Scaptomyza*, the larger number of sensilla in *Sfla* concurred with this species’ larger palp surface area (Figure Supplement 6). Taken together, these results show that Brassicales plant specialists tend to have a higher proportion of pb1-like sensilla compared to microbe-feeding *Drosophila* and *Scaptomyza*, suggesting that Brassicales plant specialist *Scaptomyza* species may have enhanced capacity to detect volatile ITCs.

### *Sfla Or42a* is triplicated in the genome of *Sfla* and is highly expressed in the maxillary palps.

We next investigated the expression of *Or* genes in maxillary palps OSNs across some of the species of interest. Initially, we scanned the known genome sequences of species in the genus *Scaptomyza* and found a duplication of *Or42a* in the lineage leading to all known *Scaptomyza*, while the *Dmel* outgroup had a single *Or42a* homolog (Figure Supplement 7). Interestingly, within *Scaptomyza*, *Sfla* exhibited serial triplicates in one of the *Or42a* duplicates, which we named *Or42a2*, *Or42a3*, and *Or42a4* (Figure 3B and Figure Supplement 7), whereas *Smon* retained only two paralogs, like *Shsu* and *Spal*.

To determine the extent of *Or* gene expression, including that of the *Or42a*s paralogs of *Scaptomyza*, we conducted species- and sex-specific RNA transcriptome analyses of *Spal* and *Sfla* maxillary palps (Figure 3C; Figure Supplement 8). We confirmed the expression of *Spal Or42a* and *Sfla Or42a2-4* in these organs. Interestingly, the *Sfla Or42a* paralogs were each expressed at levels comparable to those of other *Or* genes, such as *Or33c*, the homolog of which in *Dmel* is expressed in OSNs of pb2a sensilla (see below). Furthermore, we confirmed the expression of homologs *Or71a* (expressed in OSNs of *Dmel* pb1b), and of *Or33c/Or85e* and *Or46a* (expressed in OSNs of *Dmel* pb2a and pb2b) in both *Spal* and *Sfla* maxillary palps. *Spal Or42a2* and *Sfla Or42a2-4* were expressed in the OSNs of their respective palp sensilla (pb1-like), whereas *Spal Or42a1* and *Sfla Or42a1* were not. *Or85d* and *Or59c* were respectively expressed in OSNs of *Dmel* pb3a and pb3b but the homologs were unidentified in the genomes of *Scaptomyza* (Goldman-Huertas et al., 2015). Instead, *Or59a1, Or67a1, Or67a2*, and *OrN2a* were strongly expressed in OSNs of both *Spal* and *Sfla*, suggesting that these homologs may be expressed in pb3a or pb3b (Figure 3C). In summary, the potential conservation of *Or* expression in pb1 and in pb2 sensilla, and the difference in *Or* expression in OSNs of pb3 between *Dmel* and *Sfla*, may reflect the similarities and differences in their odorant response profiles (See Figure 2).

### The Brassicales specialist *Sfla* have more *Or42a*-positive-OSNs.

Our transcriptome analysis revealed high expression of *Or42a* triplicates in *Sfla* maxillary palps (Figure 3C), which could be due to high expression of *Or42a* in individual cells and/or a higher number of *Or42a*-positive-OSNs. To distinguish between these possibilities, we quantified the number of *Or42a*-positive-OSNs in the maxillary palps of *Sfla* and *Spal* using hybridization chain reaction RNA fluorescent in situ hybridization (HCR RNA FISH). Due to the high sequence similarity between the paralogs, we designed an RNA probe based on the conserved sequence region of *Sfla Or42a2-4* to visualize all *Or42a2-4* paralog^+^ cells in *Sfla*. Similarly, we designed the *Spal Or42a2* probe to compare the expression of homologs in the maxillary palps of *Sfla* and *Spal* (Figure 3B).

HCR RNA FISH analysis revealed that the maxillary palps of *Sfla* contained more *Or42a*-positive-OSNs than those of *Spal*, as well as more *Orco*-positive-OSNs (Figure 3D–E). Because all maxillary palp OSNs express Orco (Larsson et al. 2004), the number of Orco-positive OSNs represents the total number of palp OSNs. The ratio of the total number of *Or42a*-positive-OSNs to the total number of *Orco*-positive-OSNs was higher in *Sfla* (Figure 3F). This indicates that *Sfla* not only has more *Or42a*-positive-OSNs and *Orco*-positive-OSNs, but also a greater proportion of *Or42a*-positive-OSNs relative to the overall number of OSNs in the maxillary palps.

### Paralog-specific functional evolution of Or42a.

**Figure 4:**
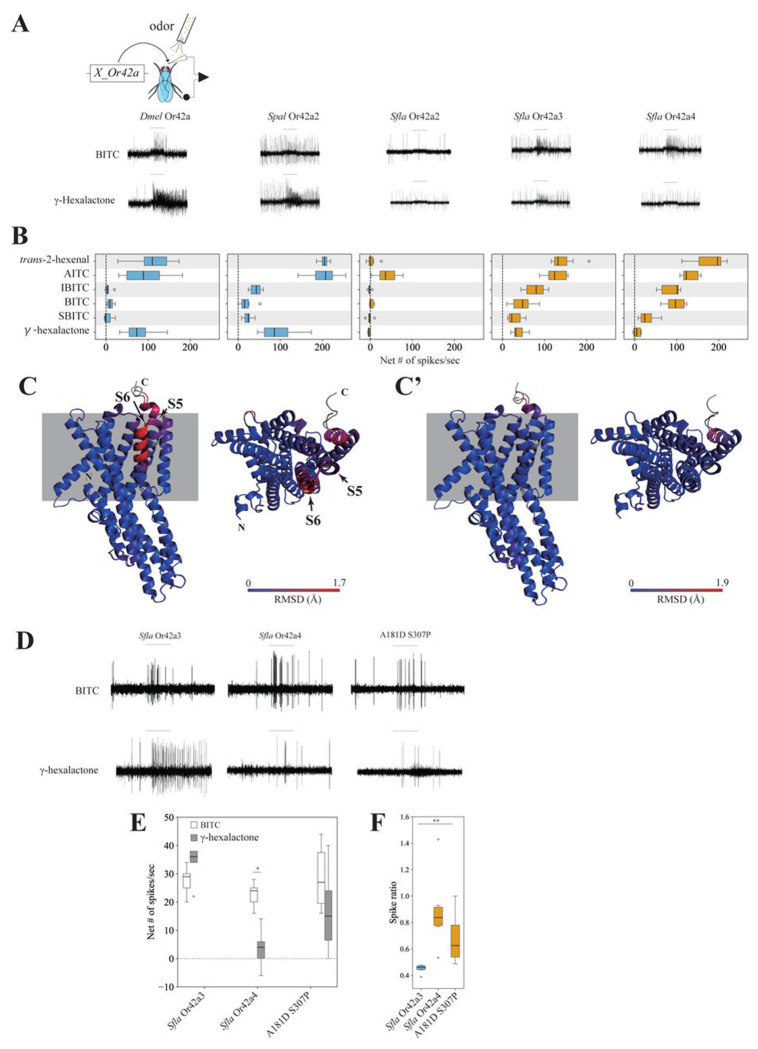
Functional characterization of chimeric Or42a (A181D and S301P) and Or42a from *Dmel, Spal*, and *Sfla* **(A)** Representative SSR traces from *Dmel* at1 sensilla OSNs expressing species-specific *Or42a* under the control of *Or67d* ^*Gal4*^ (fly genotype: *UAS-Or42a; Or67d* ^*Gal4*^) in response to stimulation with BITC and the fruit odor γ-hexalactone. **(B)** Responses of at1 OSNs (n=6-8 sensilla from 3-4 animals/genotype) expressing species-specific *Or42a* upon stimulation with *trans*-2-hexenal (a general leaf odor released upon leaf mechanical damage such as crushing), various ITCs produced by mustard plants (AITC, IBITC, BITC, SBITC), and γ-hexalactone. **(C-C’)** The 3D alignment of *Sfla* Or42a3 and *Sfla* Or42a4 predicted by AlphaFold2 **(C)**, and 3D alignment of *Sfla* Or42a4 and a chimeric Or42a with two amino acid substitutions (A181D and S301P) in the background of *Sfla* Or42a3 **(C’)**. The root mean square deviation (RMSD) is visualized with a color gradient from blue (low) to red (high) in both the side (left) and the top view (right). The upper and lower sections of the side view represent the extracellular and the intracellular regions, respectively, with the cell membrane (gray rectangles) separating them. **(D-F)** Representative SSR from *Dmel* at1 sensilla expressing heterologous *Sfla* Or42a3, Or42a4, and the chimera (genotypes of *UAS-Or42a/CyO; Or67d* ^*Gal4*^) upon stimulation with BITC and γ-hexalactone (**D**), at1 population responses to BITC (white bars) and to γ-hexalactone (gray bars) (**E**, n=6-10 sensilla from 3-4 animals/genotype; * p<0.05, Mann-Whitney U test), and response ratio [**F**, (response to BITC) / (response to BITC + response to γ-hexalactone); **p<0.01, ***p<0.001, Kruskal-Wallis ANOVA followed by Dunn’s multiple comparisons].

We next investigated the functional evolution of *Or42a* triplicates in *Sfla*. To do this, we expressed *Dmel Or42a, Spal Or42a2*, and *Sfla Or42a2-4* in *Dmel* antennal trichoid 1 (at1) OSNs in the background of a null mutation for *Or67d*, the at1 cognate receptor (Kurtovic et al. 2007). We selected at1 because some insects utilize host-derived chemicals as pheromones (Reddy and Guerrero 2004), and certain pheromones only activate OSNs housed in trichoid sensilla in *Dmel* (Xu et al., 2005 Neuron; Benton et al., 2007). We conducted SSR from at1 using six different odorant stimuli at 1:100 vol/vol. We found that γ-hexalactone, *trans*-2-hexenal, and AITC strongly activated OSNs expressing *Dmel* Or42a (>79 spikes/second) or *Spal* Or42a2 (>96 spikes/sec; Figure 4A–B), while IBITC, BITC and SBITC evoked much weaker responses from these two paralogs (respectively <11 and 18 spikes/second). In contrast, OSNs expressing *Sfla* Or42a3 or *Sfla* Or42a4 were very sensitive to IBITC and BITC (> 87 and 46 spikes/second, respectively), which is consistent with the response profiles of *Sfla* pb1a OSNs (Figure 2). Notably, OSNs expressing *Sfla* Or42a4 showed only small responses to γ-hexalactone, and *Sfla* Or42a2 was only activated by AITC (Figure 4B). These results align with the niche difference between microbe-feeding flies and Brassicales specialists. Moreover, our findings reveal the paralog-specific functional evolution of Or42a triplicates in *Sfla*, as different paralogs have evolved distinct sensitivities to various ITC chemicals and fruit odors.

### AlphaFold2-led screening with ectopic expression of *Sfla* Or42a reveals the molecular changes underlying changes in odor sensitivity

We investigated which amino acid substitutions in *Sfla* O42a3 lead to the gain of sensitivity to BITC and the decreased sensitivity to γ-hexalactone in *Sfla* Or42a4 (there are total of 32 amino acid differences between *Sfla* Or42a3 and *Sfla* Or42a4, Figure Supplement 9A). For this, we predicted the 3D structures of *Sfla* Or42a3 and *Sfla* Or42a4 in the cell membrane and aligned them in 3D space using PyMol, and checked the overlap between Ors (Jumper et al. 2021; Mirdita et al. 2022; Benton and Himmel 2023; Himmel et al. 2023).

We first confirmed that the predicted Local Distance Difference Test (pLDDT) scores for the *Sfla* Or42a3 and *Sfla* Or42a4 structures were sufficiently high to ensure confidence in the 3D predictions in general, except for the N-terminal and C-terminal regions (Figure Supplement 9B). One of the most striking differences in 3D structures between *Sfla* Or42a3 and *Sfla* Or42a4 was observed in the S5 and S6 helices in the transmembrane region (~1.7Å root mean square deviation, RMSD). These helices are reported to contain the ligand binding pockets (Figure 4C–C’ and Figure Supplement 9B; Del Mármol et al. 2021; Wang et al. 2024; Zhao et al. 2024). Importantly, we confirmed that the pLDDT scores for these non-overlapping sites were above 70, indicating that the 3D predictions of these sites are reliable (Figure Supplement 9B). We then substituted each of the 32 amino acids in *Sfla Or42a4* individually with the corresponding residues from *Sfla* Or42a3 *in silico*, predicted the 3D structures of the chimeras, and aligned them with *Sfla* Or42a4 in 3D space until the local structural differences were resolved. Remarkably, the substitutions of A181D and S307P in *Sfla* Or42a3 (hereafter referred to as A181D S307P) reduced the RMSD to approximately 0.1 Å in the S5 and S6 helices when aligned with *Sfla* Or42a4, indicating that these two mutations effectively resolved the local structural differences (Figure 4C–C’ and Figure Supplement 9B).

We then used the *Dmel* at1 empty neuron system to investigate whether these two amino acid substitutions could account for the *Sfla* Or42a4 enhanced sensitivity to BITC and reduced sensitivity to γ-hexalactone, in comparison with *Sfla* Or42a3. While the A181DS307P variant and the two *Sfla* paralogs show similarly moderate responses to BITC (Figure 4E), the BITC to γ-hexalactone response ratios of *Sfla* Or42a4 and A181D S307P were not different from each other, but were both higher than that of *Sfla* Or42a3 (Figure 4F). These findings suggest that the two amino acid substitutions are sufficient to increase the response to BITC relative to that of γ-hexalactone in *Sfla* Or42a4. This effect was observed in flies carrying the heterozygous genotype A181D S307P/+ but not in flies with the homozygous A181D S307P genotype (Compare Figure 4E–F with Figure Supplement 11), possibly due to saturation effects. In summary, our findings demonstrate that the A181D and S307P substitutions are critical for shifting the receptor’s sensitivity from fruit odorants to ITCs.

## DISCUSSION

In this study, we first discovered that plant-derived ITCs have a detrimental effect on survival of *Drosophila melanogaster* (*Dmel*) through volatile exposure. We then found that these toxic electrophilic volatiles are detected by Or42a, which is expressed in the maxillary palps. This Or is necessary for behavioral aversion and to our knowledge, is the first known ITC-detecting olfactory detector in *Dmel* and plays a role in mediating behavioral aversion to ITCs. Thus, in *Dmel*, Or42a works in combination with the “wasabi” taste receptor” TrpA1 and Painless (Al-Anzi et al. 2006; Kang et al. 2010; Mandel et al. 2018), and possibly with other Ors, to facilitate adaptive behavioral avoidance of these chemicals through both gustatory and as we show here, olfactory chemosensory modalities.

We also conducted molecular evolutionary analyses, site-directed mutagenesis, and functional assays to uncover the molecular mechanisms by which the Brassicales specialist *Sfla* evolved paralogous copies of *Or42a* genes that responded to a broad range of electrophilic compounds emitted by their host plants. Overall, our findings provide new insight into the olfactory mechanisms that allow generalist insects to avoid lethal volatile toxins, and into the rapid evolution of an expanded set of more finely tuned *Or*s in specialist insects that detect these volatile host-plant signals.

### Plant-derived volatile AITC is toxic to *Dmel* and its detection and avoidance is mediated by *Or42a*-positive-OSNs

Plants have evolved a diverse array of specialized metabolites that repel or intoxicate insects (Ibanez et al. 2012). Among these, plant-derived electrophilic compounds, including *trans*-2-hexenal and ITCs, are highly reactive (Noge and Becerra 2015; Tocmo et al. 2021). For example, AITC derived from wasabi and other mustard and Brassicales plants causes a highly pungent sensation by activating contact-mediated pain receptor TrpA1 of humans (Bell et al. 2018). Here, we found that volatile AITC exerts a dose-dependent detrimental effect on *Dmel* adults (Figure 1A), underscoring the high toxicity of this compound. These findings are consistent with previous findings showing that ingestion of AITC decreases fly survival (Zimmermann et al. 2024) and with earlier studies on the toxic effect of volatile PITC (Lichtenstein et al. 1964).

TrpA1 pain receptors in *Dmel* mediate the detection of ITCs and other reactive compounds by taste (i.e, by contact), as they do in humans (Kim et al. 2010). In addition, the OSNs of some mustard-plant specialists, such as the diamondback moth *Plutella xylostella* (via Or35a and Or49a; Liu et al. 2020) and *Sfla* (Matsunaga et al. 2021) also detect these compounds. However, it was unclear whether non-specialists, such as *Dmel* and humans, have evolved strategies to detect these compounds via olfaction. We found that Or42a and its associated OSNs are necessary for olfactory responses in *Dmel* pb1a sensilla (Figure 1B–D). Furthermore, this Or was necessary for inducing olfactory aversion to AITC in *Dmel* in two different behavioral contexts (Figure 1E–F and Figure Supplement 2B). It would be interesting to investigate whether vertebrate also possess volatile electrophile sensors, despite the different evolutionary origins from insect chemoreceptors.

Our results indicate that Or42a OSNs, in combination with Or7a and Or35a, contribute to mediate olfactory repellence to AITC and attraction to the fruit volatile γ-hexalactone (Figure 1E–F and Figure Supplement 2B–J). How does a single OSN type (*Or42a*-positive-OSNs) mediate aversion to AITC while also driving attraction to γ-hexalactone? We found that AITC also activates (to a lesser degree) OSNs in ab4a sensilla (Figure 1C), which expresses Or7a, a reported “generalist Or” that also responds to aversive odorants (Münch and Galizia 2016). Additionally, γ-hexalactone activates Or35a-expressing OSNs in the ac3B sensilla, mediating attraction to yeast odors (Yao et al., 2005; Oh et al., 2021). Consistently with these results and previous reports, we found that *Or7a* −/− flies also avoided AITC but less so than genetic controls (Figure Supplement 2G–H), while *Or 35a* −/− lost the attraction to γ-hexalactone (Figure Supplement 2I–J). These findings suggest that the valence of the behavioral responses mediated by Or42a is context dependent: Or42a mediates aversion when another aversive olfactory circuit (e.g. Or7a) is active, whereas it mediates attraction when an additional attractive olfactory channel is active (e.g. Or35a; Figure Supplement 2M). Future investigations at the circuit level, particularly on the role of interglomerular interaction via local neurons (Haverkamp et al. 2018), will be essential to further elucidate the neural mechanisms mediating these behavioral responses of opposite valence.

### Functional evolution of *Or42a* and *Or67b* in *Scaptomyza* mustard plant specialists

Insect specialization on chemically defended host plants provides a novel niche for host use if they can overcome the initial toxic barrier (Ali and Agrawal, 2012). Indeed, specialized insects often use these toxic compounds as olfactory “tokens” to find their host plants. Many Brassicales plants release species-specific volatile ITCs (Wu et al. 2021) at particular ratios and concentrations that attract a guild of specialized herbivores through olfaction. For instance, *P. xylostella* uses volatile ITCs to find suitable oviposition sites (Liu et al. 2020), volatile ITCs attract *Sfla* females at least in the laboratory (Matsunaga et al., 2021), and herbivorous *Scaptomyza* species have lost four *Or*s that in *Dmel* mediate attraction to yeast-associated volatiles (Goldman-Huertas et al. 2015; Matsunaga et al. 2021). Interestingly, all *Scaptomyza* also lost *Or7a* (which is expressed in ab4), which in *Dmel* responds to AITC (Figure 1C) and participates in mediating olfactory aversion to this odor compound (Figure Supplement 2G–H).

Given the need for finer olfactory resolution in mustard plant specialist like *Sfla*, we investigated whether the *Or*s in this lineage have undergone differentiation and specialization compared to those of more generalist microbe-feeding species like *Dmel* and the close relatives of the herbivorous *Scaptomyza*, which include the microbivores *Shsu* and *Spal*. We constructed functional maps of the palp olfactory sensilla across all five species and found that all *Scaptomyza* species, similarly to *Dmel* (Ray et al. 2008; Dweck et al. 2016), possess three distinct types of sensilla (Figure 2 and 3A, Figure Supplement 3). Of particular significance is the finding that while OSNs housed in pb1a sensilla from all species responded to volatile AITC, those housed in the pb1a-like sensilla of the mustard specialists *Sfla* and *Smon* additionally responded to other volatile ITCs (Figure 2). Furthermore, this sensilla type was expanded in these two species (Figure 3A and Figure Supplement 5).

Concomitantly with the functional and morphological changes described above, we found a duplication of the Or (*Or42a*) housed in these ITC-responding pb1a sensilla in the lineage leading to all known *Scaptomyza*, with the Brassicales-feeding *Sfla* exhibiting serial triplicates in one of the *Or42a* duplicates (*Or42a2*, *Or42a3*, and *Or42a4*; Figure 3B and Figure Supplement 7). Similarly, we previously reported that *Sfla* has a triplicated and positively selected Or (Or67b; Matsunaga et al. 2021) which is expressed in antennal OSNs. These Ors respond to aromatic and some aliphatic ITCs in a paralog-specific manner, while the *Dmel* and *Spal* Or67b single copies did not respond to any volatile ITCs (Matsunaga et al. 2021). However, all three *Sfla* Or67b paralogs showed poor responses to organosulfur ITCs, including AITC. In this study, on the other hand, we found that all three *Sfla* Or42a paralogs responded to AITC (Figure 4B). Thus, the gene duplications and amino acid substitutions of Or42a, along with those of Or67b, likely play an important role in enabling Brassicales-plant specialist *Scaptomyza* species to detect a wide range of ITCs, including specific blend ratios and concentrations, facilitating effective host plant location.

Although the pb1a sensilla of the two mustard specialist species have a broad ITC response range (Figure Supplement 4A and D), *Sfla* has triplicated *Or42a*s, whereas *S. montana* (*Smon*) has only one copy (Figure Supplement 7). This suggests that both the mustard plant specialization and the mutations underlying the expanded ITC sensitivity range of Or42a preceded the triplication of *Or42a*. What is then the adaptive value of the *Or42a* triplication? We observed that *Sfla* pb1a OSNs, which express at least one of the three triplicated Or42as (Figure 3B–C), exhibited reduced sensitivity to fruit-borne γ-hexalactone in comparison with *Smon* pb1a OSNs (Figure Supplement 4B). Only *Sfla* Or42a4 acquired the two key mutations that increased the sensitivity BITC to γ-hexalactone ratio (Figure 4E–F), as evidenced by the weaker γ-hexalactone response of the chimeric Or compared to *Sfla* Or42a3 (Figure 4B). In agreement with these observations, structural alignment of the 3D models predicted by AlphaFold2 revealed that *Smon* Or42a2 aligned well with *Sfla* Or42a3, but not with *Sfla* Or42a4 (Figure Supplement 10). Based on these findings, we hypothesized the following sequence in the evolution of Or42a (Figure Supplement 12): 1) mustard specialization and broadening of ITC sensitivity in the ancestral drosophilid Or42a that was already sensitive to some ITCs like AITC; 2) speciation and triplication of Or42a in *Sfla* but not in *Smon*; 3) relaxation of evolutionary constraints due to gene duplications, allowing *Sfla* Or42a4 to reduce its sensitivity to γ-hexalactone. Thus, although mustard specialization preceded the Or42a duplication, gene duplication appears to be important in driving adaptation to new ecological niches.

Finally, what is the functional relevance of ITC-sensitive Ors expressed in various olfactory organs? *Or67b* in *Sfla* is primarily expressed in the antennae (Matsunaga et al. 2021), whereas *Or42a* in *Sfla* and the other drosophilids is expressed in the maxillary palps (Figure 3C). In *Dmel*, maxillary palps OSNs have lower sensitivity thresholds to certain host-related compounds compared to antennal OSNs (Dweck et al. 2016). Indeed, we found that the *Sfla* Or42a paralogs are much more sensitive to AITC than the Or67b paralogs (Figure 4, Matsunaga et al. 2021). Odor response redundancy between antennal and maxillary palp Ors could have evolved to further underpin olfactory orientation over both long and short distances in drosophilids (Dweck et al. 2016). Given the proximity of Or42a OSNs to the mouthparts, their activation could potentially modulate feeding behaviors, as suggested in *Dmel* (Shiraiwa 2008). *Sfla* and *Smon* adult females feed on the juice that seeps into the leaf wounds they create in Brassicales plants before oviposition (Peláez et al. 2022). Given this stereotyped feeding behavior of adult females, while the activation of contact chemoreceptors by ITCs could help females assess the suitability of an oviposition site through taste, flies may also be aided by olfactory activation via the maxillary palps perhaps even before tasting the plants given their close proximity to the wounds before feeding.

### Differential expression of *Or*s in *Scaptomyza*

Our maxillary palp RNA-seq expression data (Figure 3C), combined with electrophysiological results showing similar patterns of odor responses in pb1-like and pb2-like sensilla OSNs (Figure 2), suggest that all *Scaptomyza* species express the following *Or*s in palp sensilla OSNs: *Or42a* in pb1a-like, *Or71a* in pb1b-like, *Or33c* and *Or85e* in pb2a-like, and *Or46a* in pb2b-like. This raises the question of which *Or*s are expressed in OSNs of pb3a-and pb3b-like sensilla in *Scaptomyza* spp. Based on our RNA-seq data, candidates included *Or67a, Or98b, Or59a,* and *OrN2a*, as they were robustly expressed in the maxillary palps (Figure 3C). Among these, only Or67a has been functionally characterized in *Dmel*, where it responds to 2-heptanone (Auer et al. 2020). Our SSR experiments showed that 2-heptanone also activated OSNs in pb3a-like (Figure 2), raising the possibility that *Scaptomyza* pb3a-like OSNs express Or67a. Alternatively, other yet uncharacterized Ors responsive to 2-heptanone might be expressed in these OSNs.

### Functional evolution of ITC-responding Ors coupled with increases in OSNs

Using HCR RNA FISH, we discovered an increase in the number of *Or42a*-positive-OSNs in *Sfla* compared to *S. pallida* (*Spal*) (Figure 3D–F). Similar increases in OSNs which detect odors that bear biological significance for insects have been reported in several *Drosophila* species. For instance, the noni fruit specialist *D. sechellia* and the seasonal specialist of screw pine fruits *D. erecta* both show an increase in the number of *Or22a^+^* OSNs (Dekker et al. 2006; Linz et al. 2013; Auer et al. 2020). In *D. sechellia*, these OSN increases have been shown to enhance odor tracking by reducing adaptation in second-order projection neurons (Takagi et al. 2024). We hypothesize that the increase in the number of *Or42a*-positive-OSNs in *Sfla* may similarly contribute to enhance odor sensitivity and tracking during host plant finding, although this remains to be investigated.

### Insight into the binding pocket of Or42a paralogs

Our results demonstrated that the Or42a paralogs from microbe-feeding species show strong responses to both AITC and γ-hexalactone (Figure 4), while the paralogs from the herbivorous *Sfla* show a notable shift in olfactory sensitivity. For example, *Sfla* Or42a3 showed moderate responses to γ-hexalactone (about an order of magnitude lower than those of the Or42a from the microbe-feeding *Spal*), and *Sfla* Or42a4 showed even less spiking activity (Figure 4D–E). Intriguingly, *Sfla* Or42a4 has a relatively higher sensitivity to BITC compared to Or42a3 (Figure 4B and D–E). These findings align with the fact that *Sfla* has undergone a full transition to herbivory. To explore the mechanisms underlying this shift from fruit-detector to ITC-detector, we employed computational and functional approaches to identify the amino acid substitutions responsible for the differences in odor sensitivities between *Sfla* Or42a3 and Or42a4.

The binding pocket of Ors is located within the transmembrane region, and amino acid substitutions in these regions can alter the ligand’s binding affinity [(Del Mármol et al. 2021; Zhao et al. 2024), although extracellular loops may also play an important role (Carraher et al. 2015)]. Our AlphaFold2-led screening for amino acid substitutions identified two key substitutions in the transmembrane portion: A181D and S307P (Figure 4C). We substituted these two amino acids and found that OSNs expressing this chimeric Or have an increased BITC to γ-hexalactone response ratio (Figure 4E–F). Proline is known to be a secondary structure breaker (Chou and Fasman 1978; Levitt 1978), and the substitution of serine by proline (S307P) likely influenced the conformation change of the binding pocket, altering the protein structure, polarity, and hydrophobicity. Similarly, the substitution of alanine by aspartic acid (A181D) can alter protein polarity and hydrophobicity. Thus, these two amino acid changes likely account for the notable change in ligand sensitivity. However, the increased BITC to γ-hexalactone spike ratio for was observed only in flies heterozygous for A181D and S307P (Or67d^Gal4^; UAS-A181D S307P/+), but not in the flies homozygous for these two amino acid substitutions (compare Figure 4E–F with Figure Supplement 11). Given that homozygous flies express A181D and S307P more strongly than the heterozygous flies, it is possible that the OSNs’ response to both BITC and γ-hexalactone reached saturation in homozygous flies, obscuring further differences in spike ratios. Alternatively, excessive expression of A181D and S307P may have altered the 3D structure of the protein, reverting it closer to the original *Sfla* Or42a3 conformation and restoring the original binding pocket. Nonetheless, the partial rescue we observed in heterologous flies indicates that A181D and S307P are critical for altering the binding pocket structure, enabling the binding pocket of Ors to better accommodate BITC instead of γ-hexalactone.

## CONCLUSIONS

Taken together, our findings reveal that generalist insects like *D. melanogaster* have evolved olfactory sensory mechanisms that allow them to detect and avoid plant-derived volatile electrophilic toxins, such as ITCs. In contrast, Brassicales plant specialists like *S. flava* not only have physiological adaptations to detoxify these toxic compounds but have undergone significant evolutionary sensory adaptations for host recognition, including specialized ITC olfactory receptors to aid host plant location. These adaptations involve the triplication of homologous Ors in the antennae (Matsunaga et al. 2021), as well as in the maxillary palps (this study), which collectively expanded the sensitivity and detection range for these and other signature hostplant odorants. Furthermore, our use of AlphaFold2, followed by site-directed mutagenesis and electrophysiology, identified critical amino acid changes for the evolution of specialized odorant receptors that we confirmed experimentally. This result also demonstrates the utility of machine learning algorithms as tools for evolutionary biology research, particularly in screening and identifying functionally relevant alleles for follow-up functional studies.

## Supplementary Material

Supplement 1 Supplementary file 1: Chemical list

Supplement 2 Supplementary file 2: Control-subtracted net number of spikes/sec and EC_50_ obtained from SSRs

Supplement 3 Supplementary file 3: Immobility assay data

Supplement 4 Supplementary file 4: Consumption assay data

Supplement 5 Supplementary file 5: Olfactory positional assay data and trap assay

Supplement 6 Supplementary file 6: Expression intensity from maxillary palp RNA-seq

Supplement 7 Supplementary file 7: OSNs counts from RNA FISH

Supplement 8 Supplementary file 8: Primer list

Supplement 9 Supplementary file 9: AlphaFold2 (Colabfold) prediction in pdb format

Supplement 10 Supplementary file 10: Pb sensilla counts from SSR experiments

Supplement 11 Supplementary file 11: Measurements (area) of maxillary palp and body

Supplement 12 Supplementary file 12: CDS of *Or42a* homologs in fasta format

Supplement 13 Supplementary file 13: Alignment 1130 of *Or42a* homologs in fasta format

14

## Data Availability

The data presented in this study are available on request from the corresponding author.
