# Supplementary material for: Odorant receptors mediating avoidance of toxic mustard oils in Drosophila melanogaster are expanded in herbivorous relatives
Source: bioRxiv. 2025 May 29:2024.10.08.617316. Originally published 2024 Oct 11. Preprint. [Version 3] doi: 10.1101/2024.10.08.617316 (PMC11482750; doi:10.1101/2024.10.08.617316)

bioRxiv preprint doi: https://doi.org/10.1101/2024.10.08.617316; this version posted May 29, 2025. The copyright holder for this preprint (which was not certified by peer review) is the author/funder, who has granted bioRxiv a license to display the preprint in perpetuity. It is made available under aCC-BY 4.0 International license.

FIGURE SUPPLEMENT CAPTIONS:

Figure Supplement 1: Schematic representations of the behavioral assays. (A) Mobility assay used as a proxy for intoxication. Mated female flies (n=10) were placed in the upper chamber (lateral view) and monitored every 10 minutes in presence of volatile chemicals (4 drops of a 5 µl solution in the lower chamber, top view). The top and the bottom chambers were separated by a fabric mesh, preventing direct contact between the flies and the chemical solutions. **(B)** Positional olfactory assay. Groups of 10-12 non-starved cold-anesthetized mated females were placed in a small piece of clear Tygon tube, which was then used to connect the narrow ends of two cut glass Pasteur pipettes (non-choice/release zone). The distal end of each glass pipette was connected to a glass vial containing 10 µL of the odor solution or the solvent control; a piece of fabric mesh prevented insects from entering the vials containing the odor or control solutions. The number of flies in each of the two tubes, as well as in the middle release section, were counted every five minutes until minute 35, and in tests with allyl ITC, again at 65 minutes. The % of insects that choose one or the other tube over the total number of insects released, and the % of insects in the tube closest to the odor source over the total number of insects that choose one or the other tube, was then calculated for each time point. **(C)** Feeding assay in presence of odors or the solvent control. In each test a group of 24 hours wet-starved mated female flies (n=11-15) was placed in a vial containing a piece of filter paper impregnated with 160 µl of 50 mM D-glucose dyed

bioRxiv preprint doi: https://doi.org/10.1101/2024.10.08.617316; this version posted May 29, 2025. The copyright holder for this preprint (which was not certified by peer review) is the author/funder, who has granted bioRxiv a license to display the preprint in perpetuity. It is made available under aCC-BY 4.0 International license.

blue. A small container with a mesh bottom was affixed to the inner side of the vial cap contained a piece of filter paper loaded with 10 µl of an odorant solution or the solvent control, such as that flies could smell but not contact the odor source. After 15 minutes, vials were frozen for at least 60 minutes, the amount of blue dye in the abdomen of each fly in each vial was quantified blind to treatment, and a feeding score was calculated for each vial as explained in materials and methods.

Figure Supplement 2: Behavioral data related to Figure 4. Positional olfactory assays. **(A-B)** Tests with allyl isothiocyanate (AITC) 1:500 vol/vol vs mineral oil (as in Figure 4A) using Canton-S flies (n=19); flies preferred the odorless tube from minute 25 onwards (one sample signed rank tests against median=50%, *p<0.05 in all cases; **A**). Total choice of Canton-S flies calculated as: [(# of flies in the odorless tube + # of flies in the odorous tube) / (# of flies released) *100)]; **B**. **(C)** Total choice of genetic background control (line # 68384) and *Or42a-/-* mutants (n=15 for each genotype). Similar percentages of control flies and *Or42a-/-* mutants choose one or the other tube at all time points, indicating that all fly genotypes are similarly active in presence of AITC volatiles (Mann-Whitney U tests, p>0.05 for all time points). **(D-E)** Tests using γ-hexalactone 1:10 vol/vol vs the solvent control using genetic background control flies and *Or42a-/-* mutants. Control flies choose the odorous tube at 20 and 30 minutes (n=19, *p<0.05, one-sample signed rank tests), while mutants distributed randomly at all time points (n=19, p>0.05 in all cases); **D**. Additionally, the total choice of control flies was larger than that of mutant flies at all time points, suggesting that the presence of the odor increases exploration activity in control flies (Mann-Whitney U tests, *p<0.05, ***p<0.005); **E**. Canton-S flies also showed attraction to this odor (at 5, 10, and 15 minutes, n=12; one-sample signed rank tests, p<0.05 at all those three times, data shown in Supplementary File 4). **(F-G)** Tests with *Or7a-/-* (n=15) and genetic background control flies (n=18) using AITC 1:500 vol/vol vs mineral oil. Control flies avoided the AITC tube at all time points, while mutants only avoided at 30 minutes and onwards (one-sample signed rank tests, 1-tail, *p<0.05, ****p<0.001); **F**. The total choice of flies was not different between genotypes (p>0.05 at all time points); **G**. **(H-I)** Tests with *Or35a-/-* (n=29) and genetic control flies (n=27) using γ-hexalactone 1:10 vol/vol vs the solvent control. Control flies choose the odorous tube at various time points (*p<0.05, ***p<0.001, one sample signed rank tests, 1 tail), while mutants always distributed at random (p>0.05 in all cases); **H**. **I:** Total choice of control and Or35a-/- mutants; differences between genotypes were not statistically different (Mann-Whitney U tests, p>0.05 at all time

bioRxiv preprint doi: https://doi.org/10.1101/2024.10.08.617316; this version posted May 29, 2025. The copyright holder for this preprint (which was not certified by peer review) is the author/funder, who has granted bioRxiv a license to display the preprint in perpetuity. It is made available under aCC-BY 4.0 International license.

points). In all cases (panels **C-I,** Figure 4A) mutants of the various genotypes used were assayed in parallel with the genetic background control line and therefore, control data was obtained from different subsets of flies. **(J)** Feeding assay, as in Figure 1E, but using γ-hexalactone (1:50 vol/vol). The dotted line at 50% indicates neither feeding enhancement or repellence. Canton-S flies consumed more in presence than in absence of γ-hexalactone (one-sample signed rank test on normalized data against expected median=1, $p<0.001$, n=25), and *Or42a-/-* mutants instead consumed less in presence of the odor; $p<0.001$, n=24). **(K)** Mobility assay, as in Figure 2, but using γ-hexalactone at various concentrations from 1:500 to 1:2.5 vol/vol. n=10 for each condition (solvent and concentration). Flies remained active in the presence of this volatile. **(L)** Single sensillum recordings from all *D. melanogaster* antennae (ab) and palp basiconic (pb) sensilla upon stimulation with γ-hexalactone 1:100 vol/vol. Only pb1a was activated by γ-hexalactone (n= 3-6 OSNs). **(M)** Activation of Or42a, in combination with distinct olfactory channels, is involved in mediating both olfactory repellence to AITC (light blue arrow) and attraction to γ-hexalactone (orange arrow). Activation of Or42a in combination with Or7a or O35a likely mediates repellence and attraction, respectively.

Figure supplement 3: Functional characterization of *D. melanogaster* and *Scaptomyza* species maxillary palp OSNs housed in the three palp basiconic sensilla types.

Single sensillum recordings from maxillary palp OSNs (pb2, pb2-like, pb3, and pb3-like) of *D. melanogaster, S. hsui, S. pallida, S. flava,* and *S. montana*. Stimuli (1:100 vol/vol) included diagnostic chemicals used to identify Ors in *D. melanogaster* (see Methods), fruit volatiles, green leaf volatiles, and Brassicales plant-derived isothiocyanates (ITCs) (n=6-9 from 3-4 animals/species). Pb and pb-like sensilla housed two OSNs, labeled "a" (darker color) and "b" (lighter color). Their response profiles and stereotyped locations within the palp support the classification of *Scaptomyza* sensilla into three types, pb1-like, pb2-like, and pb3-like (see also Figure 5). Methyl salicylate (a *S. flava* volatile attractant in natural settings, Orre et al. 2010) activated all *Scaptomyza* pb2b-like sensilla but not *D. melanogaster* pb2b (first row). The response profiles of *Scaptomyza* pb3-like OSNs were different from those of *D. melanogaster* pb3, likely because the homologs of *Or59c* and *Or85d*, which are respectively expressed in *D. melanogaster* pb3a and p3b, are unidentified in the genomes of *Scaptomyza* (Goldman-Huertas et al. 2015). The odorant response profiles of OSNs in pb3-like sensilla were more similar between the more distantly related species *S. hsui* and *S. pallida,* than between the more closely related *S. pallida* and

bioRxiv preprint doi: https://doi.org/10.1101/2024.10.08.617316; this version posted May 29, 2025. The copyright holder for this preprint (which was not certified by peer review) is the author/funder, who has granted bioRxiv a license to display the preprint in perpetuity. It is made available under aCC-BY 4.0 International license.

S. flava, or *S. pallida* and *S. montana.* For example, *trans*-2-hexenal activated these OSNs in *S. hsui* and *S. pallida* (but not in *S. flava* or *S. montana*), while phenethyl acetate activated *S. flava* and *S. montana* OSNs (but not *S. hsui* or *S. pallida*). AITC: allyl ITC, IBITC: isobutyl ITC, BITC: butyl ITC, SBITC: *sec*-butyl ITC, PITC: phenethyl ITC.

Figure Supplement 4: Hierarchical cluster analysis of maxillary palp sensilla based on odorant response profiles across species. (A) Hierarchical clusters were constructed using odorant response data from single sensillum recordings of basiconic palp sensilla from *D. melanogaster, S. hsui, S. pallida, S. flava,* and *S. montana* using R studio v1.4.1717. A terminal node corresponds to a single recording from the individual sensilla; n=6-8 sensilla from 3-4 animals. **(B)** Hierarchical clusters were created for each sensilla type, including pb1-like, pb2-like, and pb3-like, using the odorant response (control-subtracted net number of spikes) average from each species as inputs. **(C)** The species phylogeny (left: Kim et al., 2021) and palp functional dendrogram (right) highlight the relevance of niche differences in shaping odorant responses. The palp functional dendrogram was generated using odorant response averages from each species.

Figure Supplement 5. Responses of OSNs from *D. melanogaster* pb1a and *Scaptomyza* pb1a-like sensilla to stimulation with various odorant concentrations. (A) Responses of pb1a OSNs across species upon stimulation with various concentrations (ranging from 1:10^{-2} to 10^{-5} vol/vol) of ethyl butyrate, γ-hexalactone, *trans*-2-hexenal, allyl isothiocyanate (AITC), isobutyl isothiocyanate (IBITC), butyl isothiocyanate (BITC) and *sec*-butyl isothiocyanate (SBITC; n=6-8 sensilla from 3-4 animals/species). The chemical structures of each compound are shown on the left. IBITC, BITC, and SBITC were only tested in *S. flava* and *S. montana* pb1a, as BITC at 10^{-2} vol/vol failed to activate OSNs in pb1a from the two microbe-feeding species (Figure 5). **(B-C)** Responses of *S. flava* and *S. montana* pb1a OSNs upon stimulation with γ-hexalactone 10^{-4} vol/vol **(B)** and the green leaf volatile *trans*-2-hexenal 10^{-4} vol/vol **(C)**; n=6-7 sensilla from 3 animals/species. Mann-Whitney U tests: *$p<0.05$, **$p<0.01$. **(D)** Half maximal effective concentrations (EC_{50}) for each fly species and odorant (see Methods for calculations of EC_{50}). Kruskal-Wallis tests followed by Dunn's multiple comparisons. Different letters indicate significant differences between species (p-values for ethyl butyrate: *S. pallida* vs *S. flava* $p<0.05$, *S. pallida* vs *S. montana* $p<0.01$; for γ-hexalactone: $p<0.001$; for *trans*-2-hexenal: *D.*

bioRxiv preprint doi: https://doi.org/10.1101/2024.10.08.617316; this version posted May 29, 2025. The copyright holder for this preprint (which was not certified by peer review) is the author/funder, who has granted bioRxiv a license to display the preprint in perpetuity. It is made available under aCC-BY 4.0 International license.

melanogaster vs *S. montana* $p<0.05$, *S. hsui* vs *S. flava* $p<0.01$, *D. melanogaster* vs *S. flava* $p<0.0001$; for AITC: $p<0.05$). The EC_{50s} of *S. flava* and *S. montana* to BITC, IBITC and SBITC were not statistically different (Mann-Whitney U tests, $p>0.05$). Overall, pb1a OSNs of microbe-feeding species exhibited greater sensitivity to γ-hexalactone, whereas Brassicales specialists showed higher sensitivity to *trans*-2-hexenal and AITC. *S. flava* and *S. montana* showed similar sensitivities to all four ITCs tested.

Figure Supplement 6: Or42a gene tree constructed by RAxML, and syntenic maps and dot plots visualized by DiGAlign. (A) Maximum-likelihood gene tree with 1000 bootstrap cycles using *S. hsui Or42a1*, *S. pallida Or42a1*, *S. graminum Or42a1*, *S. montana Or42a1*, *S. flava Or42a1*, *S. hsui Or42a2*, *S. pallida Or42a2*, *S. graminum Or42a2*, *S. flava Or42a2*, *S. montana Or42a*, *S. flava Or42a2*, *S. flava Or42a3*, *S. flava Or42a4*, and *D. melanogaster Or42a* as an outgroup, generated by RAxML (v8.2.19) (Stamatakis 2014) after alignment by MAFFT v7 (Katoh and Standley 2013). Bootstrap values are shown on the branches when the values were lower than 80. The tree was displayed by FigTree v1.4.3 (Rambaut 2009). **(B)** Dot plots (left) and synteny maps (right) were generated for the *Or42a2-Or42a4* genomic region across four species pairs: *S. flava*-*S. montana*, *S. montana*-*S. graminum*, *S. graminum*-*S. pallida*, and *S. pallida*-*S. hsui*. A 60,199-nt region was analyzed in *S. flava*, and 30,000-nt regions were analyzed in the other four species. Genomes of *S. montana, S. graminum,* and *S. pallida* were reversed-aligned for visualization. Each genomic region is color-coded according to BLASTn percent identity (top left). Cyan rectangles mark the span from the start to the end of *Or42a* homologs. *S. flava* has three copies of *Or42a2* species-specific, separated by intronic intervals that are mostly not conserved in the other species. In contrast, the remaining *Scaptomyza* species showed conserved synteny at the *Or42a2* locus.

Figure Supplement 7: Maxillary palp RNA seq of *S. pallida* and *S. flava Or*s. The heatmap displays Log2[RPM+1] values of *Or*s expression in the maxillary palps of female (F) and male (M) *S. flava* and *S. pallida* (n=3/sex). Each column represents a replicate (e.g. *S. flava* F1: replicate #1 of female *S. flava*, *S. flava* M1: replicate #1 of male *S. flava*). Gray boxes indicate that the corresponding genes were unidentified in the genomes of the species. Only one copy of Or42a (Or42a2) is expressed in *S. pallida*, while *S. flava* expresses three copies. We confirmed the expression of homologs *Or71a* (expressed in OSNs of *D. melanogaster* pb1b), and of *Or33c/Or85e* and *Or46a* (expressed in OSNs of *D.*

bioRxiv preprint doi: https://doi.org/10.1101/2024.10.08.617316; this version posted May 29, 2025. The copyright holder for this preprint (which was not certified by peer review) is the author/funder, who has granted bioRxiv a license to display the preprint in perpetuity. It is made available under aCC-BY 4.0 International license.

melanogaster pb2a and pb2b) in both *S. pallida* and *S. flava* maxillary palps. *Or85d* and *Or59c* were respectively expressed in *D. melanogaster* pb3a and pb3b OSNs but the homologs were unidentified in the genomes of *Scaptomyza* (Goldman-Huertas et al., 2015). Instead, *Or59a1, Or67a1, Or67a2,* and *OrN2a* were strongly expressed in OSNs of both *S. pallida* and *S. flava,* suggesting that these homologs may be expressed in pb3a or pb3b.

Figure Supplement 8: Over-representation of pb1-like sensilla in mustard plant specialists. Picture of single sensillum recording from *S. flava* maxillary palp sensilla (top left) and a representative anatomical mapping of *S. flava* palp sensilla (bottom left) obtained using diagnostic chemicals. Green: pb1-like, red: pb2-like, and magenta: pb3-like. Boxplots in the graph (right) represent the number of each sensilla type in *D. melanogaster* and the four *Scaptomyza* species (n=3 animals/species; see Figure Supplement 9 for individual maps). Maxillary palp pb1 sensilla are over-represented in *S. flava* and *S. montana*, the two mustard specialists.

Figure Supplement 9: Location and percentages of the three different maxillary palp basiconic (pb) sensilla across species. Classification of maxillary palp pb sensilla using single sensillum recordings and stimulation with diagnostic odorants. Data represents the proportion of pb1 and pb1-like (green), pb2 and pb2-like (red), and pb3 and pb3-like (magenta) in *D. melanogaster, S. hsui, S. pallida, S. flava,* and *S. montana*. The schematic positions of pb sensilla in each of the three replicates (R1, R2, and R3) from each species are shown on the right. The total number of sensilla identified in each animal is indicated between parentheses.

Figure Supplement 10: Alignment of 1D and 3D structures of amino acids of Or42a. (A) The 1D alignment of amino acids of *S. pallida* Or42a2, *S. flava* Or42a3, *S. flava* Or42a4, and *S. montana* Or42a2. Red arrowheads on top denote the sites where site-directed-mutagenesis was performed to generate the A181D S301P chimera. **(B)** The 3D structures of *S. flava* Or42a3, *S. flava* Or42a4, A181D S301P, and *S. montana* Or42a2 were predicted using AlphaFold2. The colors of the amino acids indicate the predicted local distance difference test (pLDDT) scores: blue (>90), cyan (>70), yellow (> 50), and orange (<50). We confirmed that the scores for these non-overlapping sites were above 70, indicating that the 3D predictions of these sites are reliable.

bioRxiv preprint doi: https://doi.org/10.1101/2024.10.08.617316; this version posted May 29, 2025. The copyright holder for this preprint (which was not certified by peer review) is the author/funder, who has granted bioRxiv a license to display the preprint in perpetuity. It is made available under aCC-BY 4.0 International license.

Figure Supplement 11: Screening of candidate amino acids by AlphaFold2 3D prediction. (A) Lateral view of the 3D structure of A181D S301P predicted by AlphaFold2 (left). *S. flava* Or42a3-derived amino acids are indicated in magenta, and the substitutions with *S. flava* Or42a4-derived amino acids are represented in green. The middle and right panels respectively display side and top views of alignment of the predicted structures of *S. flava* Or42a3, *S. flava* Or42a4, and A181D S301P. The extracellular region at S5 and S6 (red arrows) highlights the local structural differences between *S. flava* Or42a3 and Or42a4 where the A181D, S301P overlaps with the local structure of *S. flava* Or42a4. **(B)** The 3D predictions of *S. flava* Or42a3 (magenta), *S. flava* Or42a4 (green), and A181D S301P (cyan) were aligned using PyMol 2.5.3. Red rectangles highlight the sites of local structural variance between *S. flava* Or42a3 and Or42a4. The A181D S301P shows a closer alignment with Or42a4. S5' and S6' are enlarged views of S5 and S6, respectively. **(C)** The enlarged views of S5 and S6 in the alignment of 3D predictions of *S. flava* Or42a3 (magenta), *S. flava* Or42a4 (green), and *S. montana* Or42a2 (cyan). **(D)** The 3D alignment of *S. flava* Or42a3 and *S. montana* Or42a2 predicted by AlphaFold2. The root mean square deviation (RMSD) is visualized with a color gradient.

Figure Supplement 12: Responses of OSNs expressing homozygous Or42a to stimulation with butyl isothiocyanate and γ-hexalactone. Left: responses of OSNs expressing homozygous *S. flava* Or42a3, *S. flava* Or42a4, or A181D S307P (*UAS-Or42a; Or67d* ^{Gal4}) to stimulation with butyl isothiocyanate (BITC, white) and γ-hexalactone (grey) (n=16-30 from 5-10 animals). Right: ratio between responses to BITC and the sum of the responses evoked by stimulation with BITC and γ-hexalactone. Kruskal-Wallis followed by Dunn's multiple comparisons, *p<0.05.

Figure Supplement 13: A model for the evolution of Or42a in *S. flava, S. montana* and *S. pallida*. The evolution of Or42a begins with a shift in the ligand specificity of an ancestral Or42a (a), which was tuned to fruit-borne odors such as γ-hexalactone and limited ITCs (i.e. AITC), and later it broadened to detect a wider range of ITCs (b). Subsequent gene triplication of *S. flava Or42a* resulted in three paralogous genes (c: *S. flava Or42a2, S. flava Or42a3,* and *S. flava Or42a4*). The lineage leading to *S. flava* Or42a2 and *S. flava* Or42a4 experienced a reduction in sensitivity to γ-hexalactone (arrow pointing

bioRxiv preprint doi: https://doi.org/10.1101/2024.10.08.617316; this version posted May 29, 2025. The copyright holder for this preprint (which was not certified by peer review) is the author/funder, who has granted bioRxiv a license to display the preprint in perpetuity. It is made available under aCC-BY 4.0 International license.

to the right in the bottom rightmost panel), while both *S. flava* Or42a3 and *S. montana* Or42a2 retained sensitivity to γ-hexalactone.

SUPPLEMENTARY MATERIAL AND METHODS:

Dilution series

Trans-2-hexenal, AITC, butyl ITC, isobutyl ITC, *sec*-butyl ITC, and ethyl butyrate were diluted in mineral oil, and γ-hexalactone was diluted in DMSO to concentrations ranging from 10^{-5} to 10^{-2} vol/vol. Half maximal effective concentration (EC50) was generated by GraphPad Prism v10.2.1.

Construction of the Or42a gene tree

The amino acid sequences of *D. melanogaster Or42a,* and *S. flava Or42a1, S. flava Or42a2, S. flava Or42a3,* and *S. flava Or42a4* were obtained from Flybase (2024_01), and Goldman-Huertas et al. (2015). Blast searches were conducted to annotate *S. hsui Or42a1, S. pallida Or42a1, S. graminum Or42a1, S. graminum Or42a2, S. montana Or42a1, S. montana Or42a2, S. flava Or42a1, S. hsui Or42a2,* and *S. pallida Or42a2,* using *S. flava Or42a1* and *S. flava Or42a2* as query against the genomes of *S. hsui* (ASM1815282v1), *S. pallida* (ASM1815296v1), *S. graminum* (ASM1890183v1), and *S. montana* (ASM1890430v1). Amino acid sequences of *S. hsui Or42a1, S. pallida Or42a1, S. gramina Or42a1, S. graminum Or42a2, S. montana Or42a1, S. montana Or42a, S. flava Or42a1, S. flava Or42a2, S. flava Or42a3, S. flava Or42a4, S. hsui Or42a2, S. pallida Or42a2* (Katoh and Standley 2013)*,* and *D. melanogaster Or42a* were aligned by MAFFT v7.511 with JTT as scoring matrix for amino acid sequences with L-INS-i strategy. Maximum-likelihood gene tree with 1000 bootstrap cycles using the above sequences were constructed with RAxML (v8.2.19) (Stamatakis 2014). GAMMA model of rate heterogeneity was applied. CDS and alignment of *Or42a* are included in Supplementary files 12 and 13, respectively.

Alignment of *S. flava Or42a3* and *Or42a4,* and *S. montana Or42a2* using AlphaFold2 3D structural prediction

CDSs of *S. flava Or42a3*, *S. flava Or42a4,* and *S. montana Or42a2* were used as inputs into ColabFold (Jumper et al. 2021; Mirdita et al. 2022). The output models ranked first (rank1) were selected, visualized, and 3D-aligned by using PyMol2.5.3.

bioRxiv preprint doi: https://doi.org/10.1101/2024.10.08.617316; this version posted May 29, 2025. The copyright holder for this preprint (which was not certified by peer review) is the author/funder, who has granted bioRxiv a license to display the preprint in perpetuity. It is made available under aCC-BY 4.0 International license.

Syntenic analysis using DiGAlign

To analyze synteny around the *Or42a2-Or42a4* loci, we extracted genomic regions spanning a total of 30kb (15kb upstream and 15kb downstream of the *Or42a2*s start site) from the genomes of *S. montana* (ASM1890430v1), *S. pallida* (ASM1815296v1), and *S. hsui* (ASM1815282v1). For *S. flava* (sfla_v2), we extracted a 60,199 bp region, encompassing 15kb upstream of the *Or42a2* start site through 15kb downstream of the *Or42a4* start site (Kim et al. 2021; Peláez et al. 2023). For visualization purposes, the genomes of *S. montana, S. graminum,* and *S. pallida* were reverse-aligned. Synteny maps and dot plots were generated and visualized using DiGAlign (Nishimura et al. 2024).

bioRxiv preprint doi: https://doi.org/10.1101/2024.10.08.617316; this version posted May 29, 2025. The copyright holder for this preprint (which was not certified by peer review) is the author/funder, who has granted bioRxiv a license to display the preprint in perpetuity. It is made available under aCC-BY 4.0 International license.

Supp. Figure 1

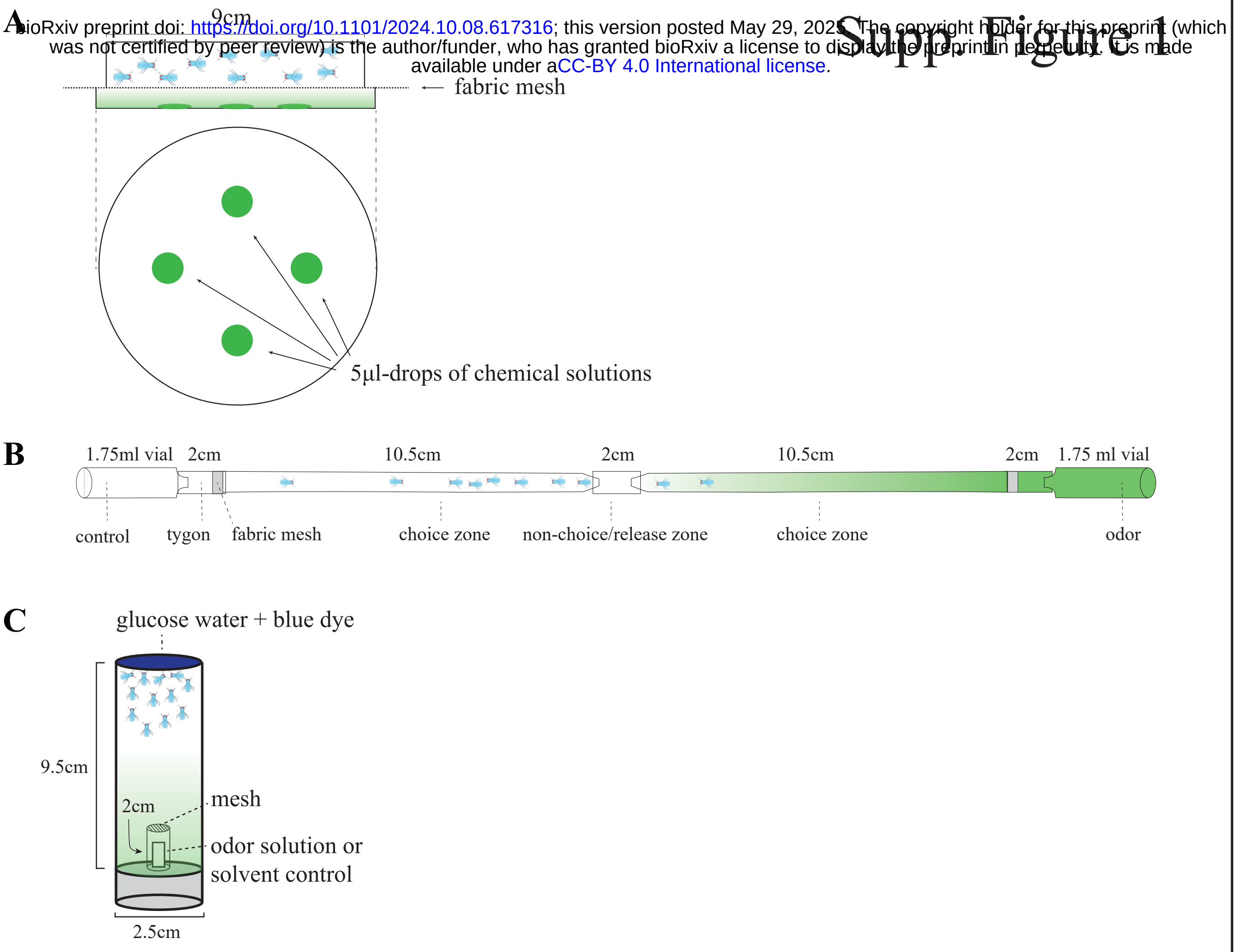

bioRxiv preprint doi: https://doi.org/10.1101/2024.10.08.617316; this version posted May 29, 2025. The copyright holder for this preprint (which was not certified by peer review) is the author/funder, who has granted bioRxiv a license to display the preprint in perpetuity. It is made available under aCC-BY 4.0 International license.

Supp. Figure 2

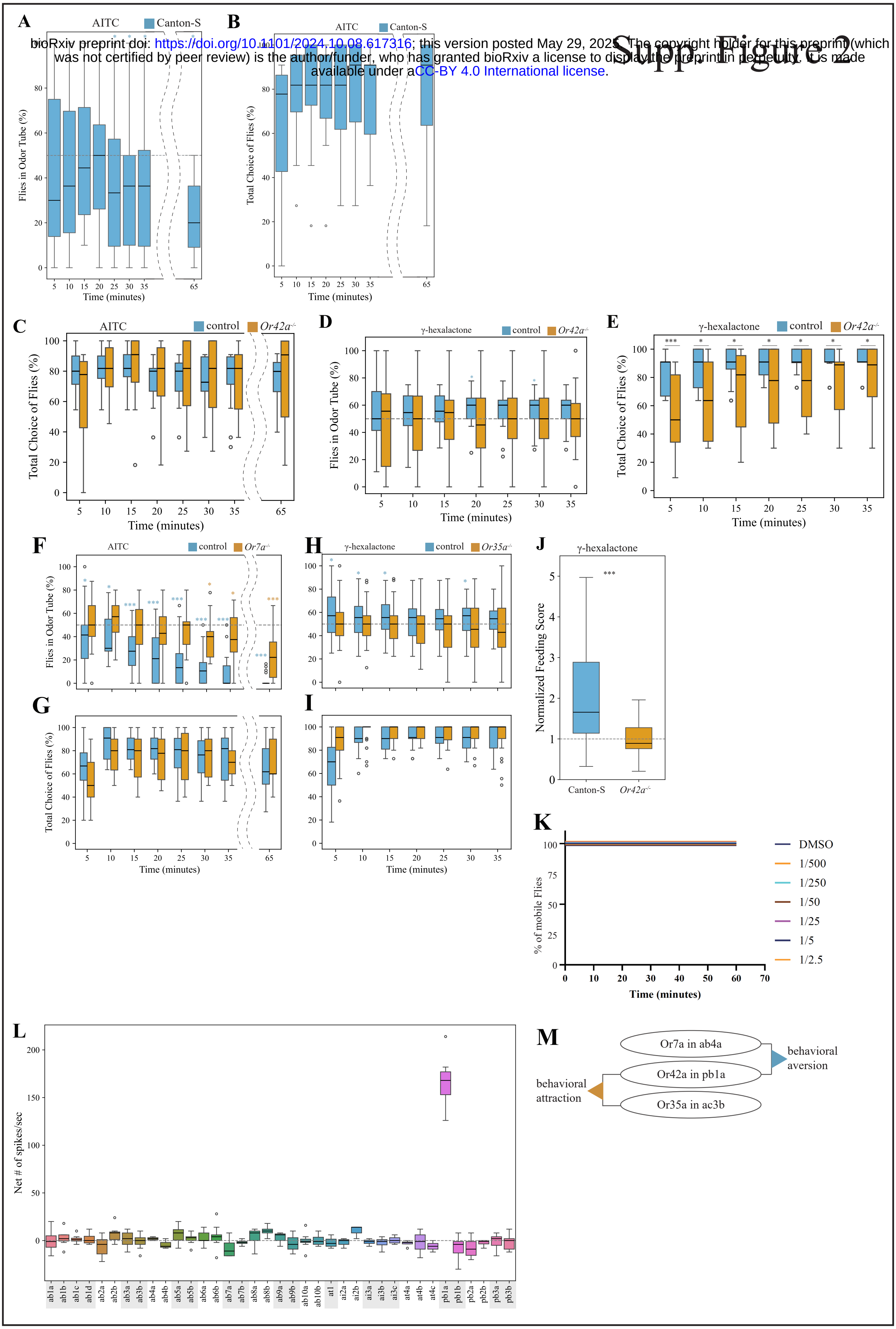

bioRxiv preprint doi: https://doi.org/10.1101/2024.10.08.617316; this version posted May 29, 2025. The copyright holder for this preprint (which was not certified by peer review) is the author/funder, who has granted bioRxiv a license to display the preprint in perpetuity. It is made available under aCC-BY 4.0 International license.

Supp. Figure 3

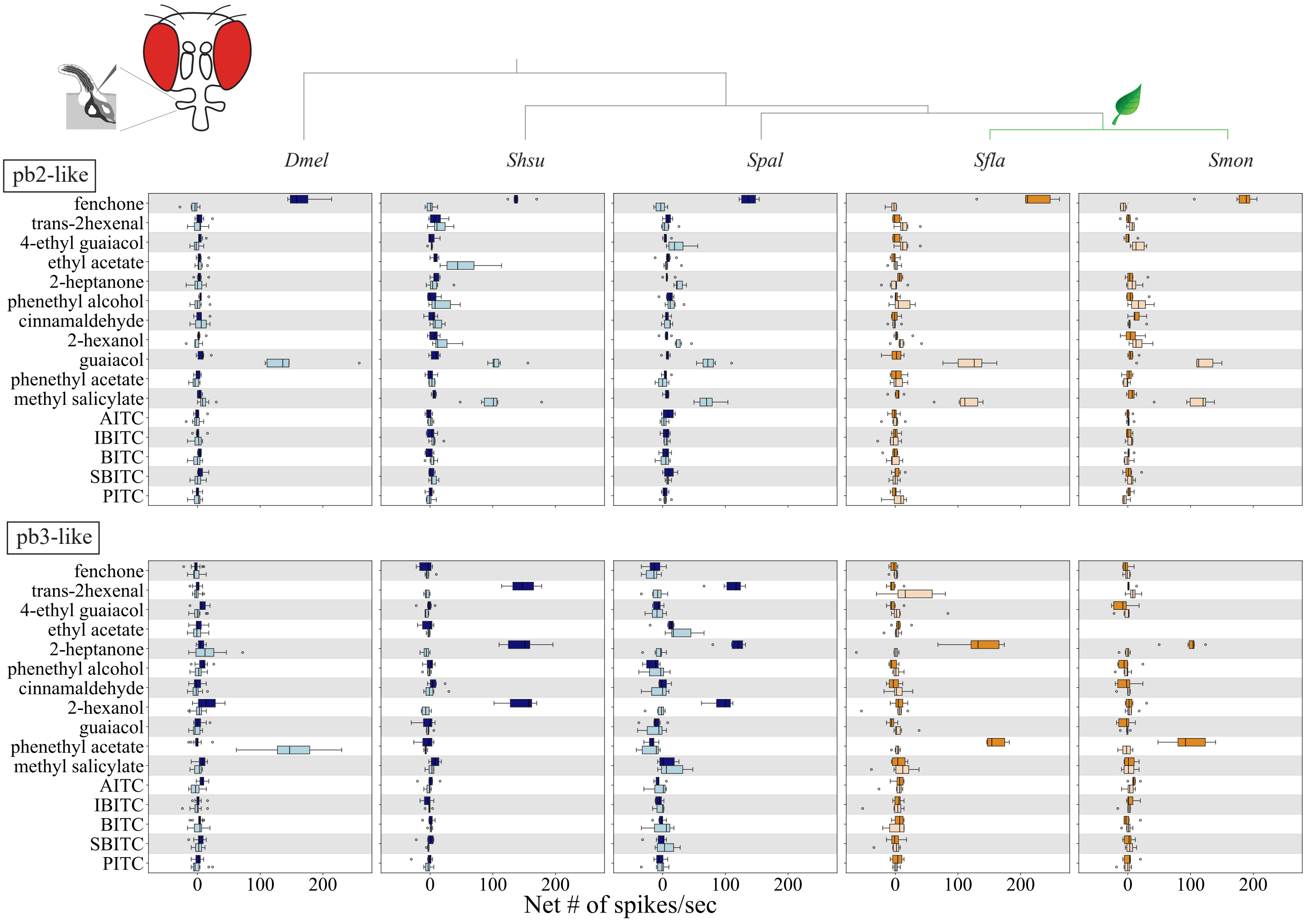

bioRxiv preprint doi: https://doi.org/10.1101/2024.10.08.617316; this version posted May 29, 2025. The copyright holder for this preprint (which was not certified by peer review) is the author/funder, who has granted bioRxiv a license to display the preprint in perpetuity. It is made available under aCC-BY 4.0 International license.

Supp.Figure 4

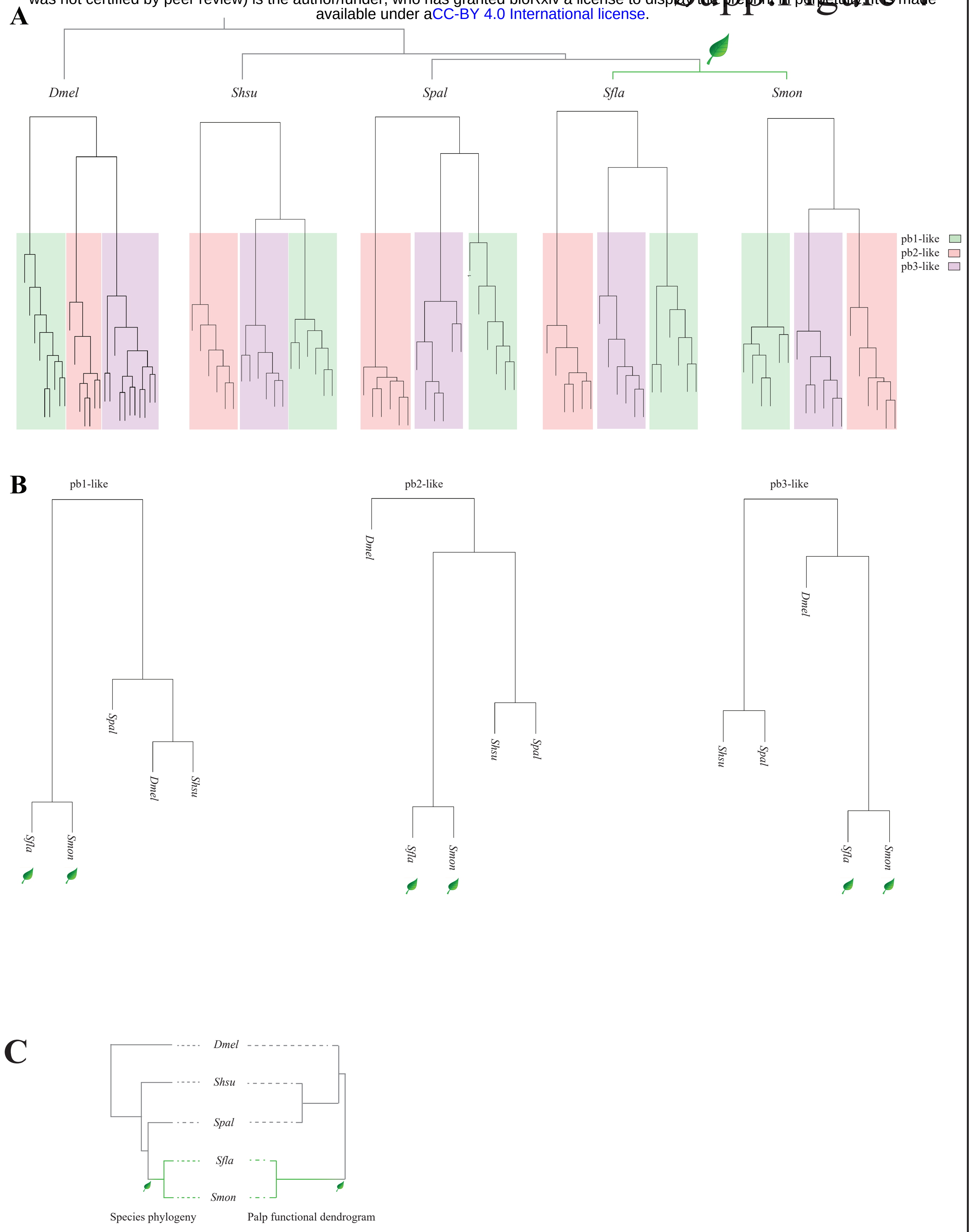

bioRxiv preprint doi: https://doi.org/10.1101/2024.10.08.617316; this version posted May 29, 2025. The copyright holder for this preprint (which was not certified by peer review) is the author/funder, who has granted bioRxiv a license to display the preprint in perpetuity. It is made available under aCC-BY 4.0 International license.

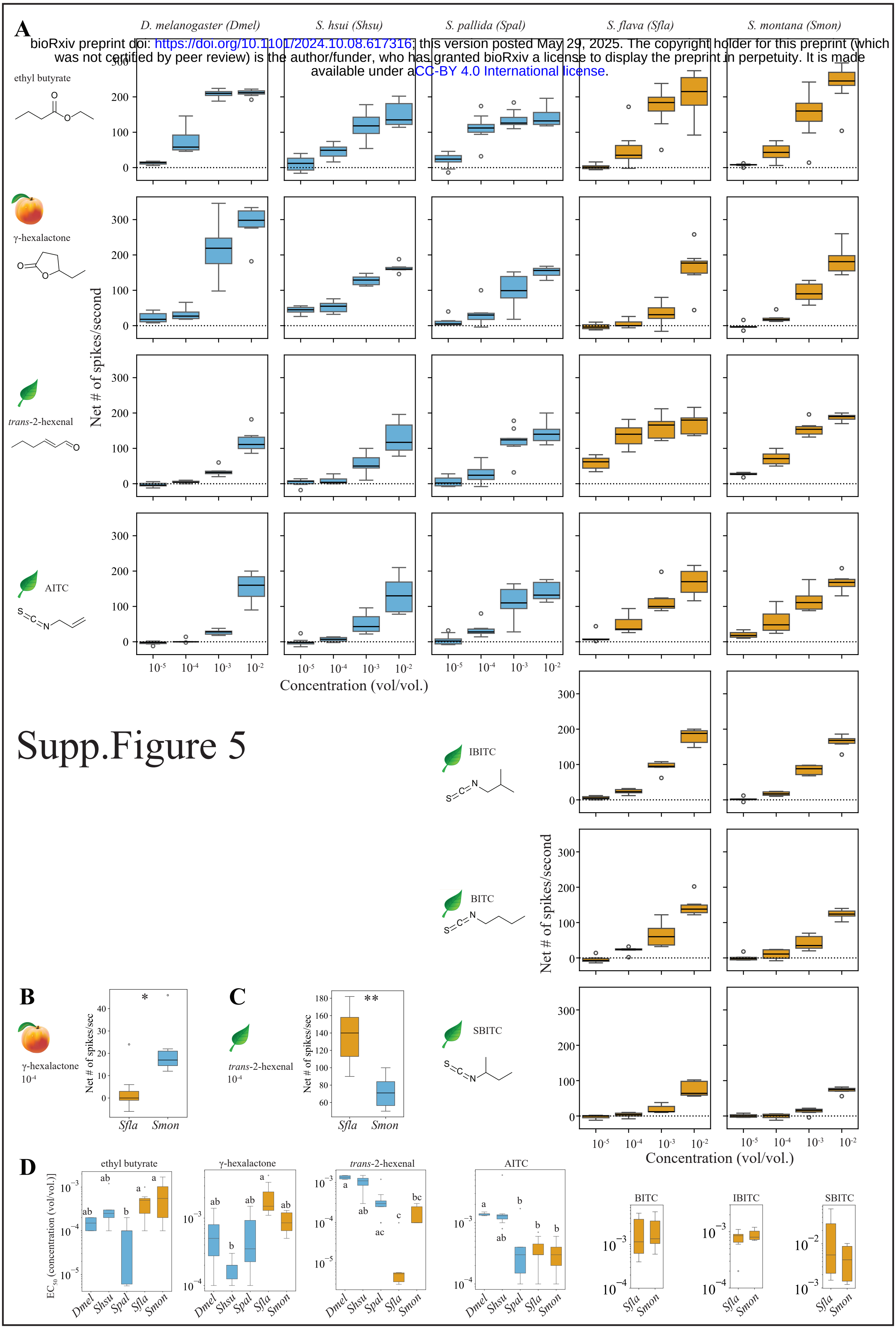

bioRxiv preprint doi: https://doi.org/10.1101/2024.10.08.617316; this version posted May 29, 2025. The copyright holder for this preprint (which was not certified by peer review) is the author/funder, who has granted bioRxiv a license to display the preprint in perpetuity. It is made available under aCC-BY 4.0 International license.

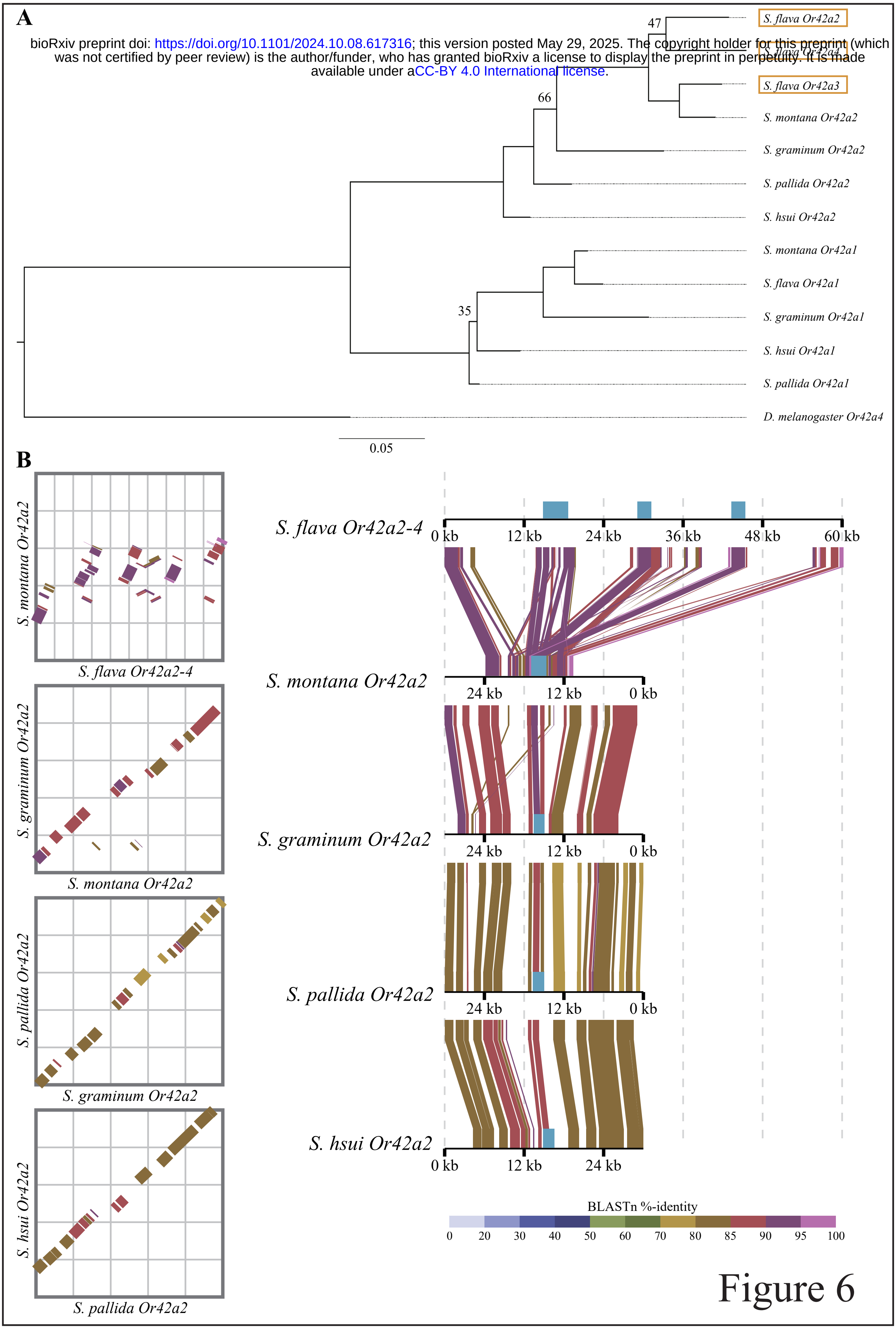

Figure 6

bioRxiv preprint doi: https://doi.org/10.1101/2024.10.08.617316; this version posted May 29, 2025. The copyright holder for this preprint (which was not certified by peer review) is the author/funder, who has granted bioRxiv a license to display the preprint in perpetuity. It is made available under aCC-BY 4.0 International license.

Supp. Figure 7

Or gene family expression (Log2[RPM+1])

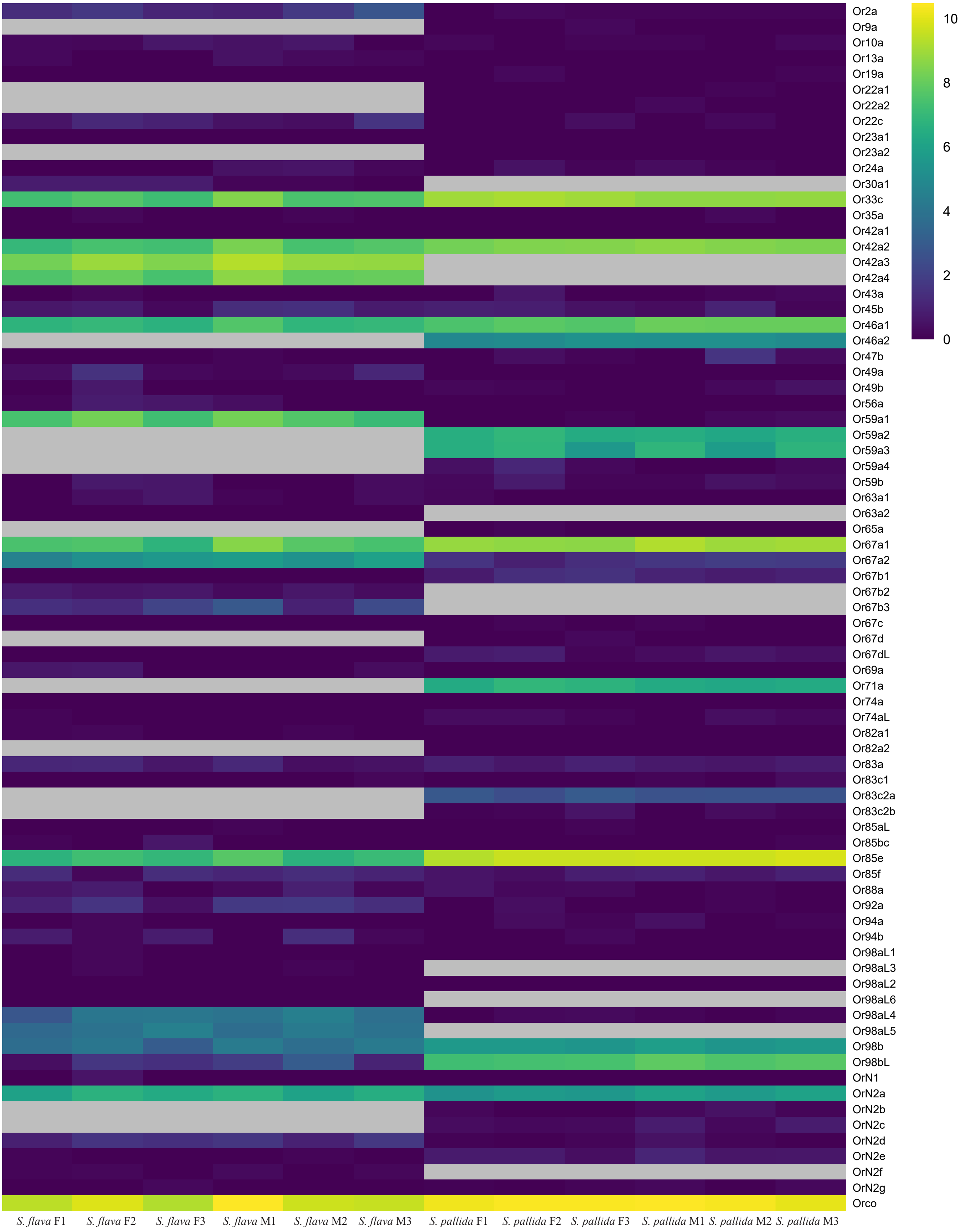

bioRxiv preprint doi: https://doi.org/10.1101/2024.10.08.617316; this version posted May 29, 2025. The copyright holder for this preprint (which was not certified by peer review) is the author/funder, who has granted bioRxiv a license to display the preprint in perpetuity. It is made available under aCC-BY 4.0 International license.

Supp. Figure 8

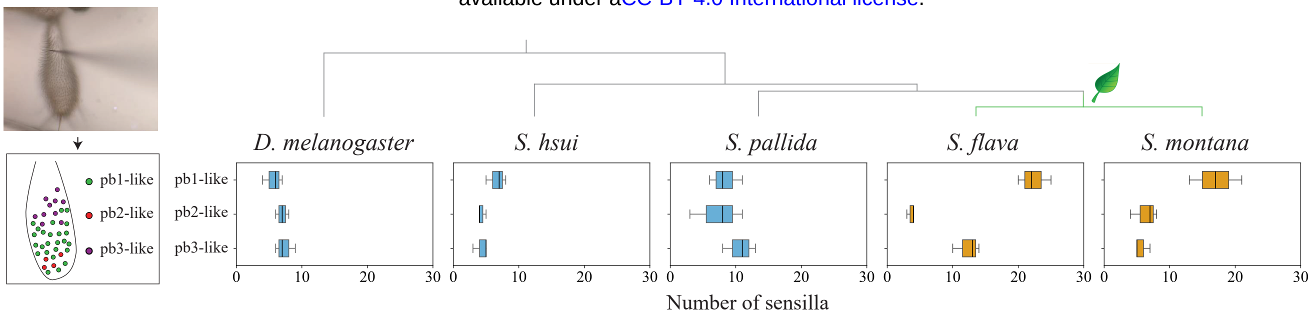

bioRxiv preprint doi: https://doi.org/10.1101/2024.10.08.617316; this version posted May 29, 2025. The copyright holder for this preprint (which was not certified by peer review) is the author/funder, who has granted bioRxiv a license to display the preprint in perpetuity. It is made available under aCC-BY 4.0 International license.

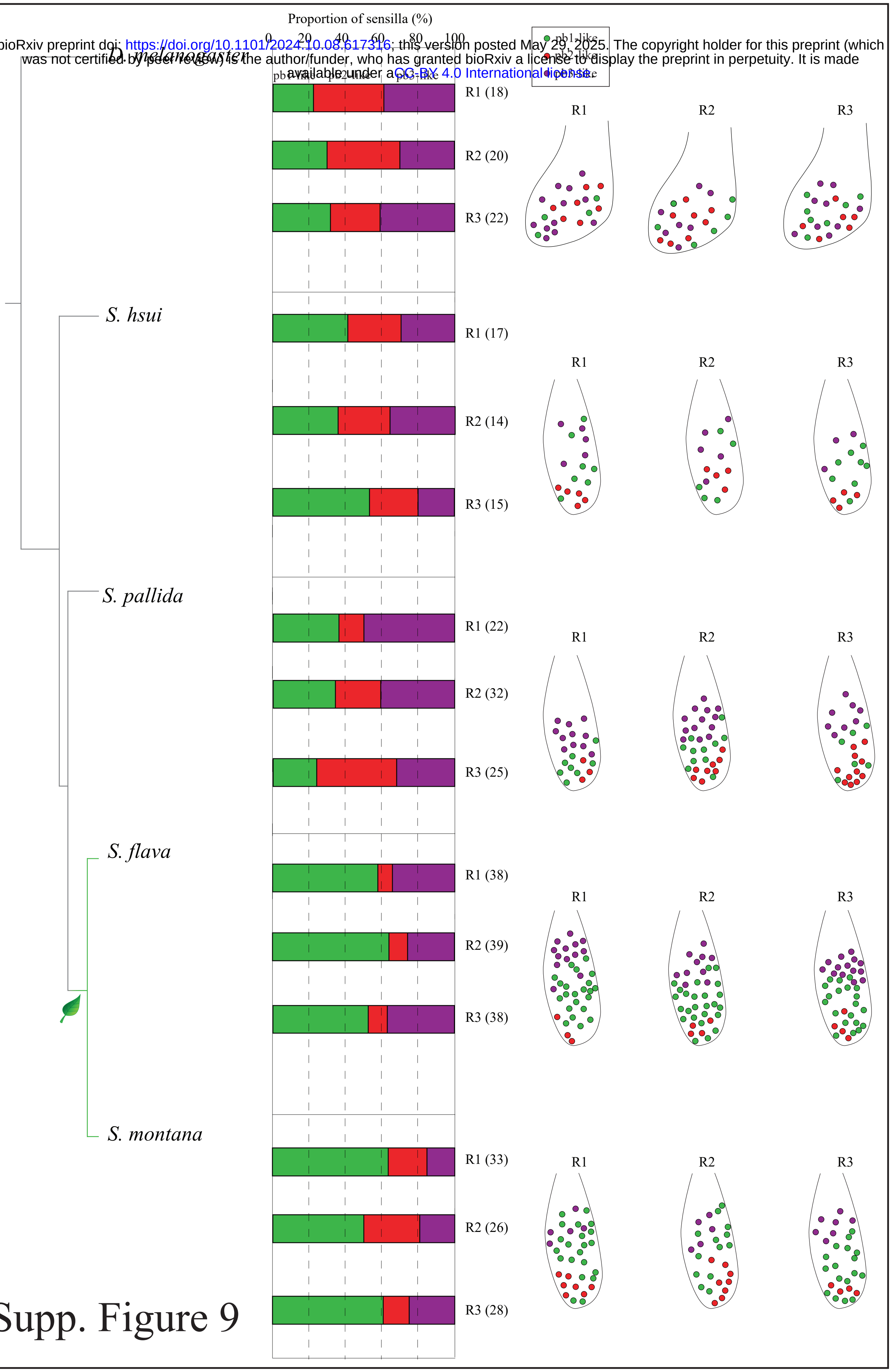

Supp. Figure 9

bioRxiv preprint doi: https://doi.org/10.1101/2024.10.08.617316; this version posted May 29, 2025. The copyright holder for this preprint (which was not certified by peer review) is the author/funder, who has granted bioRxiv a license to display the preprint in perpetuity. It is made available under aCC-BY 4.0 International license.

Supp. Figure 10

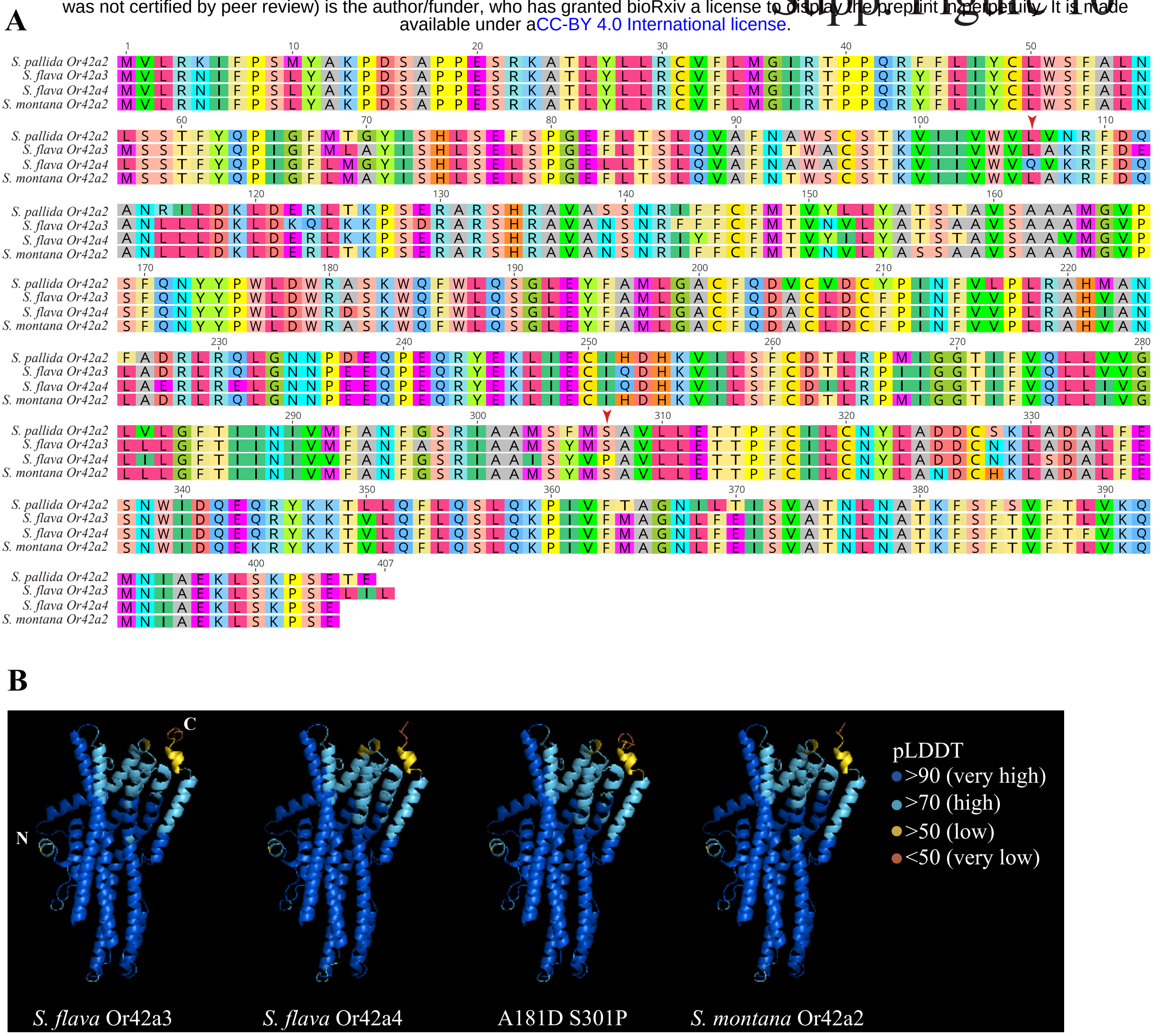

bioRxiv preprint doi: https://doi.org/10.1101/2024.10.08.617316; this version posted ... 2025. The copyright holder for this preprint (which was not certified by peer review) is the author/funder, who has granted bioRxiv a license to display the preprint in perpetuity. It is made available under a CC-BY 4.0 International license.

Supp. Figure 11

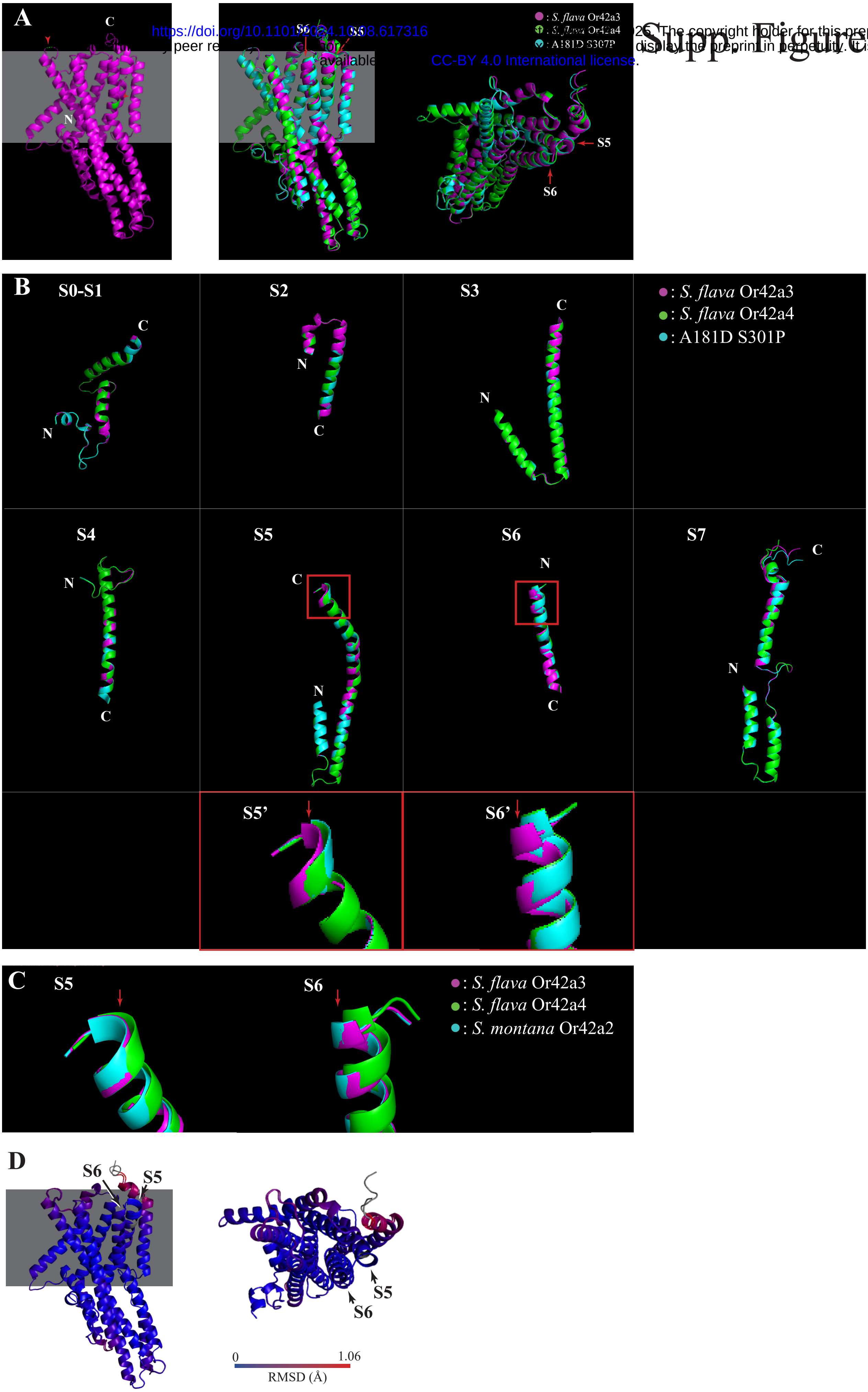

bioRxiv preprint doi: https://doi.org/10.1101/2024.10.08.617316; this version posted May 29, 2025. The copyright holder for this preprint (which was not certified by peer review) is the author/funder, who has granted bioRxiv a license to display the preprint in perpetuity. It is made available under aCC-BY 4.0 International license.

Supp. Figure 12

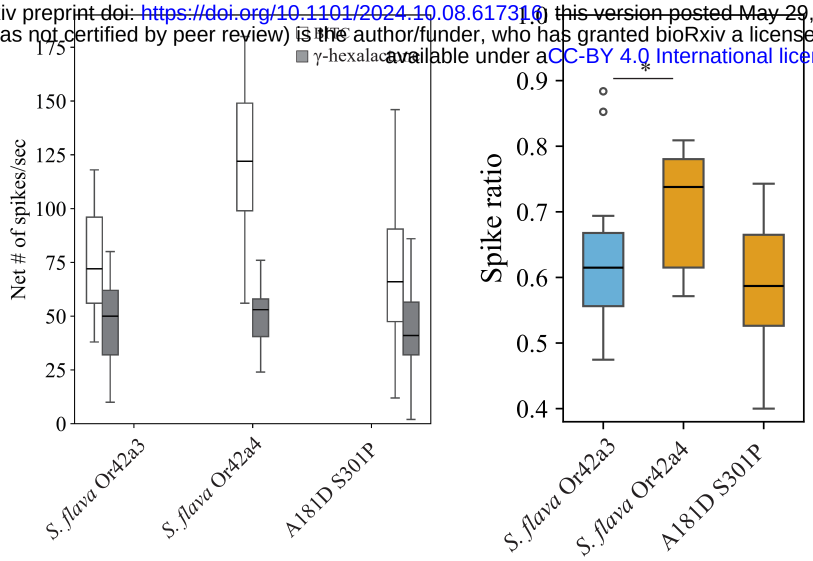

bioRxiv preprint doi: https://doi.org/10.1101/2024.10.08.617316; this version posted May 29, 2025. The copyright holder for this preprint (which was not certified by peer review) is the author/funder, who has granted bioRxiv a license to display the preprint in perpetuity. It is made available under aCC-BY 4.0 International license.

Supp. Figure 13

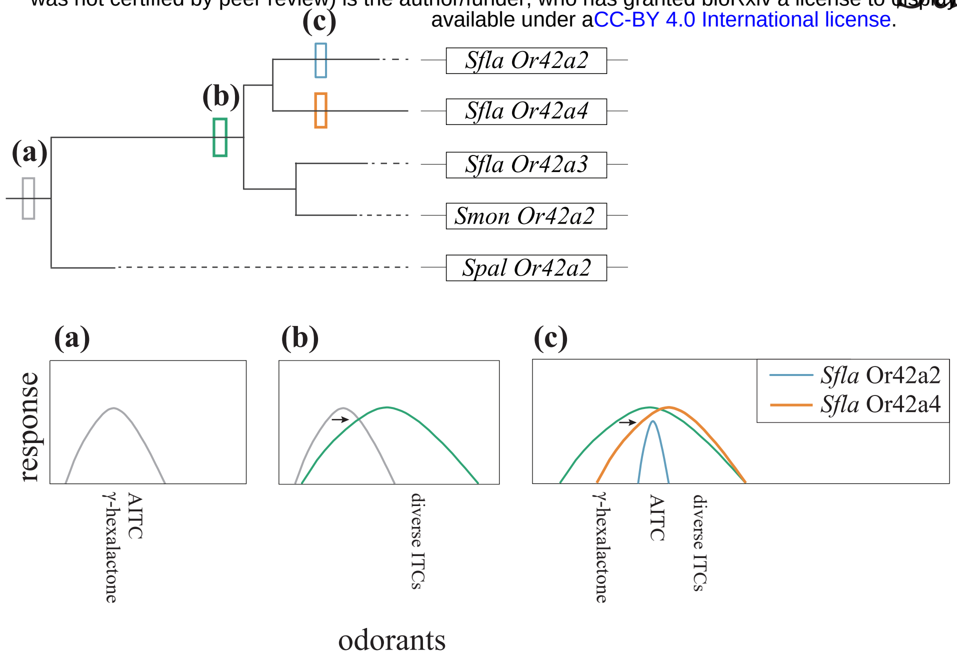

Supplement: Supplement 13 — Figure Supplement 1: Schematic representations of the behavioral assays. (A) Mobility assay used as a proxy for intoxication. Mated female flies (n=10) were placed in the upper chamber (lateral view) and monitored every 10 minutes in presence of volatile chemicals (4 drops of a 5 μl solution in the lower chamber, top view). The top and the bottom chambers were separated by a fabric mesh, preventing direct contact between the flies and the chemical solutions. (B) Positional olfactory assay. Groups of 10–12 non-starved cold-anesthetized mated females were placed in a small piece of clear Tygon tube, which was then used to connect the narrow ends of two cut glass Pasteur pipettes (non-choice/release zone). The distal end of each glass pipette was connected to a glass vial containing 10 μL of the odor solution or the solvent control; a piece of fabric mesh prevented insects from entering the vials containing the odor or control solutions. The number of flies in each of the two tubes, as well as in the middle release section, were counted every five minutes until minute 35, and in tests with allyl ITC, again at 65 minutes. The % of insects that choose one or the other tube over the total number of insects released, and the % of insects in the tube closest to the odor source over the total number of insects that choose one or the other tube, was then calculated for each time point. (C) Feeding assay in presence of odors or the solvent control. In each test a group of 24 hours wet-starved mated female flies (n=11–15) was placed in a vial containing a piece of filter paper impregnated with 160 μl of 50 mM D-glucose dyed blue. A small container with a mesh bottom was affixed to the inner side of the vial cap contained a piece of filter paper loaded with 10 μl of an odorant solution or the solvent control, such as that flies could smell but not contact the odor source. After 15 minutes, vials were frozen for at least 60 minutes, the amount of blue dye in the abdomen of each f [file NIHPP2024.10.08.617316v3-supplement-13.pdf]
