# Supplementary material for: Odorant receptors tuned to isothiocyanates in Drosophila melanogaster are co-opted and expanded in herbivorous relatives
Source: bioRxiv. 2025 Mar 10:2024.10.08.617316. Originally published 2024 Oct 11. Preprint. [Version 2] doi: 10.1101/2024.10.08.617316 (PMC11482750; doi:10.1101/2024.10.08.617316)

bioRxiv preprint doi: https://doi.org/10.1101/2024.10.08.617316; this version posted March 11, 2025. The copyright holder for this preprint (which was not certified by peer review) is the author/funder, who has granted bioRxiv a license to display the preprint in perpetuity. It is made available under aCC-BY 4.0 International license.

FIGURE SUPPLEMENT CAPTIONS:

Figure Supplement 1: Schematic representations of behavioral assays. (A) Mobility assay used as a proxy for intoxication. Mated female flies (n=10) were placed in the upper chamber and monitored every 10 minutes in presence of volatile chemicals (4 drops of a 5 µl solution in the lower chamber). The top and the bottom chambers were separated by a fabric mesh, preventing direct contact between the flies and the chemical solutions. **(B)** Feeding assay in presence of odors. A group of 24 hours wet-starved mated female flies (n=11-15) was transferred to a vial containing a piece of filter paper impregnated with 160 µl of 50 mM D-glucose dyed blue. A small container affixed to the flug vial had a piece of filter paper loaded with 10 µl of an odorant solution or the solvent control, and because the opening was enclosed by mesh, flies could smell but not contact the odor source. After 15 minutes, vials

bioRxiv preprint doi: https://doi.org/10.1101/2024.10.08.617316; this version posted March 11, 2025. The copyright holder for this preprint (which was not certified by peer review) is the author/funder, who has granted bioRxiv a license to display the preprint in perpetuity. It is made available under aCC-BY 4.0 International license.

were frozen for at least 60 minutes, the amount of blue dye in the abdomen of each fly in a vial was quantified blind to treatment, and a feeding score was calculated for each vial as explained in materials and methods. **(C)** Positional olfactory assay. Groups of 10-12 non-starved mated females were cold-anesthetized in a small piece of clear Tygon tube, which was then used to connect the narrow ends of two cut glass Pasteur pipettes. The distal end of each glass pipette was connected (via a piece of Tygon tube) to a 1.75 mL glass vial containing 10 µL of the odor solution or the solvent control; a piece of fabric mesh prevented insects from entering the vials containing the odor or control solutions. The number of flies in each of the two tubes, as well as in the middle "release" section, were counted every five minutes until minute 35, and in tests with AITC, again at 65 minutes. The % of insects that choose one or the other tube over the total number of insects released, and the % of insects in the tube closest to the odor source over the total number of insects that choose one or the other tube, was then calculated for each time point.

Figure Supplement 2. Behavioral data related to Figure 1D-F, and effect of γ-hexalactone in olfactory behavior and mobility. (A) Feeding assay, as in Figure 1E, but using γ-hexalactone (1:50 vol/vol). The dotted line at 50% indicates neither feeding enhancement or repellence. Canton-S flies consumed more in presence than in absence of γ-hexalactone (one-sample signed rank test against expected median=1, $p<0.001$, n=25), but *Or42a-/-* mutants consumed less in presence of the odor; $p<0.001$, N=24). The asterisks indicate significant differences between genotypes (***$p<0.001$, Mann-Whitney U test). **B-H:** Positional olfactory assays. **(B-C)** Tests with AITC 1:500 vol/vol vs mineral oil (as in Figure 1F) using Canton-S flies (n=19); flies preferred the odorless tube from minute 25 onwards (**B**, one sample signed rank tests, *$p<0.05$ in all cases). **(C-D)** % of flies that choose either tube in tests with AITC, as a proxy for activity [calculated as: (# of flies in one or the other tube / # of flies released) *100]; **C:** Canton-S flies, n=19; **D:** genetic control and *Or42a-/-* mutants, n=15 for each genotype. **(E-F)** Tests using γ-hexalactone 1:10 vol/vol vs DMSO. **E:** % of genetic control and *Or42a-/-* flies in the odorous tube (*$p<0.05$, one-sample signed rank tests against median=50%, n=19 for each genotype). **F:** % of genetic control and *Or42a-/-* that choose either tube. Mann-Whitney U tests comparing the two groups at each time point, *$p<0.05$, ***$p<0.005$. **(G-H)** Tests with *Or7a-/-* (n=15) and genetic control flies (n=18) using AITC 1:500 vol/vol vs mineral oil. **G:** % of flies in the odorous tube (*$p<0.05$, ***$p<0.001$, one sample signed rank tests). Differences between genotypes were significant at all time

bioRxiv preprint doi: https://doi.org/10.1101/2024.10.08.617316; this version posted March 11, 2025. The copyright holder for this preprint (which was not certified by peer review) is the author/funder, who has granted bioRxiv a license to display the preprint in perpetuity. It is made available under aCC-BY 4.0 International license.

points except 5 minutes (Mann-Whitney U tests). Because both genotypes were assayed in parallel, control data is different from that presented in Figure 1F and panel D. **H:** % of flies that choose either tube. **(I-J)** Tests with *Or35a-/-* (n=29) and genetic control flies (n=27) using γ-hexalactone 1:10 vol/vol vs DMSO. **I:** % of flies in the odorous tube (*p<0.05, ***p<0.001, one sample signed rank tests). Differences between genotypes were significant at all time points except 5 minutes (Mann-Whitney U tests, p<0.05-0.001 in all cases). **J:** % of flies that choose either tube. Because both genotypes were assayed in parallel, control data is different from that presented in panels E-F. **(K)** Mobility assay, as in Figure 1A, but using γ-hexalactone at various concentrations from 1:500 to 1:2.5 vol/vol (n=). Flies remained active in presence of this volatile. **(L)** SSR from all *Dmel* antennae (ab) and palp basiconic (pb) sensilla to stimulation with γ-hexalactone 1:100 vol/vol. Only pb1a is activated by γ-hexalactone (n= 3-6 animals/OSNs). **(M)** Activation of Or42a, in combination with distinct olfactory channels, is involved in mediating both olfactory repellence to AITC (light blue arrow) and attraction to γ-hexalactone (orange arrow). Activation of Or42a in combination with Or7a or O35a likely mediates repellence and attraction, respectively.

Figure Supplement 3: Hierarchical cluster analysis based on odorant response profiles across species. **(A)** Hierarchical clusters were constructed using odorant response data from *Dmel, Shsu, Spal, Sfla,* and *Smon* using R studio v1.4.1717. A terminal node corresponds to a single recording from the individual sensilla; n=6-8 sensilla from 3-4 animals. **(B)** Hierarchical clusters were created for each sensilla type, including pb1-like, pb2-like, and pb3-like, using the odorant response (control-subtracted net number of spikes) average from each species as inputs. **(C)** The species phylogeny (left: Kim et al., 2021) and palp functional dendrogram (right) highlight the relevance of niche differences in shaping odorant responses. The palp functional dendrogram was generated using odorant response average for each species.

Figure Supplement 4. Responses of OSNs from *Dmel* pb1a and *Scaptomyza* pb1a-like sensilla to stimulation with various odorant concentrations. **(A)** Responses of pb1a OSNs across species upon stimulation with various concentrations (ranging from $1{:}10^{-2}$ to 10^{-5} vol/vol) of ethyl butyrate, γ-hexalactone, *trans*-2-hexenal, AITC, IBITC, BITC, and SBITC (n=6-8 sensilla from 3-4 animals/species). The chemical structures of each compound are shown on the left. IBITC, BITC, and

bioRxiv preprint doi: https://doi.org/10.1101/2024.10.08.617316; this version posted March 11, 2025. The copyright holder for this preprint (which was not certified by peer review) is the author/funder, who has granted bioRxiv a license to display the preprint in perpetuity. It is made available under aCC-BY 4.0 International license.

SBITC were only tested in *Sfla* and *Smon* pb1a, as BITC at 10^{-2} vol/vol failed to activate OSNs in pb1a from microbe-feeding species (Figure 2). **(B-C)** Responses of *Sfla* and *Smon* pb1a OSNs upon stimulation with γ-hexalactone 10^{-4} vol/vol **(B)** and *trans*-2-hexenal 10^{-4} vol/vol **(C)**. n=6-7 sensilla from 3 animals/species. Mann-Whitney U tests: *$p<0.05$, **$p<0.01$. **(D)** Half maximal effective concentrations (EC_{50}) for each fly species and odorant (see Methods for calculations of EC_{50}). Kruskal-Wallis tests followed by Dunn's multiple comparisons. Different letters indicate significant differences (p-values for ethyl butyrate: *Spal* vs *Sfla* $p<0.05$, *Spal* vs *Smon* $p<0.01$; for γ-hexalactone: $p<0.001$; for *trans*-2-hexenal: *Dmel* vs *Smon* $p<0.05$, *Shsu* vs *Sfla* $p<0.01$, *Dmel* vs *Sfla* $p<0.0001$; for AITC: $p<0.05$). Overall, pb1a OSNs of microbe-feeding species exhibited greater sensitivity to γ-hexalactone, whereas Brassicales specialists showed higher sensitivity to *trans*-2-hexenal and AITC. *Sfla* and *Smon* showed similar sensitivities to all four ITCs tested.

Figure Supplement 5: Location and percentages of the three different maxillary palp basiconic (pb) sensilla across species. Classification of maxillary palp pb sensilla using SSRs and stimulation with diagnostic odorants. Data represents the proportion of pb1 and pb1-like (green), pb2 and pb2-like (red), and pb3 and pb3-like (magenta) in *Dmel, Shsu, Spal, Sfla,* and *Smon*. The schematic positions of pb sensilla in each of the three replicates (R1, R2, and R3) from each species are shown on the right. The total number of sensilla identified in each animal is indicated between parentheses.

Figure Supplement 6: Maxillary palp and body area measurements across species. (A) A representative photograph and measurement of a male *Sfla* viewed under a conventional dissection microscope. The areas of the maxillary palp and of the body are respectively indicated by the broken magenta and green lines. Body size was defined as the sum of the head, thorax (excluding legs and wings) and abdomen areas. **(B)** Data represents the palp (top) and body size (middle) areas, along with their ratio (bottom) in *Dmel, Shsu, Spal*, and *Sfla*.

Figure Supplement 7: Or42a gene tree constructed by RAxML. Maximum-likelihood gene tree with 1000 bootstrap cycles using *Shsu Or42a1*, *Spal Or42a1*, *Sgra Or42a1*, *Smon Or42a1*, *Sfla Or42a1*, *Shsu Or42a2*, *SpalOr42a2*, *Sgra Or42a2*, *Sfla Or42a2*, *Smon Or42a*, *Sfla Or42a2*, *Sfla Or42a3*, *Sfla Or42a4*, and *Dmel Or42a* as an outgroup, generated by RAxML (v8.2.19) (Stamatakis 2014) after alignment by

bioRxiv preprint doi: https://doi.org/10.1101/2024.10.08.617316; this version posted March 11, 2025. The copyright holder for this preprint (which was not certified by peer review) is the author/funder, who has granted bioRxiv a license to display the preprint in perpetuity. It is made available under aCC-BY 4.0 International license.

MAFFT v7 (Katoh and Standley 2013). Bootstrap values for the gene trees are denoted only when the values were smaller than 80. The tree was displayed by FigTree v1.4.3 (Rambaut 2009)

Figure Supplement 8: Maxillary palp RNA seq of *Spal* and *Sfla Or*s. The heatmap displays Log2[RPM+1] values of *Or*s expression in the maxillary palps of female (F) and male (M) *Sfla* and *Spal* (n=3/sex). Each column represents a replicate (e.g. *Sfla* F1: replicate #1 of female *Sfla*, *Sfla* M1: replicate #1 of male *Sfla*). Gray boxes indicate that the corresponding genes were unidentified in the genomes of the species.

Figure Supplement 9: Alignment of 1D and 3D structures of amino acids of Or42a. (A) The 1D alignment of amino acids of *Spal* Or42a2, *Sfla* Or42a3, and *Sfla* Or42a4, and *Smon* Or42a2. Red arrowheads denote the sites where site-directed-mutagenesis was performed to generate the A181D S301P chimera. **(B)** The 3D structures of *Sfla* Or42a3, *Sfla* Or42a4, A181D S301P, *Smon* Or42a2 were predicted using AlphaFold2. The colors of the amino acids indicate the predicted local distance difference test (pLDDT) scores: blue (>90), cyan (>70), yellow (> 50), and orange (<50).

Figure Supplement 10: Screening of candidate amino acids by AlphaFold2 3D prediction. (A) Lateral view of the 3D structure of A181D S301P predicted by AlphaFold2 (left). *Sfla* Or42a3-derived amino acids are indicated in magenta, and the substitutions with *Sfla* Or42a4-derived amino acids are represented in green. The middle and right panels respectively display side and top views of alignment of the predicted structures of *Sfla* Or42a3, *Sfla* Or42a4, and A181D S301P. The extracellular region at S5 and S6 (red arrows) highlights the local structural differences between *Sfla* Or42a3 and Or42a4 where the A181D, S301P overlaps with the local structure of *Sfla* Or42a4. **(B)** The 3D predictions of *Sfla* Or42a3 (magenta), *Sfla* Or42a4 (green), and A181D S301P (cyan) were aligned using PyMol 2.5.3. Red rectangles highlight the sites of local structural variance between *Sfla* Or42a3 and Or42a4. The A181D S301P shows a closer alignment with Or42a4. S5' and S6' are enlarged views of S5 and S6, respectively. **(C)** The enlarged views of S5 and S6 in the alignment of 3D predictions of *Sfla* Or42a3 (magenta), *Sfla* Or42a4 (green), and *Smon* Or42a2 (cyan). **(D)** The 3D alignment of *Sfla* Or42a3 and *Smon* Or42a2 predicted by AlphaFold2. The root mean square deviation (RMSD) is visualized with a color gradient.

bioRxiv preprint doi: https://doi.org/10.1101/2024.10.08.617316; this version posted March 11, 2025. The copyright holder for this preprint (which was not certified by peer review) is the author/funder, who has granted bioRxiv a license to display the preprint in perpetuity. It is made available under aCC-BY 4.0 International license.

Figure Supplement 11: Responses of OSNs expressing homozygous Or42a to stimulation with BITC and γ-hexalactone. Left: responses of OSNs expressing homozygous *Sfla* Or42a3, *Sfla* Or42a4, or A181D S307P (*UAS-Or42a; Or67d* ^{Gal4}) to stimulation with BITC (white) and γ-hexalactone (grey) (n=16-30 from 5-10 animals). Right: ratio between responses to BITC and the sum of the responses evoked by stimulation with BITC and γ-hexalactone. Kruskal-Wallis followed by Dunn's multiple comparisons, *p<0.05.

Figure Supplement 12: A model for the evolution of Or42a in *Sfla, Smon* and *Spal*. The evolution of Or42a begins with a shift in the ligand specificity of an ancestral Or42a (a), which was tuned to fruit-borne odors such as γ-hexalactone and limited ITCs (i.e. AITC), and later broadened to detect a wider range of ITCs (b). Subsequent gene triplication of *Sfla Or42a* resulted in three paralogous genes (c: *Sfla Or42a2, Sfla Or42a3,* and *Sfla Or42a4*). The lineage leading to *Sfla* Or42a2 and *Sfla* Or42a4 experienced a reduction in sensitivity to γ-hexalactone, while both *Sfla* Or42a3 and *Smon* Or42a2 retained sensitivity to γ-hexalactone.

SUPPLEMENTARY MATERIAL AND METHODS:

Measurement of maxillary palps and body size

Two-to-ten-days old male *Dmel, Shsu, Spal,* and *Sfla* flies with their heads facing up and their bodies sideway were mounted and imaged using a commercial camera under a conventional microscope. The areas of the maxillary palps and the body (head, thorax excluding legs and wings, and abdomen) were measured using ImageJ v1.54.

Dilution series

Trans-2-hexenal, AITC, IBITC, SBITC, and ethyl butyrate were diluted in mineral oil, and γ-hexalactone was diluted in DMSO to 10^{-5} to 10^{-2} vol/vol. Half maximal effective concentration (EC50) was generated by GraphPad Prism v10.2.1.

bioRxiv preprint doi: https://doi.org/10.1101/2024.10.08.617316; this version posted March 11, 2025. The copyright holder for this preprint (which was not certified by peer review) is the author/funder, who has granted bioRxiv a license to display the preprint in perpetuity. It is made available under aCC-BY 4.0 International license.

Construction of Or42a gene tree

The amino acid sequences of *Dmel Or42a,* and *Sfla Or42a1, Sfla Or42a2, Sfla Or42a3,* and *Sfla Or42a4* were obtained from Flybase (2024_01), and Goldman-Huertas et al., 2015. Blast searches were conducted to annotate *Shsu Or42a1, Spal Or42a1, Sgra Or42a1, Smon Or42a1, Sfla Or42a1, Shsu Or42a2, Spal Or42a2, Sgra Or42a2,* and *Smon Or42a2*, using *Sfla Or42a1* and *Sfla Or42a2* as query against the genomes of *Shsu* (ASM1815282v1), *Spal* (ASM1815296v1), *S. graminum* (ASM1890183v1), and *Smon* (ASM1890430v1). Amino acid sequences of *Shsu Or42a1, Spal Or42a1, Sgra Or42a1, Smon Or42a1, Sfla Or42a1, Shsu Or42a2, SpalOr4* (Katoh and Standley 2013)*2a2, Sgra Or42a2, Sfla Or42a2, Smon Or42a, Sfla Or42a2, Sfla Or42a3, Sfla Or42a4,* and *Dmel Or42a* were aligned by MAFFT v7.511 with JTT as scoring matrix for amino acid sequences with L-INS-i strategy. Maximum-likelihood gene tree with 1000 bootstrap cycles using the above sequences were constructed with RAxML (v8.2.19)(Stamatakis 2014). GAMMA model of rate heterogeneity was applied. CDS and alignment of *Or42a* are included in Supplementary files 12 and 13, respectively.

bioRxiv preprint doi: https://doi.org/10.1101/2024.10.08.617316; this version posted March 11, 2025. The copyright holder for this preprint (which was not certified by peer review) is the author/funder, who has granted bioRxiv a license to display the preprint in perpetuity. It is made available under aCC-BY 4.0 International license.

Supp. Figure 1

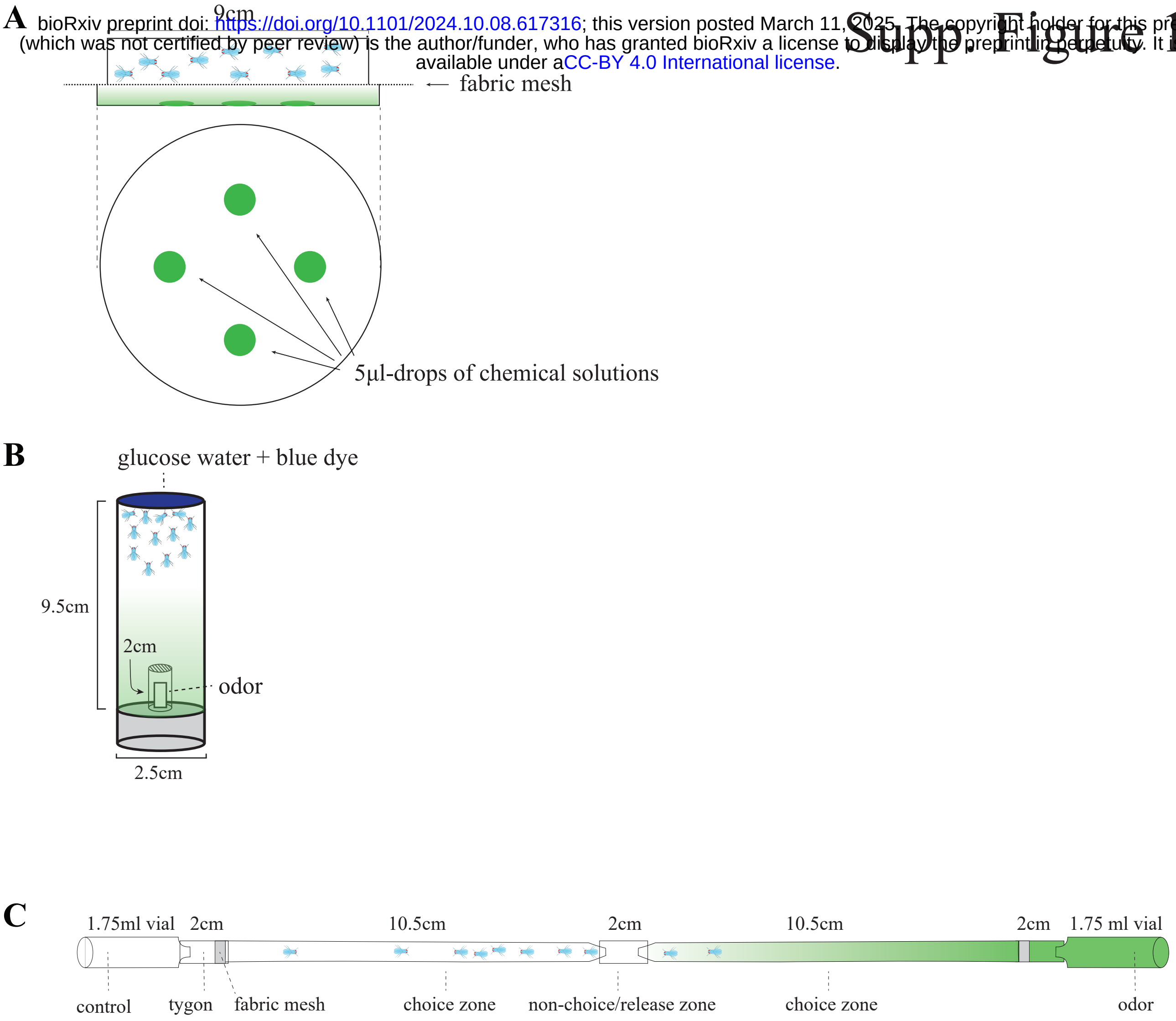

bioRxiv preprint doi: https://doi.org/10.1101/2024.10.08.617316; this version posted March 11, 2025. The copyright holder for this preprint (which was not certified by peer review) is the author/funder, who has granted bioRxiv a license to display the preprint in perpetuity. It is made available under aCC-BY 4.0 International license.

Supp.Figure 2

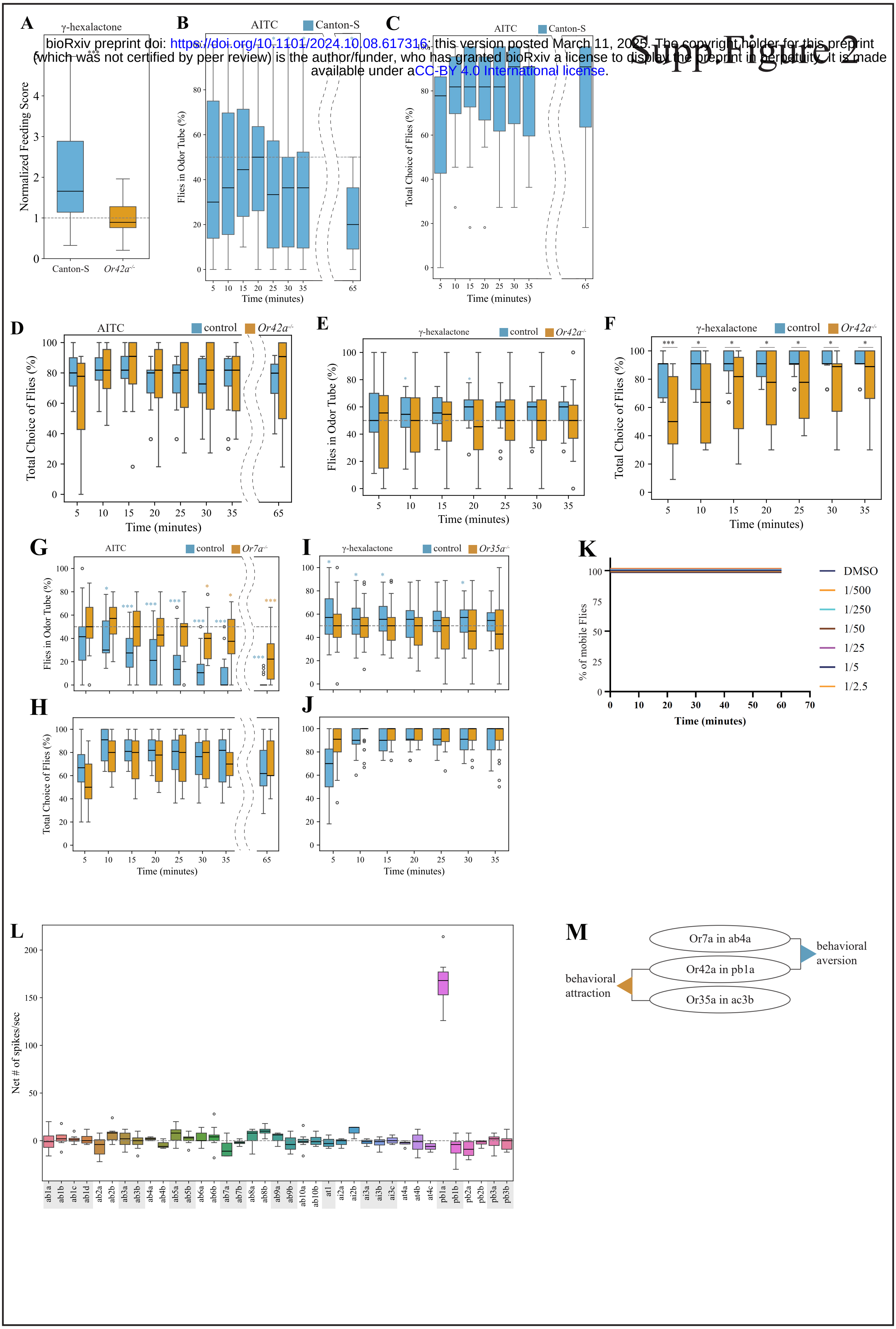

bioRxiv preprint doi: https://doi.org/10.1101/2024.10.08.617316; this version posted March 11, 2025. The copyright holder for this preprint (which was not certified by peer review) is the author/funder, who has granted bioRxiv a license to display the preprint in perpetuity. It is made available under aCC-BY 4.0 International license.

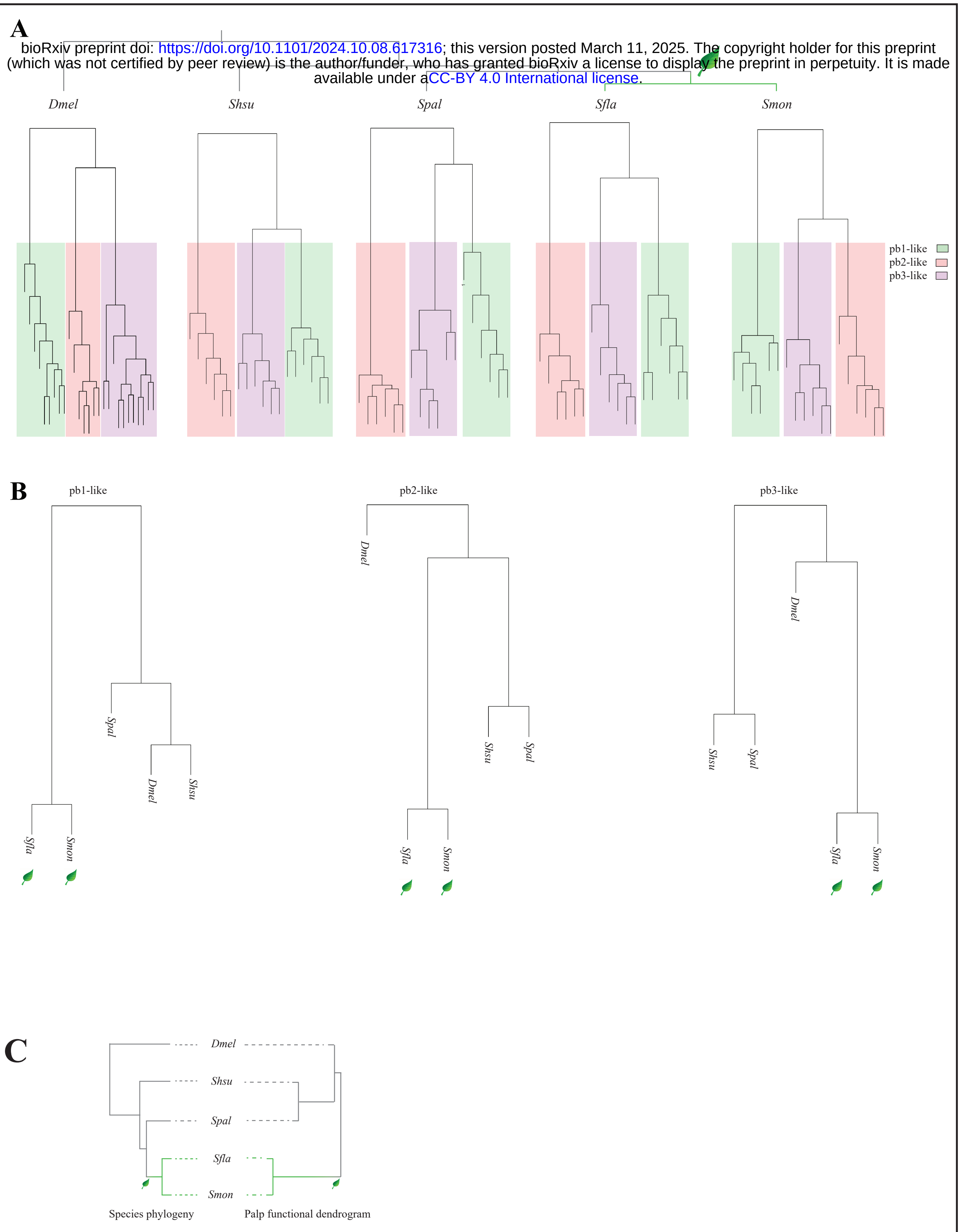

Supp. Figure 3

bioRxiv preprint doi: https://doi.org/10.1101/2024.10.08.617316; this version posted March 11, 2025. The copyright holder for this preprint (which was not certified by peer review) is the author/funder, who has granted bioRxiv a license to display the preprint in perpetuity. It is made available under aCC-BY 4.0 International license.

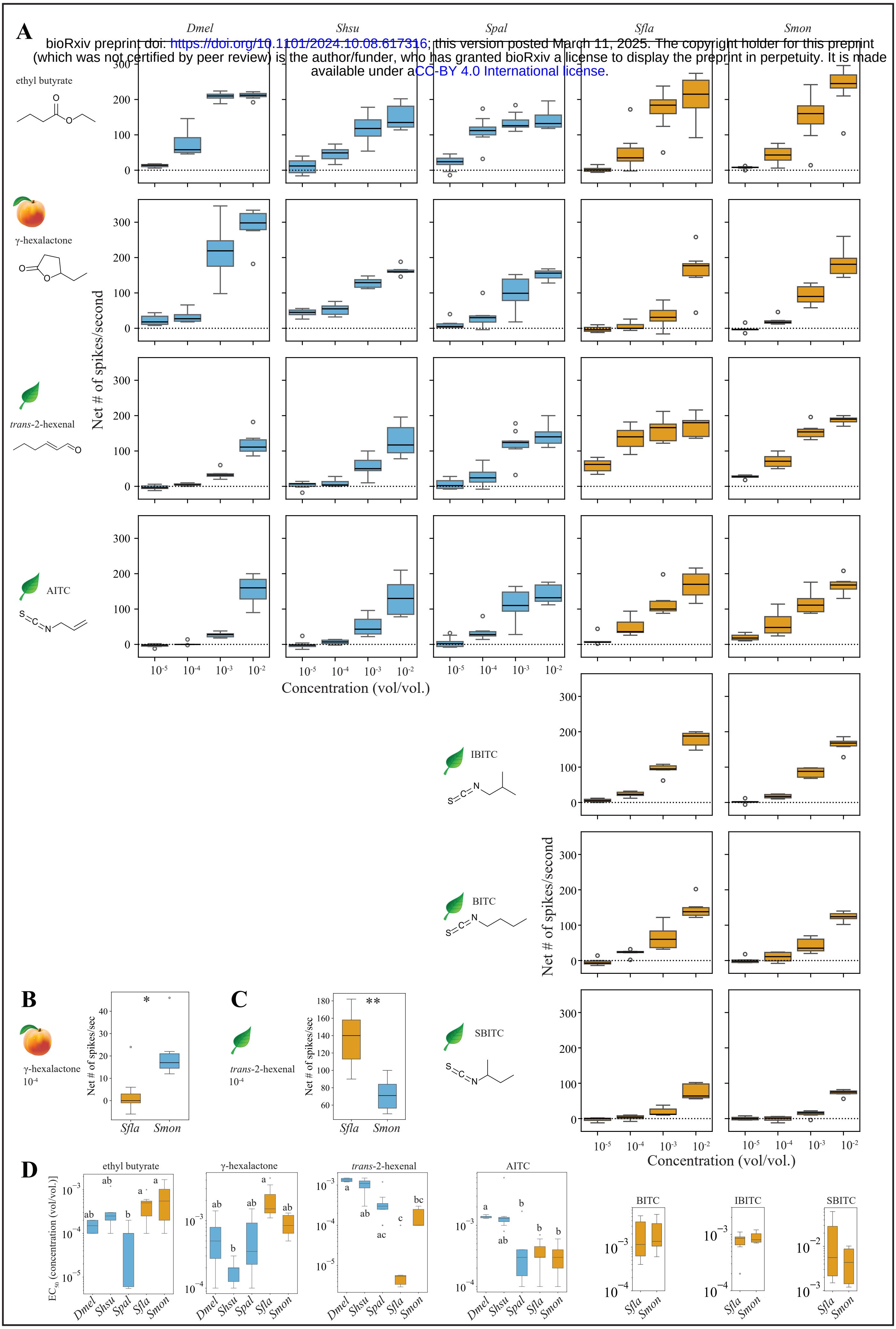

bioRxiv preprint doi: https://doi.org/10.1101/2024.10.08.617316; this version posted March 11, 2025. The copyright holder for this preprint (which was not certified by peer review) is the author/funder, who has granted bioRxiv a license to display the preprint in perpetuity. It is made available under aCC-BY 4.0 International license.

Supp. Figure 5

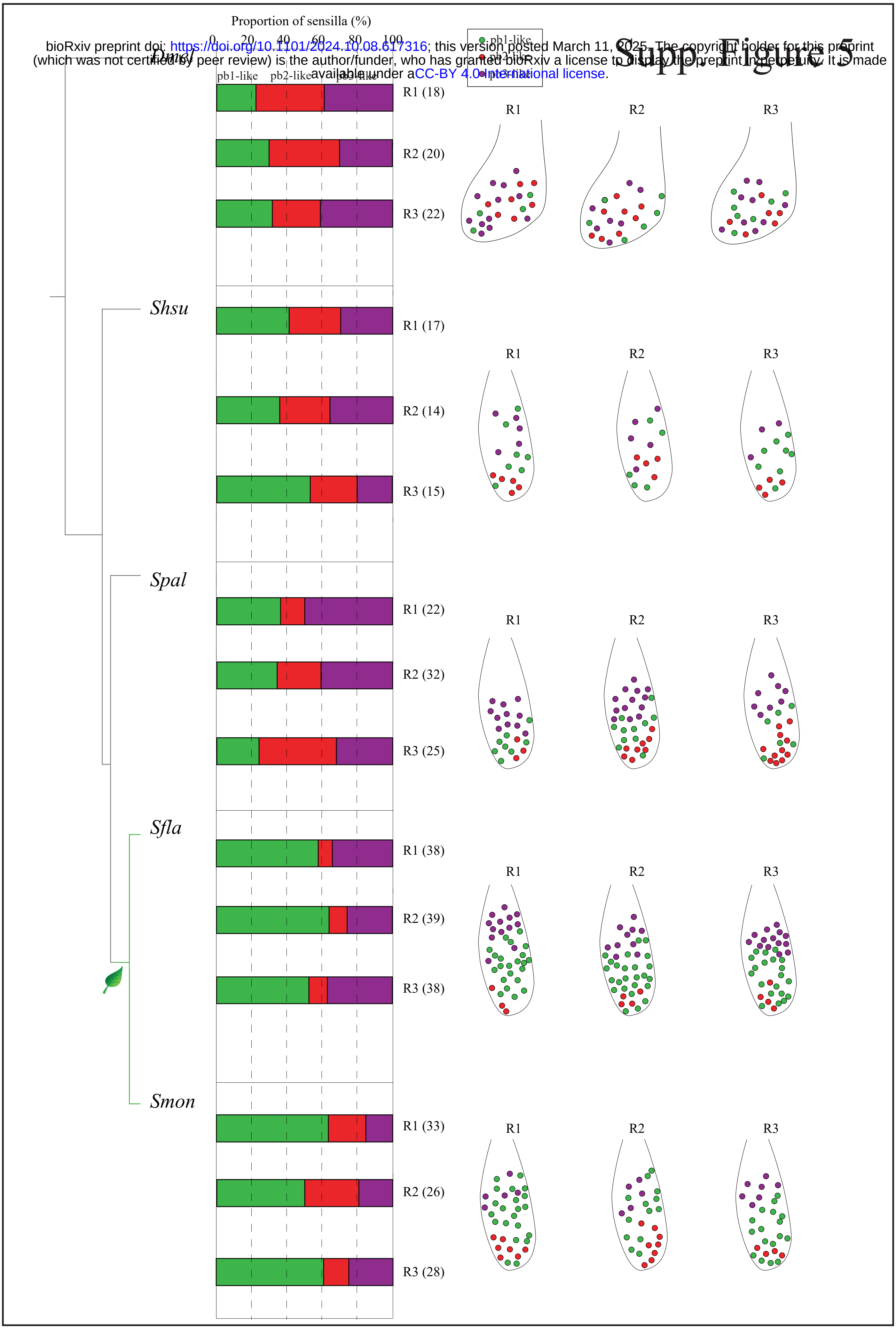

bioRxiv preprint doi: https://doi.org/10.1101/2024.10.08.617316; this version posted March 11, 2025. The copyright holder for this preprint (which was not certified by peer review) is the author/funder, who has granted bioRxiv a license to display the preprint in perpetuity. It is made available under aCC-BY 4.0 International license.

Supp. Figure 6

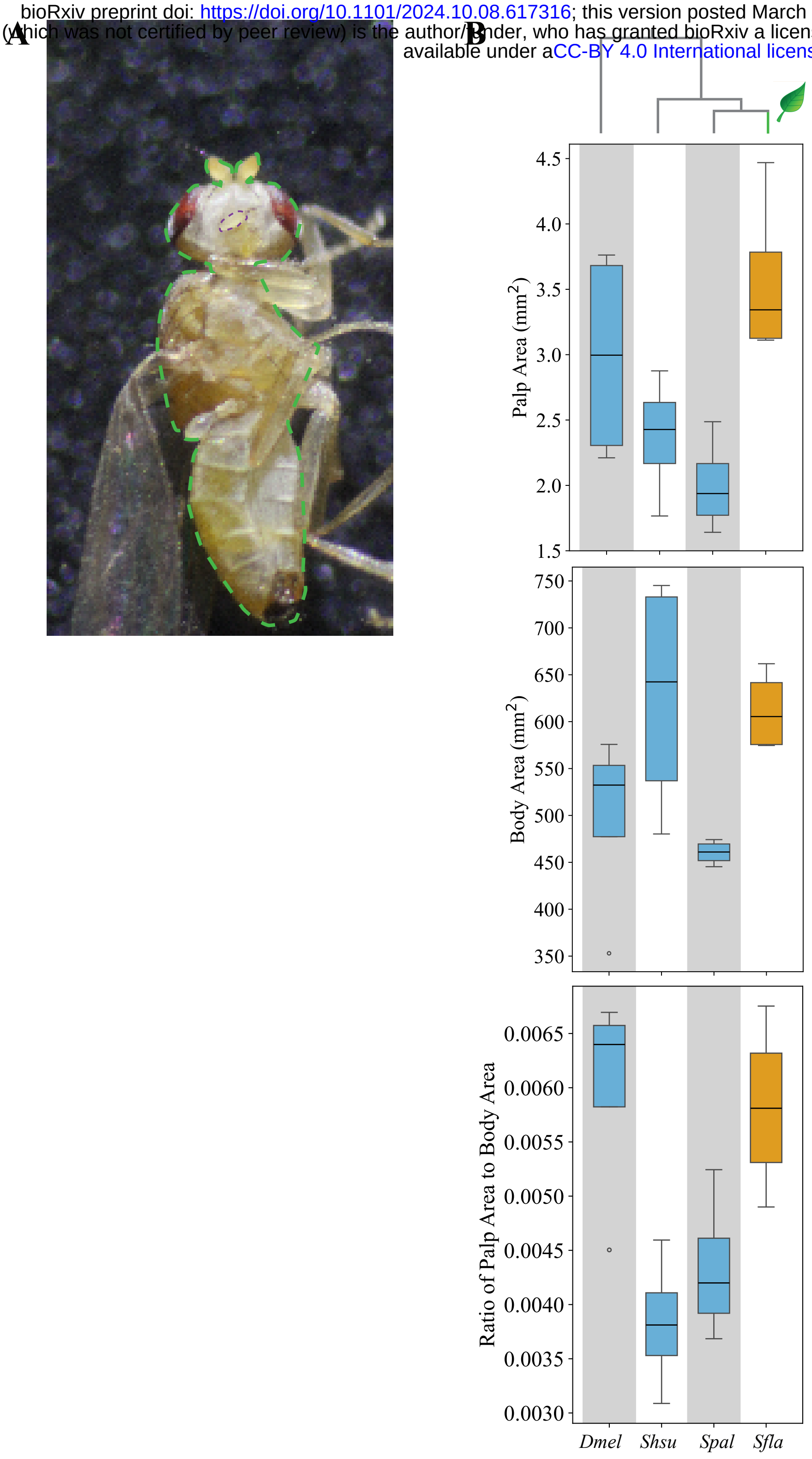

bioRxiv preprint doi: https://doi.org/10.1101/2024.10.08.617316; this version posted March 19, 2025. The copyright holder for this preprint (which was not certified by peer review) is the author/funder, who has granted bioRxiv a license to display the preprint in perpetuity. It is made available under aCC-BY 4.0 International license.

Supp. Figure 7

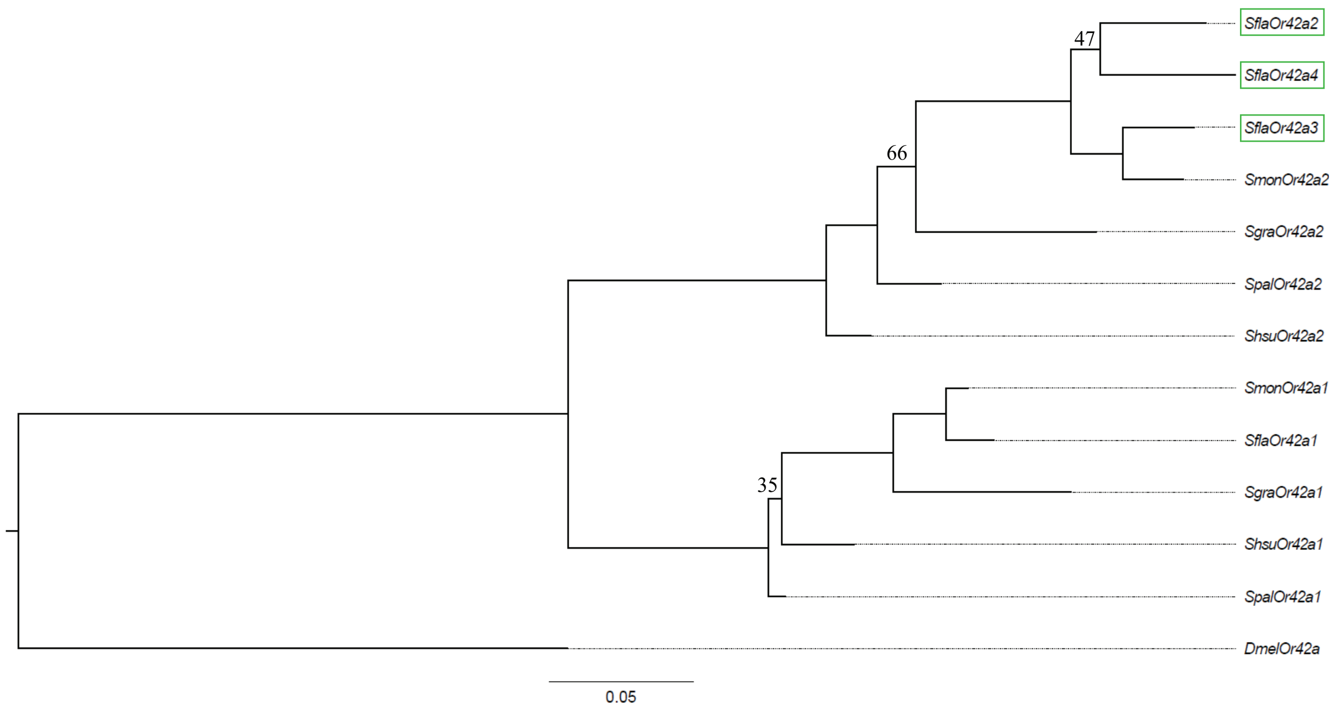

bioRxiv preprint doi: https://doi.org/10.1101/2024.10.08.617316; this version posted March 11, 2025. The copyright holder for this preprint (which was not certified by peer review) is the author/funder, who has granted bioRxiv a license to display the preprint in perpetuity. It is made available under aCC-BY 4.0 International license.

Supp. Figure 8

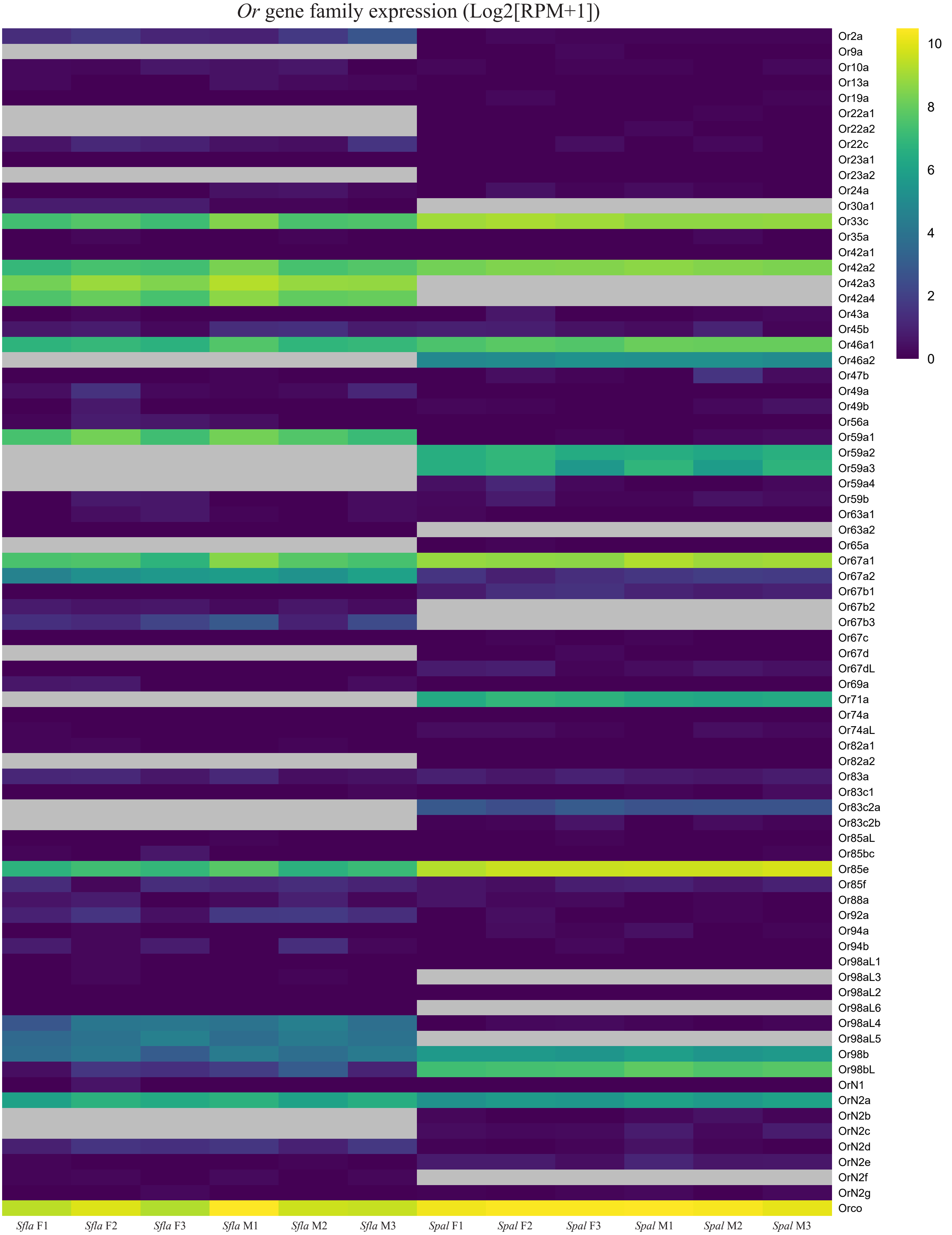

bioRxiv preprint doi: https://doi.org/10.1101/2024.10.08.617316; this version posted March 11, 2025. The copyright holder for this preprint (which was not certified by peer review) is the author/funder, who has granted bioRxiv a license to display the preprint in perpetuity. It is made available under aCC-BY 4.0 International license.

Sup. Figure 9

A

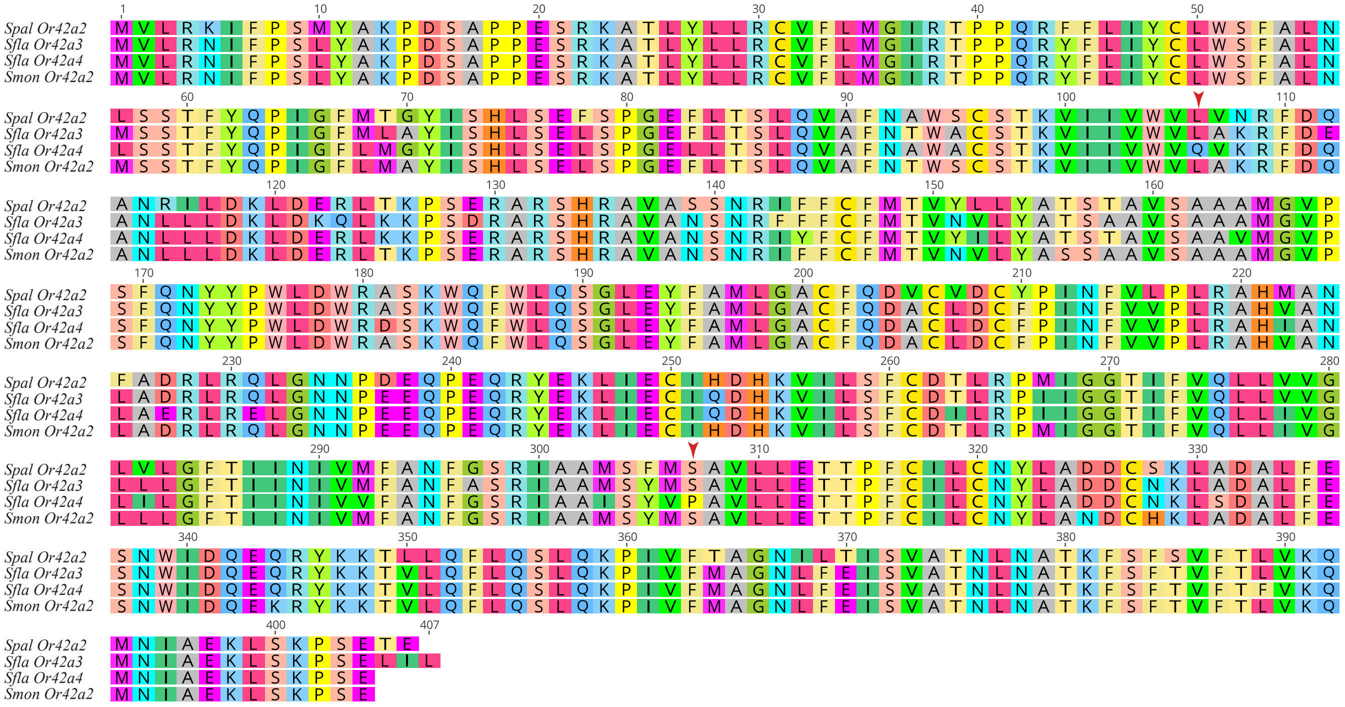

B

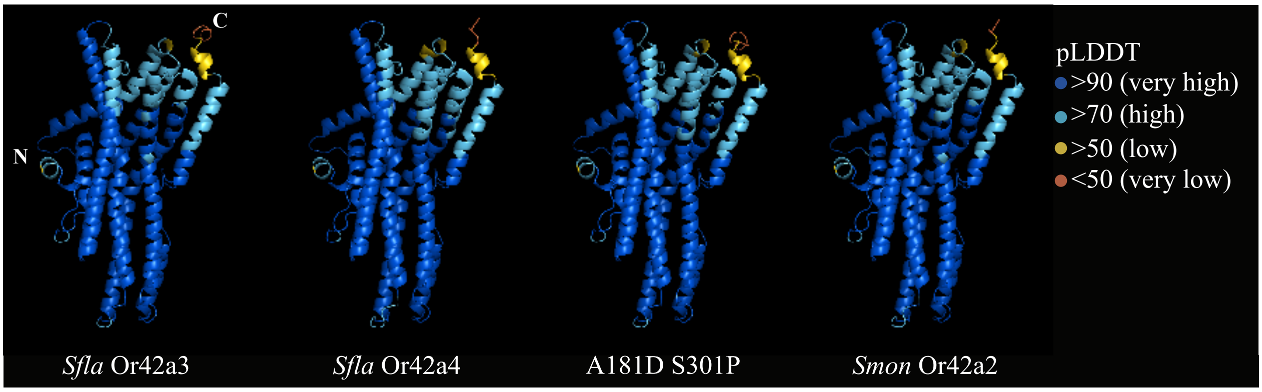

bioRxiv preprint doi: https://doi.org/10.1101/2024.10.08.617316; this version posted March 11, 2025. The copyright holder for this preprint (which was not certified by peer review) is the author/funder, who has granted bioRxiv a license to display the preprint in perpetuity. It is made available under aCC-BY 4.0 International license.

Supp. Figure 10

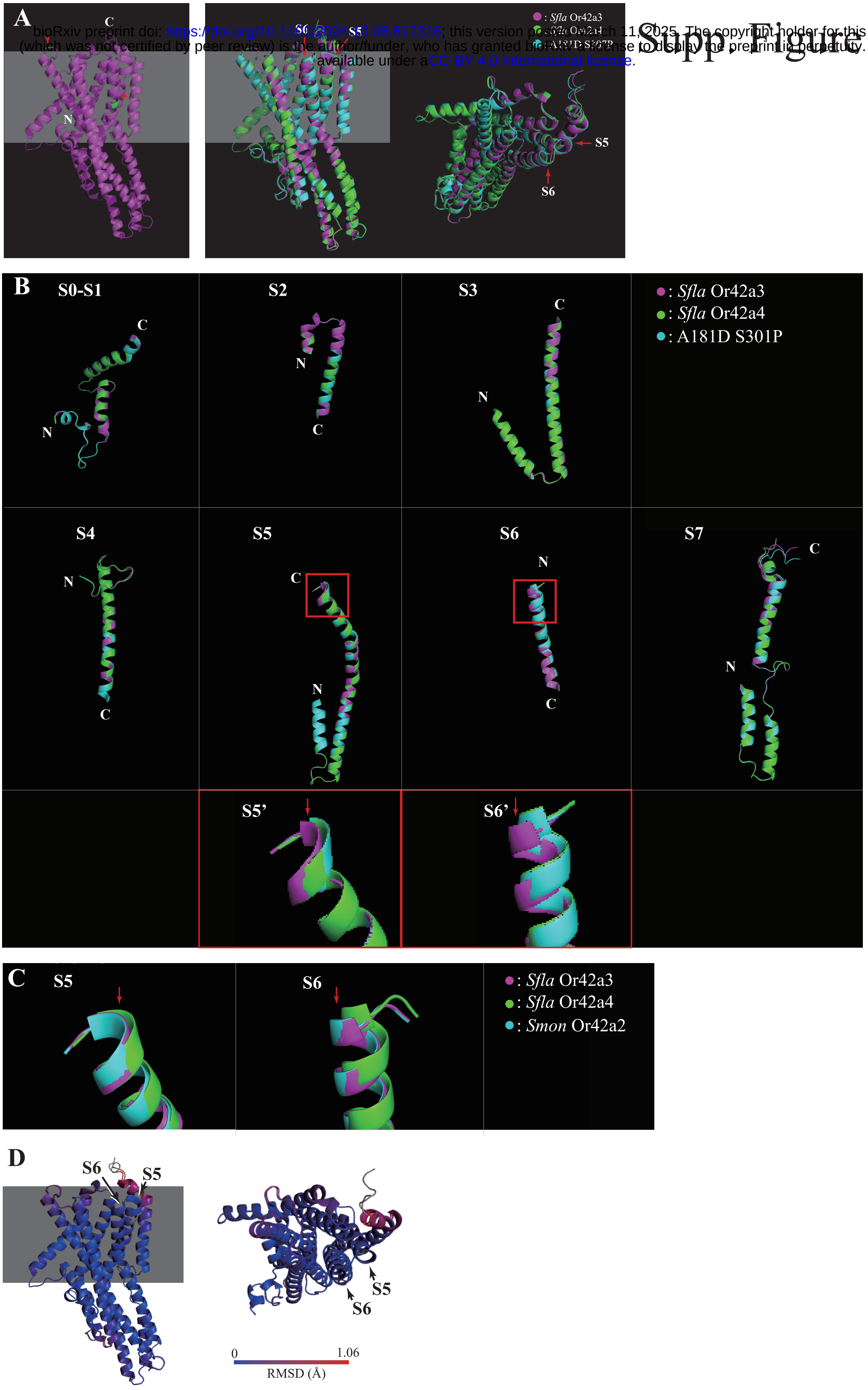

bioRxiv preprint doi: https://doi.org/10.1101/2024.10.08.617316; this version posted March 11, 2025. The copyright holder for this preprint (which was not certified by peer review) is the author/funder, who has granted bioRxiv a license to display the preprint in perpetuity. It is made available under aCC-BY 4.0 International license.

Supp. Figure 11

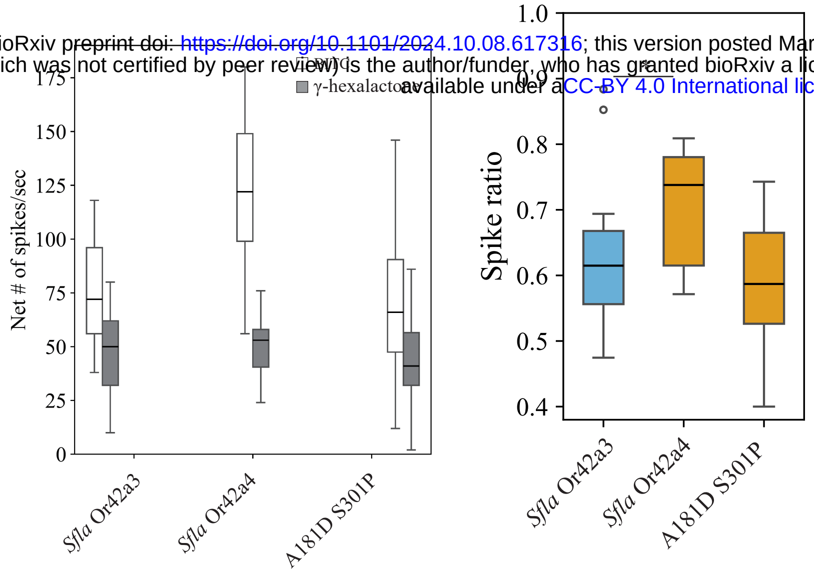

bioRxiv preprint doi: https://doi.org/10.1101/2024.10.08.617316; this version posted March 11, 2025. The copyright holder for this preprint (which was not certified by peer review) is the author/funder, who has granted bioRxiv a license to display the preprint in perpetuity. It is made available under aCC-BY 4.0 International license.

Supp. Figure 12

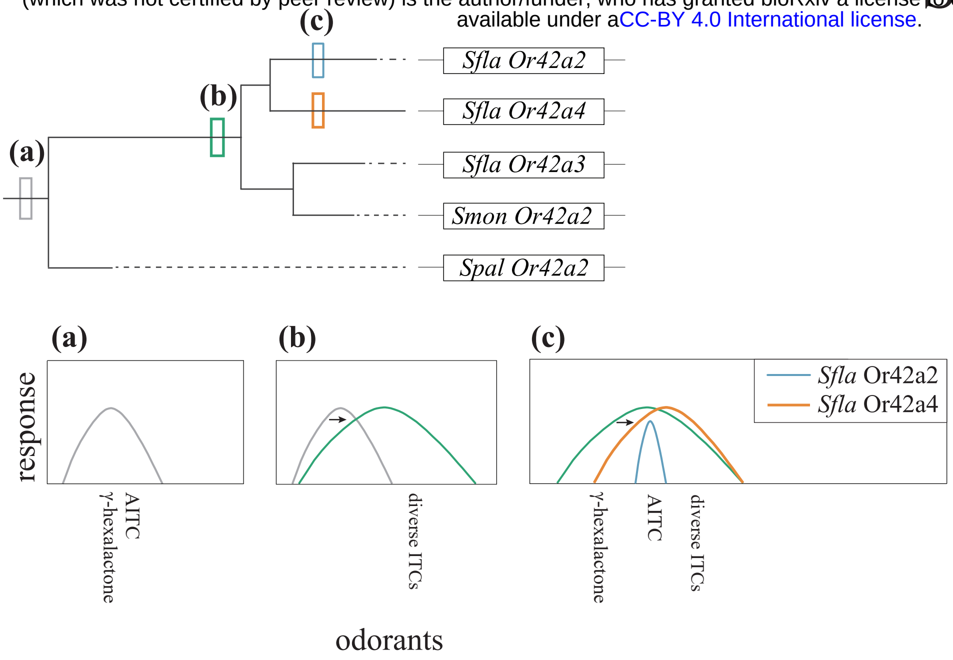

Supplement: Supplementary file 1 [file NIHPP2024.10.08.617316v2-supplement-14.pdf]
