# Supplementary material for: Odorant receptors tuned to isothiocyanates in Drosophila melanogaster and their evolutionary expansion in herbivorous relatives
Source: bioRxiv. 2024 Oct 11:2024.10.08.617316. Preprint. [Version 1] doi: 10.1101/2024.10.08.617316 (PMC11482750; doi:10.1101/2024.10.08.617316)
Supplement: Supplementary file 1 [file NIHPP2024.10.08.617316v1-supplement-1.pdf]

bioRxiv preprint doi: https://doi.org/10.1101/2024.10.08.617316; this version posted October 11, 2024. The copyright holder for this preprint (which was not certified by peer review) is the author/funder, who has granted bioRxiv a license to display the preprint in perpetuity. It is made available under aCC-BY 4.0 International license.

SUPPLEMENTARY MATERIAL AND METHODS:

Measurement of maxillary palps and body size

Two-to-ten-day old male *Dmel, Shsu, Spal,* and *Sfla* flies with their heads facing up and their bodies sideway were mounted and imaged using a commercial camera under a conventional microscope. The areas of the maxillary palps and the body (head, thorax excluding legs and wings, and abdomen) were measured using ImageJ v1.54.

bioRxiv preprint doi: https://doi.org/10.1101/2024.10.08.617316; this version posted October 11, 2024. The copyright holder for this preprint (which was not certified by peer review) is the author/funder, who has granted bioRxiv a license to display the preprint in perpetuity. It is made available under aCC-BY 4.0 International license.

Dilution series

trans-2-hexenal, AITC, IBITC, SBITC, and ethyl butyrate were diluted in mineral oil, and γ-hexalactone was diluted in DMSO to 10^{-5} to 10^{-2} vol/vol. Half maximal effective concentration (EC50) was generated by GraphPad Prism v10.2.1.

Construction of Or42a gene tree

Amino acid sequences of *Dmel Or42a,* and *Sfla Or42a1, Sfla Or42a2, Sfla Or42a3,* and *Sfla Or42a4* were obtained from Flybase (2024_01), and Goldman-Huertas et al., 2015. Blast searches were conducted to annotate *Shsu Or42a1, Spal Or42a1, Sgra Or42a1, Smon Or42a1, Sfla Or42a1, Shsu Or42a2, Spal Or42a2, Sgra Or42a2,* and *Smon Or42a2*, using *Sfla Or42a1* and *Sfla Or42a2* as query against the genomes of *S. hsui* (ASM1815282v1), *S. pallida* (ASM1815296v1), *S. graminum* (ASM1890183v1), and *S. montana* (ASM1890430v1). Amino acid sequences of *Shsu Or42a1, Spal Or42a1, Sgra Or42a1, Smon Or42a1, Sfla Or42a1, Shsu Or42a2, SpalOr4* (Katoh and Standley 2013)*2a2, Sgra Or42a2, Sfla Or42a2, Smon Or42a, Sfla Or42a2, Sfla Or42a3, Sfla Or42a4,* and *Dmel Or42a* were aligned by MAFFT v7.511 with JTT as scoring matrix for amino acid sequences with L-INS-i strategy. Maximum-likelihood gene tree with 1000 bootstrap cycles using the above sequences were constructed with RAxML (v8.2.19)(Stamatakis 2014). GAMMA model of rate heterogeneity was applied. CDS and alignment of *Or42a* are included in Supplementary files 12 and 13, respectively.

SUPPLEMENTARY FILES:

Supplementary file 1: Chemical list

Supplementary file 2: Control-subtracted net number of spikes/sec obtained from SSRs

Supplementary file 3: Survival assay data

Supplementary file 4: Consumption assay data

Supplementary file 5: Olfactory positional assay data

Supplementary file 6: Expression intensity from maxillary palp RNA-seq

Supplementary file 7: OSNs counts of RNA FISH

Supplementary file 8: Primer list

Supplementary file 9: AlphaFold2 (Colabfold) prediction in pdb format

bioRxiv preprint doi: https://doi.org/10.1101/2024.10.08.617316; this version posted October 11, 2024. The copyright holder for this preprint (which was not certified by peer review) is the author/funder, who has granted bioRxiv a license to display the preprint in perpetuity. It is made available under aCC-BY 4.0 International license.

Supplementary file 10: Pb sensilla counts by SSR

Supplementary file 11: Measurements of maxillary palp and body areas

Supplementary file 12: CDS of *Or42a* homologs in fasta format

Supplementary file 13: Alignment of *Or42a* homologs in fasta format

Figure 1

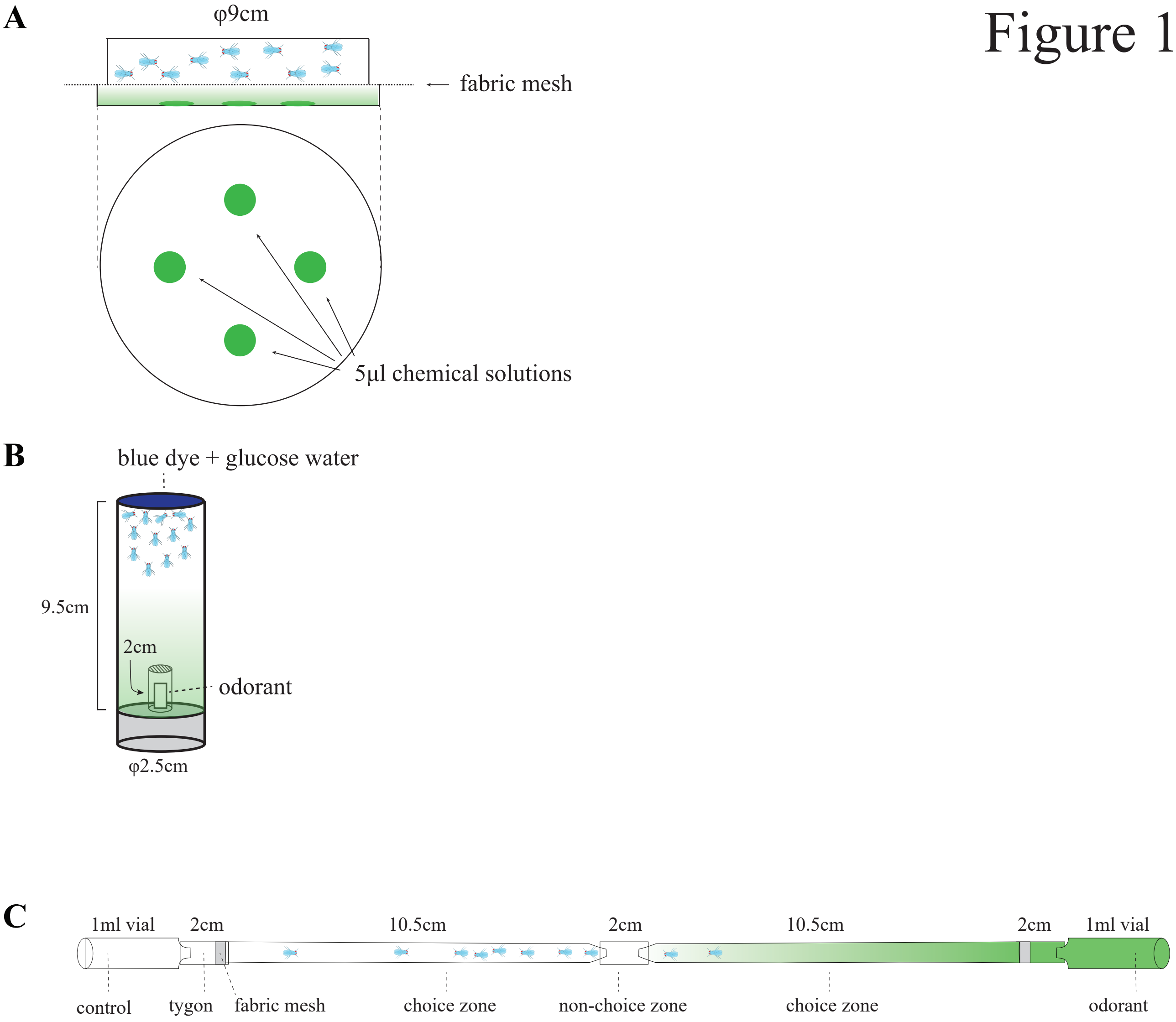
A
φ9cm
fabric mesh
5μl chemical solutions
B
blue dye + glucose water
9.5cm
2cm
odorant
φ2.5cm
C
1ml vial
2cm
10.5cm
2cm
10.5cm
2cm
1ml vial
control
tygon
fabric mesh
choice zone
non-choice zone
choice zone
odorant

Figure 2

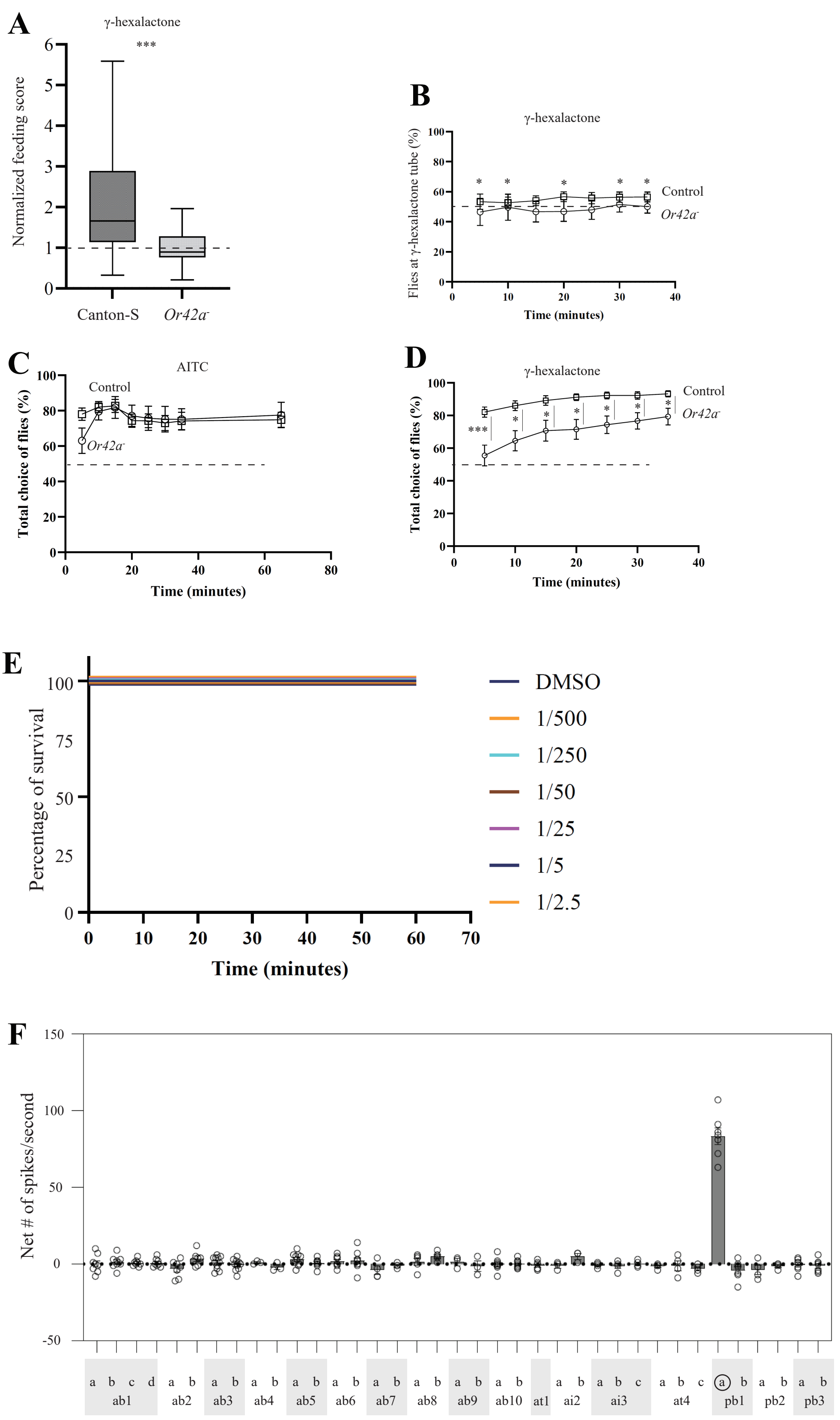
A
γ-hexalactone
***
Normalized feeding score
Canton-S
Or42a-
B
γ-hexalactone
Flies at γ-hexalactone tube (%)
Control
Or42a-
Time (minutes)
C
AITC
Total choice of flies (%)
Control
Or42a-
Time (minutes)
D
γ-hexalactone
Total choice of flies (%)
Control
Or42a-
Time (minutes)
E
Percentage of survival
Time (minutes)
DMSO
1/500
1/250
1/50
1/25
1/5
1/2.5
F
Net # of spikes/second
ab1
ab2
ab3
ab4
ab5
ab6
ab7
ab8
ab9
ab10
at1
ai2
ai3
at4
pb1
pb2
pb3

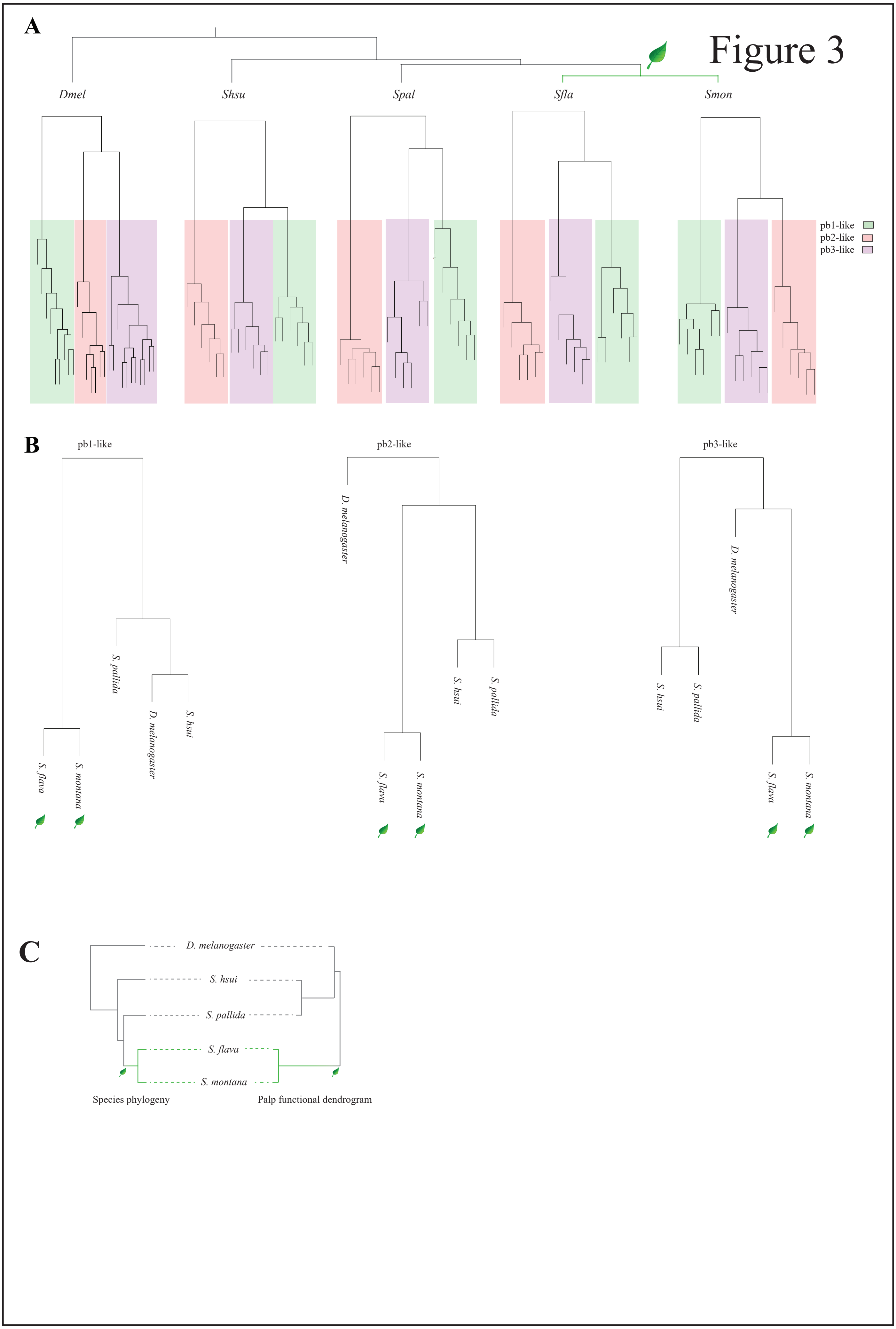

A
Figure 3
Dmel
Shsu
Spal
Sfla
Smon
pb1-like
pb2-like
pb3-like
B
pb1-like
S. flava
S. montana
S. pallida
D. melanogaster
S. hsui
pb2-like
D. melanogaster
S. flava
S. montana
S. hsui
S. pallida
pb3-like
S. hsui
S. pallida
D. melanogaster
S. flava
S. montana
C
D. melanogaster
S. hsui
S. pallida
S. flava
S. montana
Species phylogeny
Palp functional dendrogram

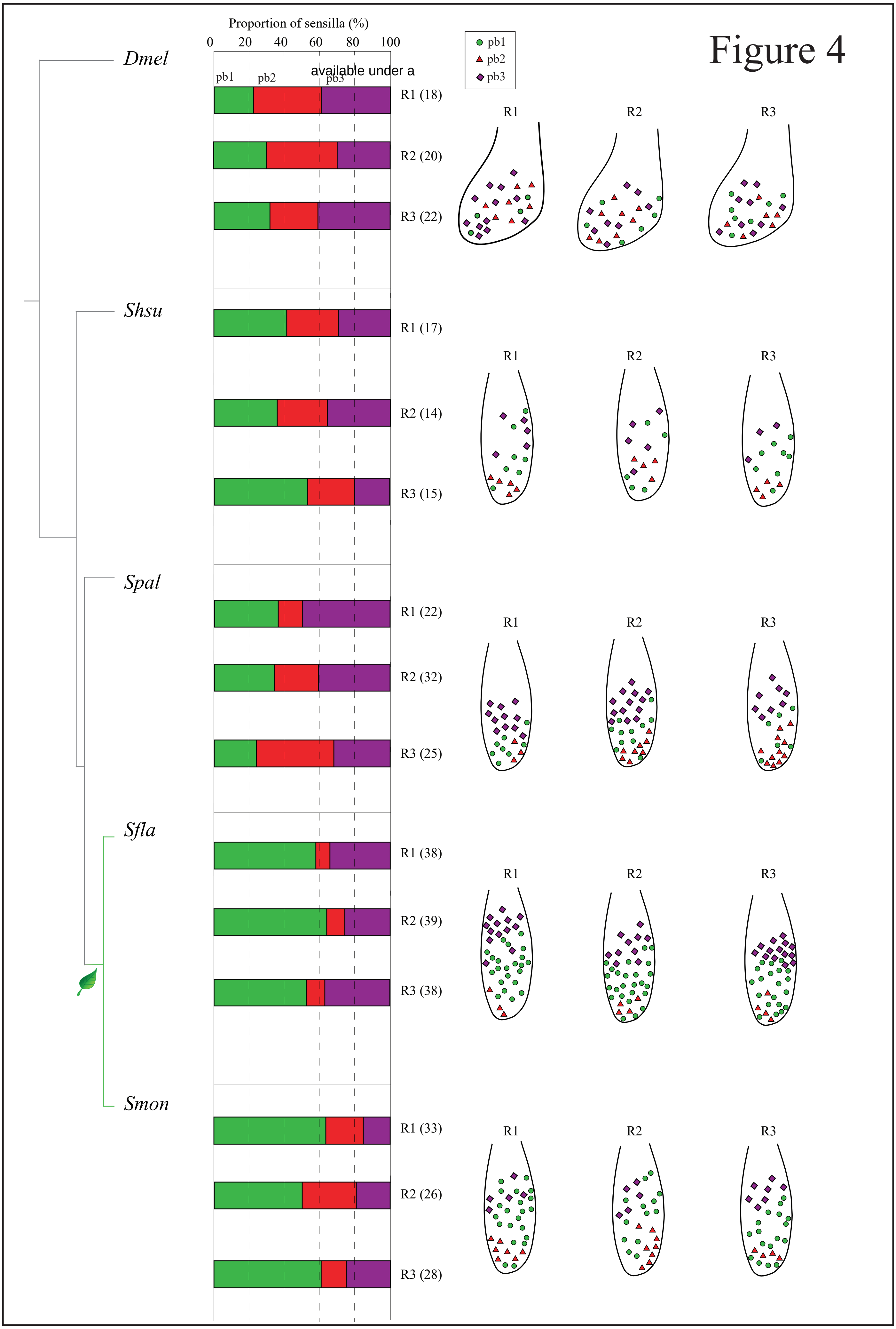
Figure 4
Proportion of sensilla (%)
0 20 40 60 80 100
available under a
pb1
pb2
pb3
Dmel
R1 (18)
R2 (20)
R3 (22)
Shsu
R1 (17)
R2 (14)
R3 (15)
Spal
R1 (22)
R2 (32)
R3 (25)
Sfla
R1 (38)
R2 (39)
R3 (38)
Smon
R1 (33)
R2 (26)
R3 (28)
R1
R2
R3

Figure 5

A

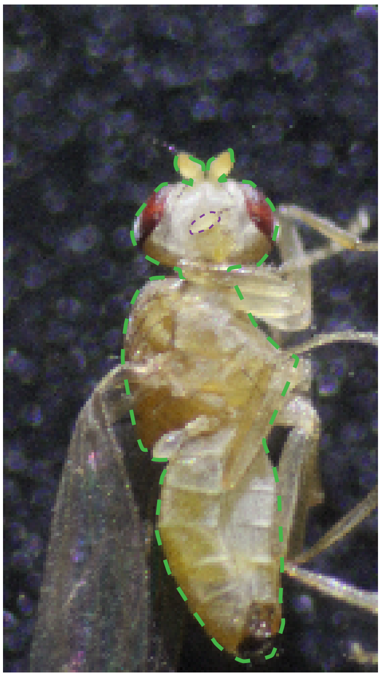

B

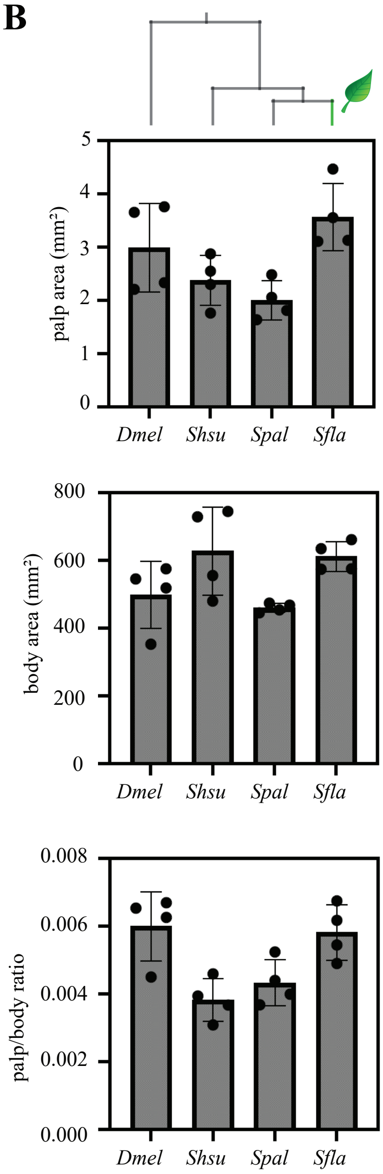

palp area (mm²)
body area (mm²)
palp/body ratio
Dmel
Shsu
Spal
Sfla

Figure 6

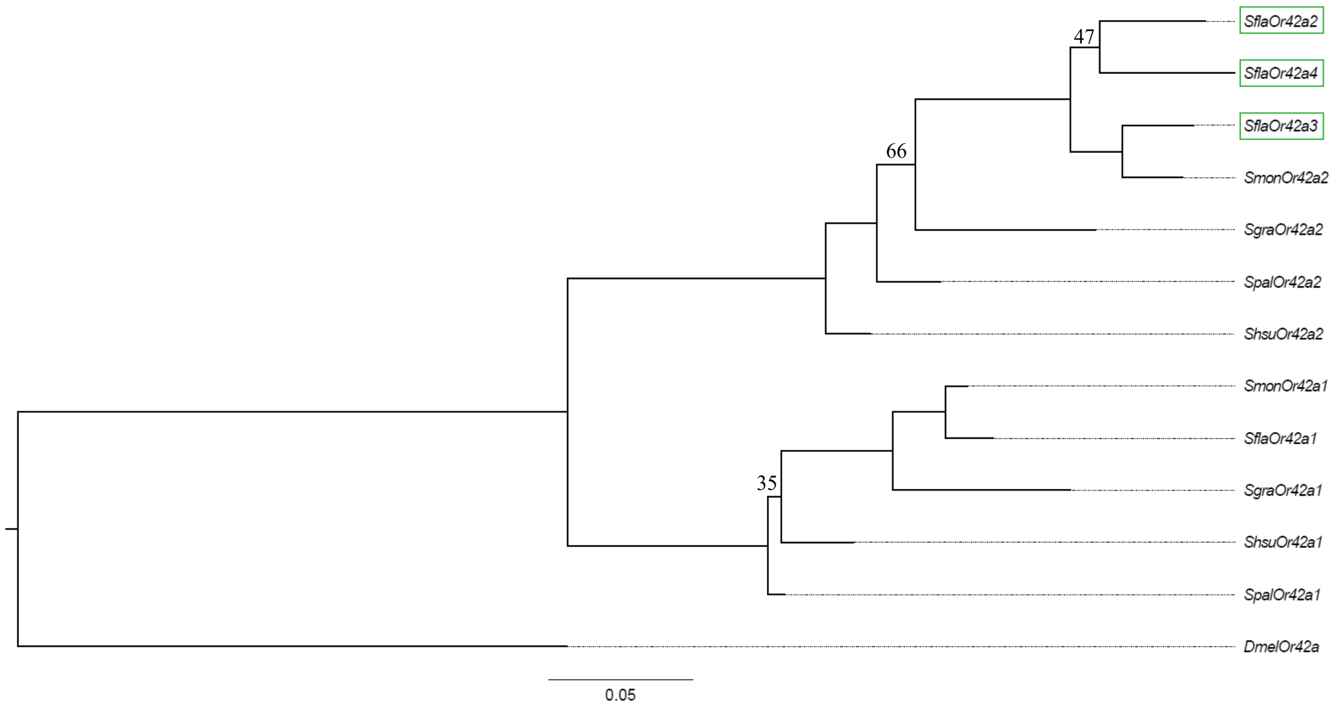

47
66
35
SflaOr42a2
SflaOr42a4
SflaOr42a3
SmonOr42a2
SgraOr42a2
SpalOr42a2
ShsuOr42a2
SmonOr42a1
SflaOr42a1
SgraOr42a1
ShsuOr42a1
SpalOr42a1
DmelOr42a
0.05

Figure 7

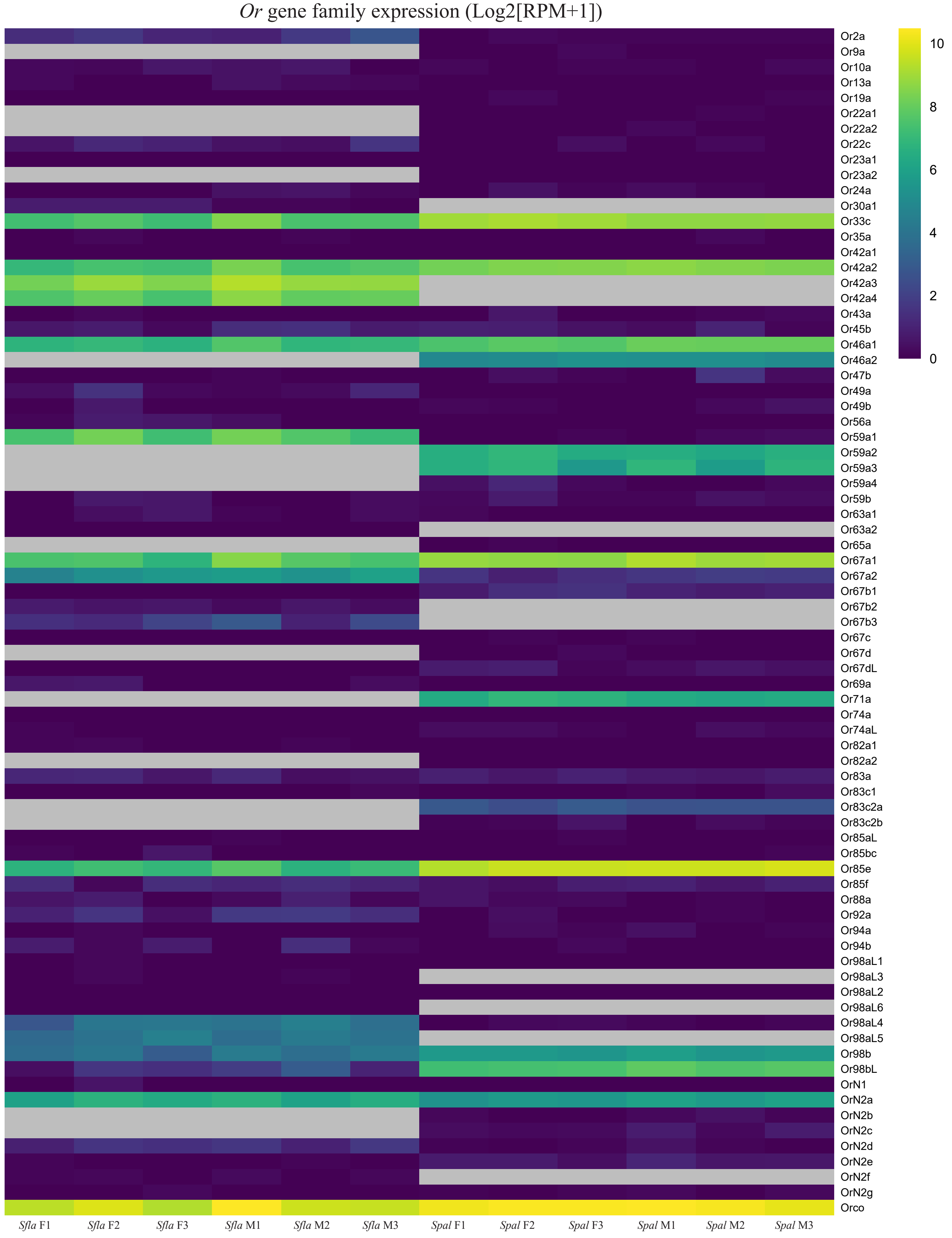
Or gene family expression (Log2[RPM+1])
Or2a
Or9a
Or10a
Or13a
Or19a
Or22a1
Or22a2
Or22c
Or23a1
Or23a2
Or24a
Or30a1
Or33c
Or35a
Or42a1
Or42a2
Or42a3
Or42a4
Or43a
Or45b
Or46a1
Or46a2
Or47b
Or49a
Or49b
Or56a
Or59a1
Or59a2
Or59a3
Or59a4
Or59b
Or63a1
Or63a2
Or65a
Or67a1
Or67a2
Or67b1
Or67b2
Or67b3
Or67c
Or67d
Or67dL
Or69a
Or71a
Or74a
Or74aL
Or82a1
Or82a2
Or83a
Or83c1
Or83c2a
Or83c2b
Or85aL
Or85bc
Or85e
Or85f
Or88a
Or92a
Or94a
Or94b
Or98aL1
Or98aL3
Or98aL2
Or98aL6
Or98aL4
Or98aL5
Or98b
Or98bL
OrN1
OrN2a
OrN2b
OrN2c
OrN2d
OrN2e
OrN2f
OrN2g
Orco
Sfla F1
Sfla F2
Sfla F3
Sfla M1
Sfla M2
Sfla M3
Spal F1
Spal F2
Spal F3
Spal M1
Spal M2
Spal M3
10
8
6
4
2
0

Figure 8

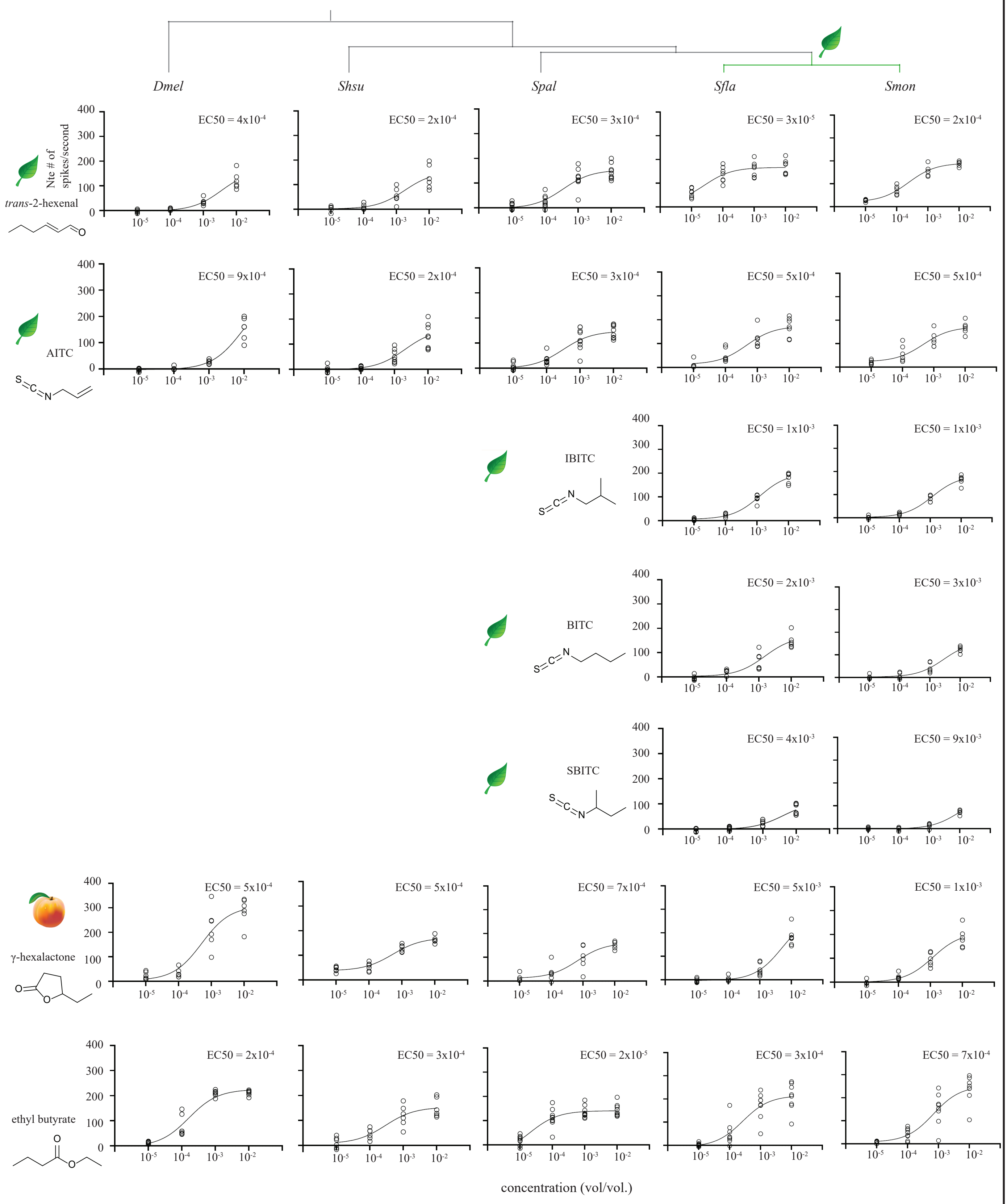

Dmel
Shsu
Spal
Sfla
Smon
Nte # of spikes/second
trans-2-hexenal
EC50 = 4x10⁻⁴
EC50 = 2x10⁻⁴
EC50 = 3x10⁻⁴
EC50 = 3x10⁻⁵
EC50 = 2x10⁻⁴
AITC
EC50 = 9x10⁻⁴
EC50 = 2x10⁻⁴
EC50 = 3x10⁻⁴
EC50 = 5x10⁻⁴
EC50 = 5x10⁻⁴
IBITC
EC50 = 1x10⁻³
EC50 = 1x10⁻³
BITC
EC50 = 2x10⁻³
EC50 = 3x10⁻³
SBITC
EC50 = 4x10⁻³
EC50 = 9x10⁻³
γ-hexalactone
EC50 = 5x10⁻⁴
EC50 = 5x10⁻⁴
EC50 = 7x10⁻⁴
EC50 = 5x10⁻³
EC50 = 1x10⁻³
ethyl butyrate
EC50 = 2x10⁻⁴
EC50 = 3x10⁻⁴
EC50 = 2x10⁻⁵
EC50 = 3x10⁻⁴
EC50 = 7x10⁻⁴
concentration (vol/vol.)

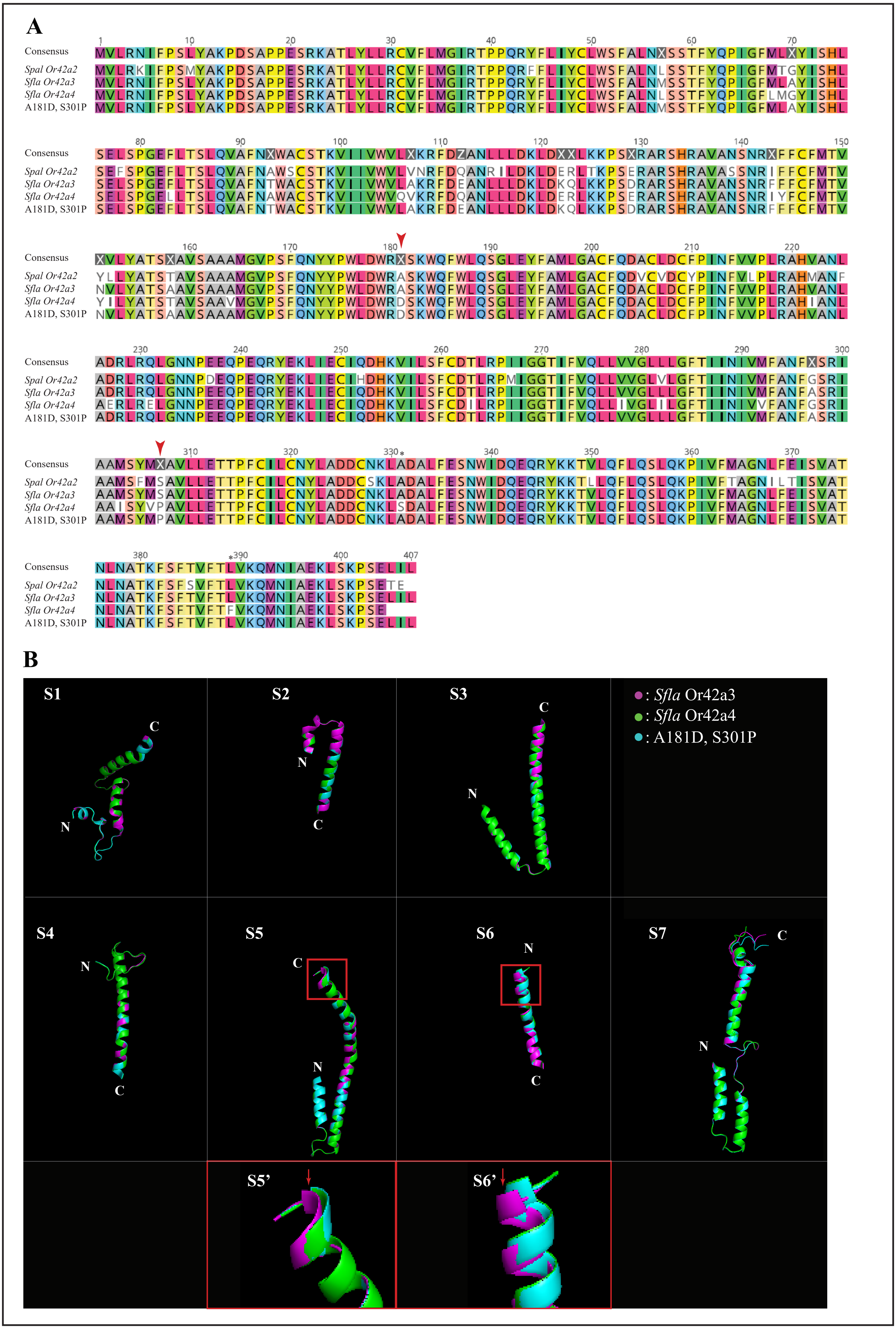
A
Consensus
Spal Or42a2
Sfla Or42a3
Sfla Or42a4
A181D, S301P
B
S1
S2
S3
S4
S5
S6
S7
S5'
S6'
N
C
●: Sfla Or42a3
●: Sfla Or42a4
●: A181D, S301P

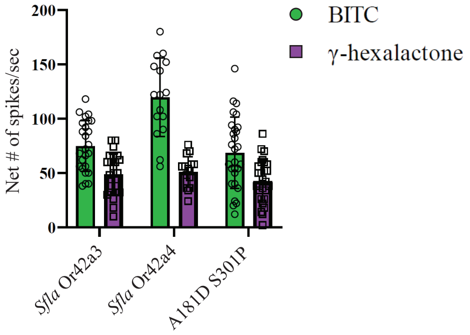

BITC
γ-hexalactone
Net # of spikes/sec
200
150
100
50
0
Sfla Or42a3
Sfla Or42a4
A181D S301P

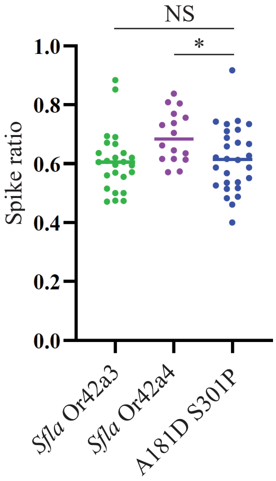

NS
*
Spike ratio
1.0
0.8
0.6
0.4
0.2
0.0
Sfla Or42a3
Sfla Or42a4
A181D S301P

Figure 10
